# Closely Related NDM-1-Encoding Plasmids from *Escherichia coli* and *Klebsiella pneumoniae* in Taiwan

**DOI:** 10.1371/journal.pone.0104899

**Published:** 2014-08-21

**Authors:** Chao-Ju Chen, Tsu-Lan Wu, Po-Liang Lu, Ying-Tsong Chen, Chang-Phone Fung, Yin-Ching Chuang, Jung-Chung Lin, L. Kristopher Siu

**Affiliations:** 1 Department of Internal Medicine, Kaohsiung Medical University Hospital, Kaohsiung, Taiwan; 2 College of Medicine, Kaohsiung Medical University, Kaohsiung, Taiwan; 3 Department of Clinical Pathology, Linkou Chang Gung Memorial Hospital, Taoyuan, Taiwan; 4 Institute of Genomics and Bioinformatics, National Chung Hsing University, Taichung, Taiwan; 5 Biotechnology Center, National Chung Hsing University, Taichung, Taiwan; 6 Institute of Molecular and Genomic Medicine, National Health Research Institutes, Miaoli, Taiwan; 7 Section of Infectious Diseases, Department of Medicine, Taipei Veterans General Hospital, Taipei, Taiwan; 8 Department of Internal Medicine and Medical Research, Chi Mei Medical Center, Tainan, Taiwan; 9 Department of Internal Medicine, Chi Mei Medical Center, Liouying, Tainan, Taiwan; 10 Division of Infectious Diseases and Tropical Medicine, Department of Internal Medicine, Tri-Service General Hospital, National Defense Medical Center, Taipei, Taiwan; 11 Institute of Microbiology and Immunology, National Defense Medical Center, Taipei, Taiwan; 12 Graduate Institute of Basic Medical Science, China Medical University, Taichung, Taiwan; 13 National Institute of Infectious Diseases and Vaccinology, National Health Research Institutes, Miaoli, Taiwan; Columbia University, College of Physicians and Surgeons, United States of America

## Abstract

**Objective:**

Two plasmids carrying *bla*_NDM-1_ isolated from carbapenem-resistant *Klebsiella pneumoniae* (CR-KP) and carbapenem-resistant *Escherichia coli* (CR-EC) were sequenced. CR-KP and CR-EC were isolated from two Taiwanese patients without travel histories.

**Methods:**

Complete sequencing of the plasmids (pLK75 and pLK78) was conducted using a shotgun approach. Annotation of the contigs was performed using the RAST Server, followed by manual inspection and correction.

**Results:**

These similar plasmids were obtained from two patients with overlapping stays at the same hospital. The pLK75 and pLK78 plasmids were 56,489-bp and 56,072-bp in length, respectively. Plasmid annotation revealed a common backbone similar to the IncN plasmid pR46. The regions flanking the *bla*_NDM-1_ genes in these plasmids were very similar to plasmid pNDM-HU01 in Japan, which contains a complex class 1 integron located next to an IS*CR1* element. The IS*CR1* element has been suggested to provide a powerful mechanism for mobilising antibiotic resistance genes.

**Conclusion:**

Two indigenous NDM-1-producing Enterobacteriaceae cases were identified for the first time in Taiwan, highlighting the alarming introduction of NDM-1-producing Enterobacteriaceae in this region.

## Introduction

New Delhi metallo-β-lactamase 1 (NDM-1) is a novel metallo-β-lactamase (MBL) that was first identified in 2009 [1]. Following the first case, NDM-1 positive bacteria have been detected in the UK, India, Pakistan and Bangladesh during 2008–2009 [2]. Since August 2010, NDM-1 isolates have been reported worldwide, including the United States and Canada, Europe, China, Japan, South Asia, Africa and Australia [3], [4]. Because it has rapidly spread worldwide, NDM-1 has become a global public health concern in recent years.

Plasmids encoding *bla*_NDM-1_ were initially observed in *Klebsiella pneumoniae* and *Escherichia coli*. However, these plasmids can be conjugatively transferred into other species, which facilitates the rapid dissemination of bla_NDM-1_ into various bacteria. The *bla*_NDM-1_ gene has been identified on different types of plasmids (IncA/C, IncF, IncL/M, or untypable), and plasmids carrying *bla*_NDM-1_ appear to have a broad host range [5], [6]. Many partial and complete plasmid sequences containing the *bla*_NDM-1_ gene have been published. Dissemination predominantly involves transfer of the *bla*_NDM-1_ gene among promiscuous plasmids and clonal outbreaks. Comparison of these plasmids shows that the regions neighbouring *bla*_NDM-1_ are quite variable, most likely reflecting multiple genetic modification events [6]. _ENREF_4Thus, an understanding of the genetic feature of various resistance determinants carried by these *bla*_NDM-1_ plasmids is important for understanding the evolution of *bla*_NDM-1_-carrying plasmids.

In Taiwan, the first case of NDM-1-producing Enterobacteriaceae colonisation was reported in 2010. The patient had been hospitalised for a gunshot injury in India and did not have symptoms or signs of infection during hospitalisation in Taipei [7]. Complete sequencing of the *bla*_NDM-1_-carrying plasmid, pKPX-1, revealed a hybrid replicon of IncR and IncF genes [5]. The first episode of infection caused by NDM-1-producing *K. pneumoniae* appeared soon thereafter in 2010. The patient received a kidney transplant in China and was admitted to a Taiwan hospital due to abdominal pain and dysuria, and an NDM-1-carrying *Klebsiella oxytoca* strain was isolated from a pelvic abscess [8]. The *bla*_NDM-1_-carrying plasmid, pKOX_NDM-1, was determined to carry a backbone with conjugation and replication genes similar to IncFIB and IncFII [6]. Here, we report the sequence annotation of pLK75 and pLK78, two similar *bla*_NDM-1_-encoding plasmids, from two Taiwanese patients without travel histories to foreign countries. Notably, these two NDM-1-producing isolates represent the first indigenous cases in Taiwan [9].

## Materials and Methods

### Patient Characteristics

The study protocol was reviewed and approved by the Institutional Review Board of Tri-Service General Hospital, National Defense Medical Center, Taipei, Taiwan. (TSGHIRB-100-05-205) Written informed consent to participants was provided in this study.

Patient 1 was an 82-year-old male with a history of hypertension, coronary artery disease, Parkinsonism, and pulmonary tuberculosis (under treatment for 3 months prior to the admission) and was a hepatitis B carrier. He was admitted for right lower lung pneumonia. CR-KP was isolated from his sputum specimen after two weeks of hospitalisation with antimicrobial therapy. The pneumonia was resolved after 3 days of ciprofloxacin followed by 6 days of ertapenem treatment.

Patient 2 was a 64-year-old female with a history of hypertension, diabetes mellitus, stroke, and end-stage renal disease with regular haemodialysis. She was admitted for respiratory failure and septic shock due to pneumonia. She received many classes of antimicrobial agents during the long hospitalisation period. CR-EC was isolated from the sputum for months after admission. However, she recovered after 14 days of cefepime treatment.

Because their hospital stays overlapped at the same hospital, a further genetic study and an outbreak investigation were performed.

### Conjugation of plasmid and antimicrobial susceptibility of *bla*_NDM-1_ donors and their transconjugants

Plasmid transfer was carried out by conjugation. A rifampin-resistant strain of *E. coli* (strain JP-995) was used as the recipient [10]. The recipient and donor strains were separately inoculated into brain heart infusion broth (Oxoid Ltd., Basingstoke, England) and incubated at 37°C for 4 h. The cells were then mixed at a ratio of 1∶10 (by volume) and incubated overnight at 37°C. A 0.1-ml sample of the overnight broth mixture was then spread onto a MacConkey agar plate containing rifampin (100 µg/ml) and imipenem (1 µg/ml). We did the PCR for transconjugants to confirm the transfer of *bla*_NDM-1_. Only one plasmid was observed in each transconjugants.

### Complete sequencing and annotation of plasmids pLK75 from CR-EC and pLK78 from CR-KP

Complete sequencing of the plasmids was conducted using a shotgun approach; sequencing of 8-kb pair-end and shotgun libraries prepared from purified plasmid DNA was carried out using a 454 GS Junior (Roche, US). Gap filling between the contigs was accomplished by adding Sanger reads with the aid of Consed [11]. Annotation of the contigs was performed using the RAST Server [12], followed by manual inspection and correction. The complete nucleotide sequences and annotations of plasmids pLK75 and pLK78 were submitted to GenBank and assigned the sequence accession numbers KJ440076 and KJ440075, respectively (sequence S1 and S2). Multilocus sequence typing (MLST) performed for *K. pneumoniae* isolate was according to Diancourt *et al.* [13]. Allele sequences and sequence types were verified at http://www.pasteur.fr/recherche/genopole/PF8/mlst/Kpneumoniae.html. MLST performed for *E. coli* isolate was according to the protocol described on the *E. coli* MLST website (http://mlst.warwick.ac.uk/mlst/dbs/Ecoli/documents/primersColi_html). The allele sequences and sequence types were verified at the http://mlst.warwick.ac.uk/mlst/dbs/Ecoli website.

### Antimicrobial susceptibility testing

The MICs of antimicrobial agents were determined using the broth microdilution test according to the recommendations from the Clinical and Laboratory Standards Institute [14]. The following antimicrobial agents were used: ampicillin, cefazolin, cefotaxime, ceftazidime, cefepime, aztreonam, cefoxitin, ertapenem, imipenem, meropenem, doripenem, ciprofloxacin, gentamicin, amikacin, colistin, tigecycline and trimethoprim-sulfamethoxazole (SXT).

## Results and Discussion

### The first indigenous NDM-1-producing bacteria in Taiwan

To our knowledge, only four NDM-1-positive Enterobacteriaceae isolates have been reported in Taiwan to date. These four isolates were recovered from two Taiwanese patients with travel histories to foreign countries [15]. Because the patients in the present study had no such travel history, these 2 NDM-1-producing bacteria are thought to be indigenous.

### The patients' characteristics of two NDM-1-producing strains

Tables 1 and 2 showed the patient clinical features and antimicrobial susceptibility testing results of the *bla*_NDM-1_-carrying isolates. Both of the patients were elderly and immunocompromised. Both patients stayed in the same hospital for pneumonia, and NDM-1-producing bacteria were cultured from their sputum specimens after usage of several broad-spectrum antibiotics. These two isolates were hospital-acquired and suggested to be colonisation isolates because clinical improvement was achieved without effective antimicrobial therapy and no CR-EC or CR-KP was isolated from other sources of the patients. With strict infection and contact control practices, no further NDM-1-producing Enterobacteriaceae isolates were identified for one year. The isolation dates of these two patients' NDM-1-producing Enterobacteriaceae were within in one week of each other. Moreover, their patterns of antimicrobial susceptibility were quite similar. The transconjugants with a plasmid harbouring NDM-1 showed resistance to all β-lactams. The transconjugants converted from ciprofloxacin-resistant to ciprofloxacin-susceptible, indicating non-plasmid-mediated resistance in the donor strains. Both NDM-1-carrying *E. coli* and *K. pneumoniae* strains were susceptible to amikacin, colistin, and tigecycline (Table 2).

**Table 1:** Clinical features of patients carrying *bla*_NDM-1_.

Patient	1 (pLK-75, CR-KP)	2 (pLK-78, CR-EC)
Age	82	64
Gender	Male	Female
Underlying disease	HTN, CAD, pulmonary TB, HBV, CKD, Parkinsonism	HTN, DM, stroke and ESRD with HD, schizophrenia
Admission period	2012/09/05∼2012/10/10	2012/05/28∼2012/10/24
Clinical diagnosis	Pneumonia	Pneumonia, septic shock, respiratory failure
NDM-1 bacteria isolation date	2012/09/24	2012/09/29
Source	Sputum	Sputum
Antibiotics	Ceftriaxone (9/4∼9/5), Piperacillin/Tazobactam (9/6∼9/20), Ciprofloxacin (9/24∼9/26), Ertapenem (9/27∼10/2), Isoniazid (6/28∼), Rifampin (6/28∼)	Piperacillin/Tazobactam (9/4∼9/19), Levofloxacin (9/14∼9/27), Linezolid (9/19∼10/1) Imipenem/Cilastatin (9/28∼10/2), Ceftriaxone (10/3∼10/11), Daptomycin (10/3∼10/17), Cefepime (10/12∼10/26)
Outcome	Improved	Improved

Abbreviations: HTN, hypertension; CAD, coronary artery disease; TB, tuberculosis; HBV, hepatitis B; CKD, chronic kidney disease; ESRD, end-stage renal disease; HD, haemodialysis.

**Table 2:** Antimicrobial susceptibility testing among *bla*_NDM-1_-carrying isolates and their transconjugants.

Antibiotics	Patient 1		Patient 2	
	*E. coli*	Transconjugant	*K. pneumoniae*	Transconjugant
Ampicillin	≥32 µg/ml	≥32 µg/ml	≥32 µg/ml	≥32 µg/ml
Cefazolin	≥32 µg/ml	≥32 µg/ml	≥32 µg/ml	≥32 µg/ml
Cefotaxime	≥64 µg/ml	≥64 µg/ml	≥64 µg/ml	≥64 µg/ml
Ceftazidime	≥32 µg/ml	≥32 µg/ml	≥32 µg/ml	≥32 µg/ml
Cefepime	≥32 µg/ml	≥32 µg/ml	≥32 µg/ml	≥32 µg/ml
Aztreonam	≥32 µg/ml	≥32 µg/ml	≥32 µg/ml	≥32 µg/ml
Cefoxitin	≥32 µg/ml	≥32 µg/ml	≥32 µg/ml	≥32 µg/ml
Ertapenem	≥8 µg/ml	≥8 µg/ml	≥8 µg/ml	≥8 µg/ml
Imipenem	≥8 µg/ml	≥8 µg/ml	≥8 µg/ml	≥8 µg/ml
Meropenem	≥8 µg/ml	≥8 µg/ml	≥8 µg/ml	4 µg/ml
Doripenem	≥8 µg/ml	≥8 µg/ml	≥8 µg/ml	≥8 µg/ml
Ciprofloxacin	≥4 µg/ml	≤0.06 µg/ml	≥4 µg/ml	≤0.06 µg/ml
Gentamicin	≥16 µg/ml	≥16 µg/ml	≤1 µg/ml	≤1 µg/ml
Amikacin	≤4 µg/ml	≤4 µg/ml	≤4 µg/ml	≤4 µg/ml
Colistin	≤0.5 µg/ml	≤0.5 µg/ml	1 µg/ml	≤0.5 µg/ml
Tigecycline	0.5 µg/ml	≤0.25 µg/ml	1 µg/ml	≤0.25 µg/ml
SXT^a^	≥4 µg/ml	2 µg/ml	≥4 µg/ml	2 µg/ml

a SXT: Trimethoprim/sulfamethoxazole.

Trimethoprim/sulfamethoxazole MICs are presented according to the concentration of trimethoprim.

### Genetic features

**Figure 1:**
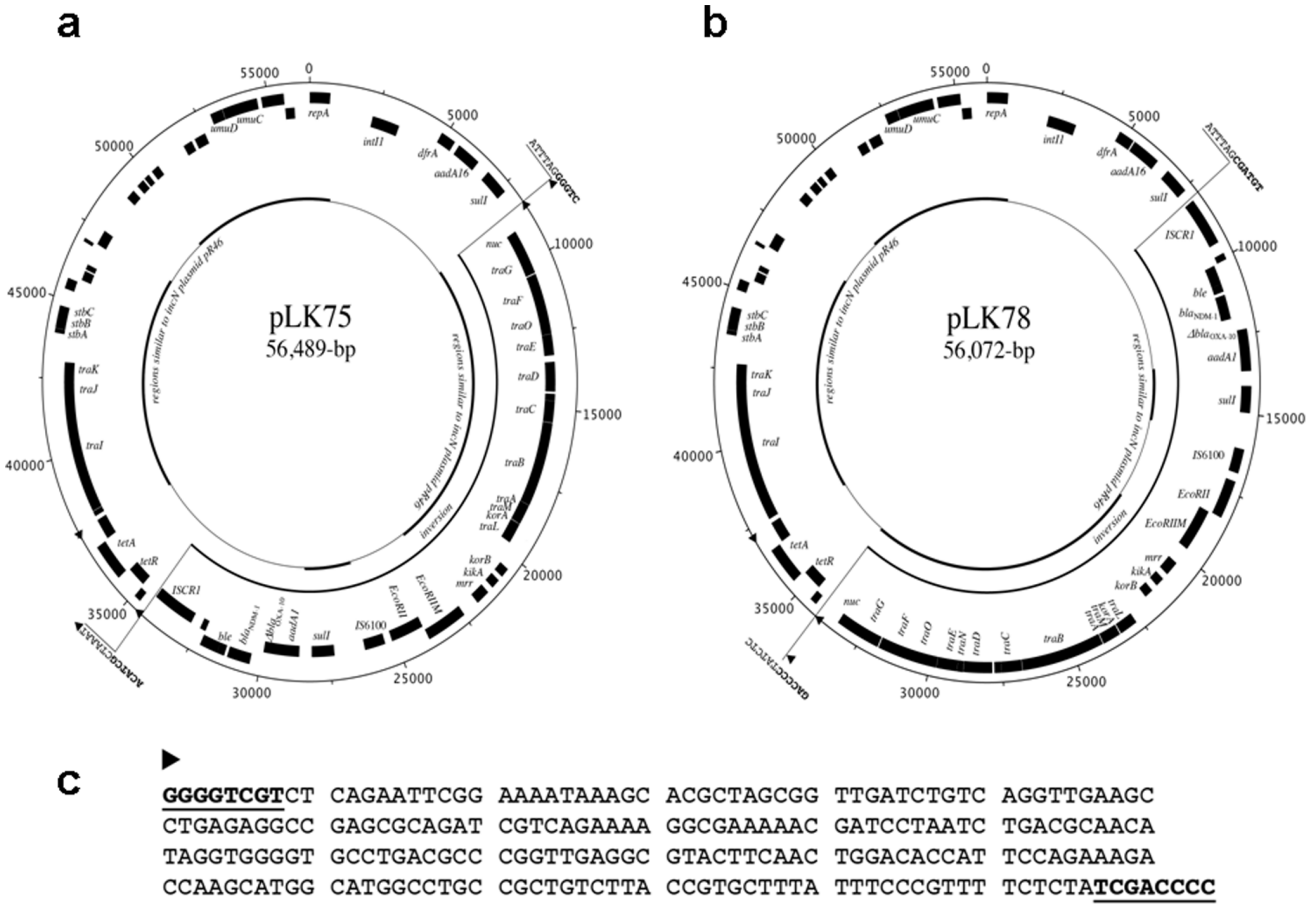
Schematic maps of (a) pLK75 and (b) pLK78. The black blocks on the outer circle are the coding DNA sequences of the positive strand, and the black blocks on the inner circle are the coding DNA sequences of the negative strand. The remarkable features are indicated. Consensus regions similar to other plasmids/replicons are marked with thick black lines inside the circles, with non-consensus sections between the conserved regions shown by thin lines. The 26-kb inversion regions are marked in the inner circles, and the flanking sequences are shown. The sequences at both ends of the inversion region are shown in bold. The positions of the 244-bp Tn*3*-like repeats (c) are marked by black arrowheads on the circles. The 244-bp repeat sequence is shown in (c). The putative inverted repeats flanking this sequence are shown in bold with an underline.

Complete sequencing was performed for the two circular *bla*_NDM-1_ plasmids: pLK75 from *E. coli* and pLK78 from *K. pneumoniae*. The pLK75 and pLK78 plasmids were 56,489-bp and 56,072-bp in length, respectively. Annotation of the plasmids revealed a common backbone similar to the IncN plasmid pR46 [16] (Figure 1a, 1b). The majority of plasmid pR46 sequences (∼72% of 50,969 bp) were preserved in both pLK75 and pLK78, including the *repA* gene for plasmid replication, *tra* genes for conjugal transfer, and genes responsible for plasmid stability. Although the *tetA*/*tetR* genes responsible for tetracycline resistance were also identified in these plasmids, a gene cluster responsible for arsenite resistance in pR46 was not found in pLK75 and pLK78. Plasmids pLK75 and pLK78 were similar to each other but contained a 26-kb inversion region located next to the *sul1* gene of a class 1 integron downstream of the *repA* replication origin (Figure 1a, 1b). At the other end of the inversion region were *tetA*/*tetR* tetracycline resistance genes flanked by two 244-bp repeat sequences (Figure 1c). This 244-bp repeat region was flanked by inverted repeats (IRs) similar to the IRs of the Tn*3* family (Figure 1c). Compared to pLK78, an additional copy of the 244-bp repeat was identified at the other end of the inversion region in pLK75 (Figure 1a). It is likely that the 244-bp Tn*3*-like repeat sequences may have facilitated the inversion of the 26-kb region. Both of the plasmids carried additional antimicrobial resistance genes to pR46, including *bla*_NDM-1_ (metallo-β-lactamase NDM-1), *sul1* (sulphonamide resistance gene), *aadA1* (aminoglycoside resistance gene), *aadA16* (streptomycin-spectinomycin resistance gene), and *ble* (bleomycin resistance gene). The IncN plasmid has been shown to encode clinically important resistance determinants, such as *bla*_CTX-M_, *bla*_IMP_, *bla*_NDM_, and *bla*_KPC_. This type of plasmid is known to be involved in the transmission of VIM-1, KPC-2, CTX-M-1, and NDM-1 among *K. pneumoniae* isolates and has also been prevalent in *E. coli* [17]. Highly efficient transmission of these plasmids may explain the diversity and worldwide spread of *bla*_NDM-1_-carrying Enterobacteriaceae [18].

### Genetic environment of *bla*_NDM-1_ in pLK75 and pLK78

**Figure 2:**
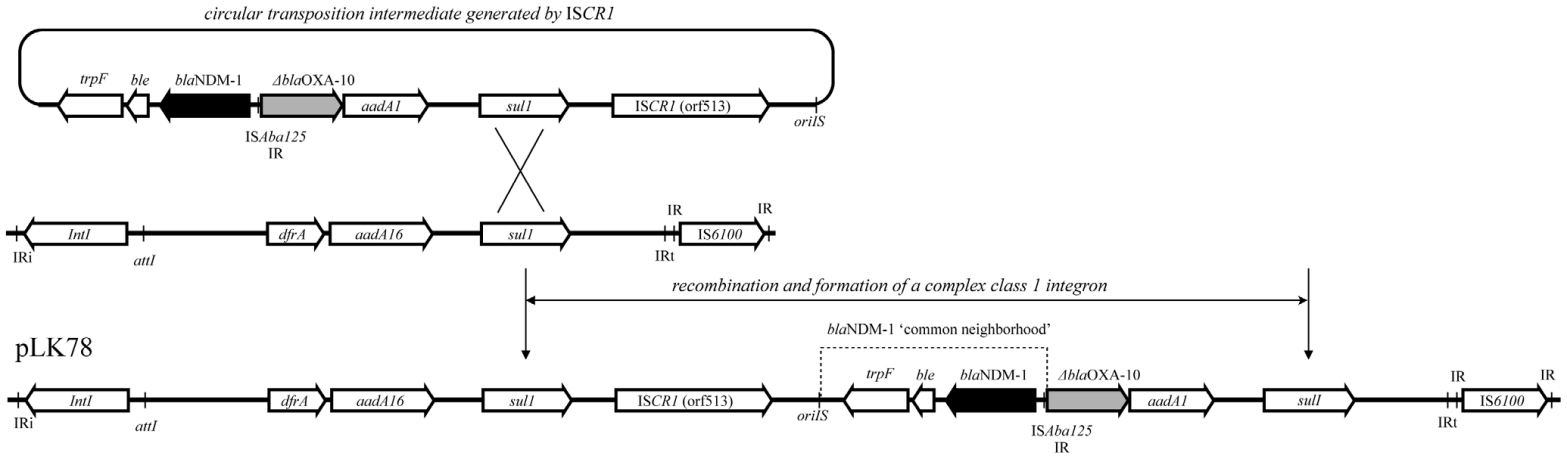
Integration of the IS*CR1* element and its nearby sequences, including the blaNDM-1 gene region, into a class 1 integron. The circular transposition intermediate generated by IS*CR1* is shown above. Integration at the *sul1* locus of a class 1 integron resulted in the genetic environment identified as flanking the *bla*_NDM-1_ ‘common neighbourhood’ region in pLK75 and pLK78 (pLK78 is shown here). The remarkable features are indicated: IRs, inverted repeats; *IntI*, integrase gene of class 1 integron; IS*CR1* (*orf513*), integrase gene of IS*CR1*; *oriIS*, origin of replication of the IS*CR* element.

The flanking region of the *bla*_NDM-1_ gene in these plasmids was very similar (∼100% identity) to plasmid pNDM-HU01, of which the partially sequenced region, including *qacEdelta1*, *aadA*, *bla*_NDM-1_, *ble*, *trpF*, IS*CR1*, and *sul1*, was reported from an *E. coli* isolate in Japan (GenBank AB769140). Additionally, the immediate region containing *bla*_NDM-1_, including the upstream intergenic region of a truncated IS*Aba*125, *bla*_NDM-1_, the *ble* bleomycin resistance gene, and the *trpF* phosphoribosylanthranilate isomerase gene, represents a ‘common neighbourhood’ to *bla*_NDM-1_, which is identical in almost all of the reported *bla*_NDM-1_-containing sequences thus far [5], [19]. This region was located next to an IS*CR1* element in both plasmids. The genetic environment of the *bla*_NDM-1_ region represents a typical complex integron in pLK78 (Figure 2), with the *bla*_NDM-1_ ‘common neighbourhood’ region and the adjacent IS*CR1* element located between two *sul1* genes associated with a nearby class 1 integron (Figure 2). In pLK78, the gene annotation revealed a complex class 1 integron near the *bla*_NDM-1_. While the two plasmids are almost identical, the complex class1 integron structure in pLK75 is truncated due to a 26-kb inversion. Therefore it is likely that pLK75 and pLK78 were derived from the same molecular ancestor, but pLK75 was altered subsequently by the inversion, which resulted in disruption of the integron structure.

IS*CR1* elements may transpose adjacent DNA sequences by a mechanism termed rolling-circle transposition and are responsible for the mobilisation of antibiotic resistance genes [20], [21]. It is likely that the IS*CR1* element mediated the acquisition of the *bla*_NDM-1_ common region to the class 1 integron, presumably through recombination at the *sul1* locus, to form the complex class 1 integron structure in pLK78 (Figure 2). Previous analysis reveal that there is an advantage of *bla*_NDM-1_ embedded within integron IS*CR1* complex since this element is known to be widely associated with the spreading of antibiotic resistance genes among bacteria [22]. Acquisition of *bla*_NDM-1_ to the IncN replicon, presumable with the aid of IS*CR1* element, may have bolstered the spreading of the resistance among *K. pneumoniae* and *E. coli*.

In conclusion, we obtained the first indigenous NDM-1-producing *E. coli* and *K. pneumoniae* isolates from patients in Taiwan without travel histories. Comparative analyses of the *bla*_NDM-1_-encoding plasmids from *E. coli* and *K. pneumoniae* revealed the importance of *tra* genes and IS*CR*1. Considering the clinical association and genetic information, the tight connection of these two carbapenem-resistant Enterobacteriaceae is not surprising. Clinicians should be aware of the possible emergence of an epidemic of carbapenem-resistant Enterobacteriaceae related to the level of plasmid transmission.

## Supporting Information

Sequence S1 **Complete nucleotide sequences and annotations of plasmids pLK75.**
(DOCX)

Sequence S2 **Complete nucleotide sequences and annotations of plasmids pLK78.**
(DOCX)

## References

[1] Yong D, Toleman MA, Giske CG, Cho HS, Sundman K, et al (2009) Characterization of a new metallo-beta-lactamase gene, *bla* _NDM-1_, and a novel erythromycin esterase gene carried on a unique genetic structure in *Klebsiella pneumoniae* sequence type 14 from India. Antimicrob Agents Chemother 53: 5046–5054. 19770275 10.1128/AAC.00774-09 PMC2786356

[2] Kumarasamy KK, Toleman MA, Walsh TR, Bagaria J, Butt F, et al (2010) Emergence of a new antibiotic resistance mechanism in India, Pakistan, and the UK: a molecular, biological, and epidemiological study. Lancet Infect Dis 10: 597–602. 20705517 10.1016/S1473-3099(10)70143-2 PMC2933358

[3] Rolain JM, Parola P, Cornaglia G (2010) New Delhi metallo-beta-lactamase (NDM-1): towards a new pandemia? Clin Microbiol Infect 16: 1699–1701. 20874758 10.1111/j.1469-0691.2010.03385.x

[4] Nordmann P, Poirel L, Walsh TR, Livermore DM (2011) The emerging NDM carbapenemases. Trends Microbiol 19: 588–595. 22078325 10.1016/j.tim.2011.09.005

[5] Chen YT, Lin AC, Siu LK, Koh TH (2012) Sequence of closely related plasmids encoding *bla* _NDM-1_ in two unrelated *Klebsiella pneumoniae* isolates in Singapore. PLoS One 7: e48737. 23139815 10.1371/journal.pone.0048737 PMC3490853

[6] Huang TW, Chen TL, Chen YT, Lauderdale TL, Liao TL, et al (2013) Copy Number Change of the NDM-1 sequence in a multidrug-resistant *Klebsiella pneumoniae* clinical isolate. PLoS One 8: e62774. 23658651 10.1371/journal.pone.0062774 PMC3639163

[7] Wu HS, Chen TL, Chen IC, Huang MS, Wang FD, et al (2010) First identification of a patient colonized with *Klebsiella pneumoniae* carrying *bla* _NDM-1_ in Taiwan. J Chin Med Assoc 73: 596–598. 21093828 10.1016/S1726-4901(10)70129-5

[8] Lai CC, Lin TL, Tseng SP, Huang YT, Wang JT, et al (2011) Pelvic abscess caused by New Delhi metallo-beta-lactamase-1-producing *Klebsiella oxytoca* in Taiwan in a patient who underwent renal transplantation in China. Diagn Microbiol Infect Dis 71: 474–475. 22083082 10.1016/j.diagmicrobio.2011.09.004

[9] Ma L, Siu LK, Lin JC, Wu TL, Fung CP, et al (2013) Updated molecular epidemiology of carbapenem-non-susceptible *Escherichia coli* in Taiwan: first identification of KPC-2 or NDM-1-producing E. coli in Taiwan. BMC Infect Dis 13: 599. 24354657 10.1186/1471-2334-13-599 PMC3878139

[10] Siu LK, Ho PL, Yuen KY, Wong SS, Chau PY (1997) Transferable hyperproduction of TEM-1 beta-lactamase in *Shigella flexneri* due to a point mutation in the pribnow box. Antimicrob Agents Chemother 41: 468–470. 9021210 10.1128/aac.41.2.468 PMC163732

[11] Gordon D, Abajian C, Green P (1998) Consed: a graphical tool for sequence finishing. Genome Res 8: 195–202. 9521923 10.1101/gr.8.3.195

[12] Aziz RK, Bartels D, Best AA, DeJongh M, Disz T, et al (2008) The RAST Server: rapid annotations using subsystems technology. BMC Genomics 9: 75. 18261238 10.1186/1471-2164-9-75 PMC2265698

[13] Diancourt L, Passet V, Verhoef J, Grimont PA, Brisse S (2005) Multilocus sequence typing of *Klebsiella pneumoniae* nosocomial isolates. J Clin Microbiol 43: 4178–4182. 16081970 10.1128/JCM.43.8.4178-4182.2005 PMC1233940

[14] CLSI. Clinical and Laboratory Standards Institute. Performance standards for antimicrobial susceptibility testing; 20th informational supplement M100-S23. Wayne, PA: Clinical and Laboratory Standards Institute, 2013.

[15] Wang SJ, Chiu SH, Lin YC, Tsai YC, Mu JJ (2013) Carbapenem resistant Enterobacteriaceae carrying New Delhi metallo-beta-lactamase gene (NDM-1) in Taiwan. Diagn Microbiol Infect Dis 76: 248–249. 23518181 10.1016/j.diagmicrobio.2013.02.003

[16] Hall RM, Vockler C (1987) The region of the IncN plasmid R46 coding for resistance to beta-lactam antibiotics, streptomycin/spectinomycin and sulphonamides is closely related to antibiotic resistance segments found in IncW plasmids and in Tn21-like transposons. Nucleic Acids Res 15: 7491–7501. 2821509 10.1093/nar/15.18.7491 PMC306263

[17] Humphrey B, Thomson NR, Thomas CM, Brooks K, Sanders M, et al (2012) Fitness of *Escherichia coli* strains carrying expressed and partially silent IncN and IncP1 plasmids. BMC Microbiol 12: 53. 22475035 10.1186/1471-2180-12-53 PMC3347995

[18] Kim MN, Yong D, An D, Chung HS, Woo JH, et al (2012) Nosocomial clustering of NDM-1-producing *Klebsiella pneumoniae* sequence type 340 strains in four patients at a South Korean tertiary care hospital. J Clin Microbiol 50: 1433–1436. 22259206 10.1128/JCM.06855-11 PMC3318568

[19] Huang TW, Wang JT, Lauderdale TL, Liao TL, Lai JF, et al (2013) Complete sequences of two plasmids in a *bla* _NDM-1_-positive *Klebsiella oxytoca* isolate from Taiwan. Antimicrob Agents Chemother 57: 4072–4076. 23752513 10.1128/AAC.02266-12 PMC3719732

[20] Toleman MA, Bennett PM, Walsh TR (2006) IS*CR* elements: novel gene-capturing systems of the 21st century? Microbiol Mol Biol Rev 70: 296–316. 16760305 10.1128/MMBR.00048-05 PMC1489542

[21] Chen YT, Liao TL, Liu YM, Lauderdale TL, Yan JJ, et al (2009) Mobilization of *qnrB2* and IS*CR1* in plasmids. Antimicrob Agents Chemother 53: 1235–1237. 19075060 10.1128/AAC.00970-08 PMC2650544

[22] Li J, Lan R, Xiong Y, Ye C, Yuan M, et al (2014) Sequential isolation in a patient of *Raoultella planticola* and *Escherichia coli* bearing a novel IS*CR1* element carrying *bla* _NDM-1_ . PLoS One 9: e89893. 24594606 10.1371/journal.pone.0089893 PMC3940617

