# Supplementary material for: Closely Related NDM-1-Encoding Plasmids from Escherichia coli and Klebsiella pneumoniae in Taiwan
Source: PLoS One. 2014 Aug 21;9(8):e104899. doi: 10.1371/journal.pone.0104899 (PMC4140731; doi:10.1371/journal.pone.0104899)
Supplement: Sequence S1 — Complete nucleotide sequences and annotations of plasmids pLK75. (DOCX) [file pone.0104899.s001.docx]

LOCUS pLK75 56489 bp DNA circular BCT 09-FEB-2014

DEFINITION Escherichia coli plasmid pLK75, whole genome shotgun sequence.

ACCESSION

VERSION

KEYWORDS WGS.

SOURCE Escherichia coli

ORGANISM Escherichia coli

Bacteria; Proteobacteria; Gammaproteobacteria; Enterobacteriales;

Enterobacteriaceae; Escherichia.

REFERENCE 1 (bases 1 to 56489)

AUTHORS Chen,Y.-T., Lin,A.-C. and Siu,K.

TITLE Sequence of closely related NDM-1-encoding plasmids from

Escherichia coli and Klebsiella pneumoniae in Taiwan

JOURNAL Unpublished

REFERENCE 2 (bases 1 to 56489)

AUTHORS Chen,Y.-T., Lin,A.-C. and Siu,K.

TITLE Direct Submission

JOURNAL Submitted (09-FEB-2014) Institute of Molecular and Genomic

Medicine, National Health Research Institutes, 35, Keyan Road,

Zhunan Township, Miaoli County, Taiwan 350, Taiwan

COMMENT ##Assembly-Data-START##

Assembly Method :: Newbler v. 2.7

Sequencing Technology :: 454; Illumina

##Assembly-Data-END##

FEATURES Location/Qualifiers

source 1..56489

/organism="Escherichia coli"

/mol_type="genomic DNA"

/db_xref="taxon:562"

/plasmid="pLK75"

/country="Taiwan"

CDS 1..720

/codon_start=1

/transl_table=11

/product="repA"

/translation="MDKLLNKKIKVKQSNELTEAAYYLSLKAKRVLWLCLMQTYFTAS

VSEDDDEMAVLGDSTFKVKVADYEQIFQVSRNQAIKDVKEGVFELSRSAVIFYPKEGS

FDCVARPWLTEAGSRSARGIWEIEFNHKLLRYIYGLTNQFTTYSLRDCGSLRNPRTIR

LYESLAQFKSSGLWVTTHAWLNDRFLLPESQQKNLAELKRSFLDPALKQINEKTPLLA

KYSIDDSGKFLFSIIDKQNPV"

misc_feature 2136..2160

/note="IRi"

CDS complement(2338..3351)

/codon_start=1

/transl_table=11

/product="integron integrase IntIPac"

/translation="MKTATAPLPPLRSVKVLDQLRERIRYLHYSLRTEQAYVHWVRAF

IRFHGVRHPATLGSSEVEAFLSWLANERKVSVSTHRQALAALLFFYGKVLCTDLPWLQ

EIGRPRPSRRLPVVLTPDEVVRILGFLEGEHRLFAQLLYGTGMRISEGLQLRVKDLDF

DHGTIIVREGKGSKDRALMLPESLAPSLREQLSRARAWWLKDQAEGRSGVALPDALER

KYPRAGHSWPWFWVFAQHTHSTDPRSGVVRRHHMYDQTFQRAFKRAVEQAGITKPATP

HTLRHSFATALLRSGYDIRTVQDLLGHSDVSTTMIYTHVLKVGGAGVRSPLDALPPLT

SER"

misc_feature 3432..3492

/note="attI"

CDS 4819..5352

/codon_start=1

/transl_table=11

/product="dihydrofolate reductase"

/translation="MRNRRATLSPFMAALTQRSIVKISLMAAKARNGVIGCGSDIPWN

AKGEQLLFKAITYNQWLLVGRKTFEAMGALPNRKYAVVSRSGSVATNDDVVVFPSIEA

AMRELKTLTNHVVVSGGGEIYKSLIAHADTLHISTIDSEPEGNVFFPEIPKEFNVVFE

QEFHSNINYRYQIWQRG"

CDS 5533..6378

/note="Streptomycin 3''-O-adenylyltransferase"

/codon_start=1

/transl_table=11

/product="aadA1"

/translation="MSNAVPAEISVQLSQALNVIEHHLGSTLLAVHLYGSALDGGLKP

CSDIDLLVTVTAQLDETVRQALFVDFLEVSASPGQSEALRALEVTIVVYGDVVPWRYP

ARRELQFGEWQRKDILAGIFEPATTDVDLAILLTKARQHSLALAGSAAEDFFNSVPES

DLFKALADTLKLWNSQPDWAGDERNVVLTLSRIWYSAATGKIAPKDVAANWVMERLPV

QHQPVLLEAQQAYLGQGMDCLASRADQLTAFIYFVKHEAASLLGSTPMMSNSSFKPTP

LRGAA"

CDS 6836..7675

/codon_start=1

/transl_table=11

/product="dihydropteroate synthase"

/translation="MVTVFGILNLTEDSFFDESRRLDPAGAVTAAIEMLRVGSDVVDV

GPAASHPDARPVSPADEIRRIAPLLDALSDQMHRVSIDSFQPETQRYALKRGVGYLND

IQGFPDPALYPDIAEADCRLVVMHSAQRDGIATRTGHLRPEDALDEIVRFFEARVSAL

RRSGVAADRLILDPGMGFFLSPAPETSLHVLSNLQKLKSALGLPLLVSVSRKSFLGAT

VGLPVKDLGPASLAAELHAIGNGADYVRTHAPGDLRSAITFSETLAKFRSRDARDRGL

DHA"

CDS complement(8912..9445)

/codon_start=1

/transl_table=11

/product="nuc"

/translation="MKNLATWLLAAAFTTAALPAFAVEPSVQVGYSPEGSARVLVLSA

IDSAKTSIRMMAYSFTAPDIMKALVAAKKRGVDVKIVIDERGNTGRASIAAMNYIANS

GIPLRTDSDFPIQHDKVIIVDNVTVETGSFNFTKAAETKNSENAVVIWNMPKLAESFL

EHWQDRWNRGRDYRSSY"

CDS complement(9445..10440)

/codon_start=1

/transl_table=11

/product="traG"

/translation="MTDAAFYQLGPLREYLEDPTVFEIRINCFQEVICDTFSGRRVVQ

NAAITADFIRNLAKSLVSSNKLTMQAINDVILPGGIRGVICLPPAVIDGTTAVAFRKD

LAADKNLEQLTSEGIFSDCRKITGSKQSLTDDDFFLKELHSSEKWPAFLQTAVEKKRT

IVICGETGSGKTVLTRALLKSLHKDERVIILEDVHEVTVDHVVEAVYMMYGDAGKIGR

VSATDALRACMRLTPGRIIMTELRDDAAWDYLKALNTGHPGGVMSTHANSARDAFNRI

GLLIKATPIGRMLDMSDIMRMLYSTIDVVVHMEKRKIKEIYFDPEYKMQCVNGSL"

CDS complement(10482..11642)

/codon_start=1

/transl_table=11

/product="traF"

/translation="MARKSVDVDQELDENTGDGEFESERGGFKGSNRRSAPGMKAFVI

LMALLALVFIGITVMGKIRTPAKAEADKDGGKAQQANTLPNYSFNSDPDVNKPATAQN

SATDARAVQAAAQADADAGSSNTAARTSNKRKEPSPEELAMQRRLGGELAQTNQAATS

NSPGVQPQDNETSEGSSALAKNLTPARLKASRAGVMANPSLTVPKGKMIPCGTGTELD

TTVPGQVSCRVSQDVYSADGLVRLIDKGSWVDGQITGGIKDGQARVFVLWERIRNDQD

GTIVNIDSAGTNSLGSAGIPGQVDTHMWERLRGAIMISLFSDTLTALVNQTQSNNIQY

NSTENSGEQLASEALRSYMSIPPTLYDQQGDAVSIFVARDLDFSGVYTLADN"

CDS complement(11642..12526)

/codon_start=1

/transl_table=11

/product="traO"

/translation="MKKLLLSAVVLSVLGGAATNVMALEVGRNSPYDYRIKSVVYNPV

NVVKIDAIAGVATHIVVAPDETYITHAFGDSESWTFAHKMNHFFVKPKQAMSDTNLVI

VTDKRTYNIVLHFIGEETKKNADGTVSKSFIETPWAVRQAVLQLTYEYPFEQQEKAKS

AADKKRITQKLKQTAFAGAKNYQYVMSEQPEMRSIQPVHVWDNYRFTRFEFPANAELP

QVYMISASGKETLPNSHVVGENRNIIEVETVAKEWRIRLGDKVVGVRNNNFAPGAGAV

ATGTASPDVRRVQIGEDN"

CDS complement(12537..13235)

/codon_start=1

/transl_table=11

/product="traE"

/translation="MKANKKTGLTREAIKEFNESRKGLEVDLMDEVLKSRRTAWMVAT

GSAVVTVFALSLVGYVVHKYSQPIPAHLLTLNEATHEVQQVKLTRDQTSYGDEIDKFW

LTQYVIHRESYDFYSVQVDYTAVGLMSTPNVAESYQSKFKGRNGLDKVLGDSETTRVK

INSVILDKPHGVATIRFTTVRRVRSNPVDDQPQRWIAIMGYEYKSLAMNAEQRYVNPL

GFRVTSYRVNPEVN"

CDS complement(13454..14437)

/codon_start=1

/transl_table=11

/product="traD"

/translation="MVSANVATIISDVTPLIATCLTIKLMVQGMYSAFNPGAGDSLSS

LIKEYLSIALILSFATAGGWYQQELVNVALHLPDDFAGILSAPNKVGASGVPAIIDSG

IEKGIKIVNTAWEAADVFSSSGLAAYAIGGIMMIATVVLGGLGAGFVIMAKILLAVTL

CFGPIAIFCLLWGATKNIFARWLASVINYGLVVVILALVFGFIMQMFDNLLSSMNSDA

AYSSITGSISALLLTVISVFVLFQIPQIAASWGSGISAGVADAARSTGSSMQALGNMG

SHGMFGGNAFRGGNSGGGQQSAGGGSGSNSGGSSGSNLSGKARGSRGKKAA"

CDS complement(14510..14737)

/codon_start=1

/transl_table=11

/product="eex"

/translation="MKKLLLVIPFLLVACDASHDVEWYKKHEKERKATIQECKKDADE

LQKPDCKNAREADRQLFVFGKKDGEINSPKI"

CDS complement(14745..15458)

/codon_start=1

/transl_table=11

/product="traC"

/translation="MKKTLTAVLLTTGLILGGAQSASAGIIVTNPTELAKQVEQLQQM

AQQLEQLKSQLQTQKNMYESMAKTTNLGDLLGTSTSTLANNLPDNWKEIYSDAMNSSS

SVTPSVNSMMGQFNAEVDDMTPSEAITYMNKKLAEKGAYDRVMAEKAYNNQMQELTDM

QELTEQIKTTPDLKSIADLQARIQTSQGAIQGEQAKLNLMNMLQQSQDKLLRAQKERA

THNFVFGTGGDVTASPSIN"

CDS complement(15476..18076)

/codon_start=1

/transl_table=11

/product="traB"

/translation="MRAATATKPKKIDAYRKEPSVNKKYLPYSYHLNDYVISMENGDL

MAFFKLDGRTHDCASDRELVTWHKDLNTLVKSFGTDHVELWTHEYHHEAKEYPDGEYD

HFFPAYVDQYNRKLHGDSKQLINDLYLTVIYKQVGDKTQKFLAKFEKPTRDEIQQMQN

EALEGLEDISEQILEAMKPYGIQQLGIYYRDKRGVEIPAPDKKEREELAEVDESDIFD

EAIVIERNEPEPSQAHAYSKALEFLYFLANMEWAIVPVCRDRIREYIMDNRPVSSLWG

DVVQIRTVDHNFYTTGIEFREYEEDTEPGQLNMLKEADFEYLLTQSFSCLSESSAKTF

LTHQEKSLQETRDRAQSQLAQLGTALDMLTSREFVMGYHHGTVHVWDNDQNAVQRKAR

RVKVMLTGCGVVGGTISLASEAAYYARLPGNQKWAPRPVPINSWNFLHFSPFHNFMRG

KPDNNPWGPALTMFRTISGTPLYFNFHVTPLEELSYGKRPLGHALITGMSGEGKTTLL

NFLLAQSMKYNPRLFVYDRDRGMEPFIRSVGGYYKVLQQGMPSGFAPLQIEPTKRNIA

LIKNLFRICVETTNNGPISATMATELAEGVDAVMGEGSLIPREARTVTILDGYVNEVV

ENGVSLKGLLREWTREGQYGWLFDNDKDSLDLSANDIFGFDLSEFIAAKEEVSSPART

PLMMYLLYRVRDSIDGKRRVIQCFDEFHAYLDDPVIEREVKRGIKTDRKKDAIYVFAT

QEPNDALSSRIGRTIMSQTVTKICLRDPEAIREDYAFLTDAEYDALMSITEHSRQFLV

KQGQQSAIASFNLYPRNSDDIDADIKTMDNVLSVLSGEPQNAEIAHELVERLGNDPEV

WLKEYWRLTA"

misc_feature complement(15912..15936)

/note="IRt"

CDS complement(18076..18393)

/codon_start=1

/transl_table=11

/product="traA"

/translation="MFVDGKRPLFKGATRLPRALGVPRNVAMMIFMISASLFMIIHMW

AILVFVFLWIPSAALTKYDDRMFRIMGLWLKTKFSNWFDSPFKQWGGSSYSSVDYKRK

GLK"

CDS complement(18443..18736)

/codon_start=1

/transl_table=11

/product="traM"

/translation="MTTLFKKYGPAVVMGVLSIALPQIALAAGTDTGESTATSIQTWL

STWIPIGCAIAIMVSCFMWMLHVIPASFIPRIVISLIGIGSASFLVSLTGVGS"

CDS complement(18746..19039)

/codon_start=1

/transl_table=11

/product="korA"

/translation="MRLWMERLYLVLQSQILELFEAGKVKEVTIERVSLKKWYPVFQI

DDEQLGQIACSIRVNKEHELRTWADLRLLAEFLKDKCGVEECRLNLQSTEDSE"

CDS complement(19036..19770)

/codon_start=1

/transl_table=11

/product="traL"

/translation="MSKHPKLLVLALACLACAGRASAAPASDEVARLAQRCAPDVSPL

TMAYIVGHESSNGPYRININGSIQLKQQPRTEAEAVSVAKVLLKDNKSFDMGLAQINS

NNLVGLGLSVDDIFKPCINLRASQTILKACYDSALKSYPAGQVALRHALSCYNTGSLT

NGISNGYVTKVINVARQSTDLKIPTLLPDGQTSEDSTATEPQQAKSTAPQYDGEQDVF

GSGDGDAFSRNNTDAFLTRQETAKGE"

CDS 19879..20184

/codon_start=1

/transl_table=11

/product="korB"

/translation="MLVPLKSEKRPKGEPVYRDPDNPFNTWTGIGKRPAWLTAKLDAG

ISLEAMKMQGVANPREHRPAKYRDPRNAENTWSGTGRRPTWLKELLDSGLSLDDLKI"

CDS 20200..20544

/codon_start=1

/transl_table=11

/product="hypothetical protein"

/translation="MGELIDFAERQKSRRKKKASSIPPIFRKFRVHAIRLLASIIKSG

SYSVAYIVKKITGKLIKFYTILTIFVFVVEYIAGDIGYKSIYNAALLLILLTVINILA

SVYLNKLLRTKQ"

CDS 20541..20855

/codon_start=1

/transl_table=11

/product="kikA"

/translation="MKKLLIPLIAAGSLLYIPASHAEDPCKVIMCMAGKLTGDSGGSE

CNSAEAAFFNIVKKNKHGFLPNHTKDARKAFLNECPDNGEGGSNQSMISQIISKYGKV

RL"

CDS 21108..21899

/codon_start=1

/transl_table=11

/product="mrr"

/translation="MAPATRPAPYFTGGFARISQAALRGRYTAVAKHQGGLTALFFTL

RKPTMTMIPFPTTENLILWACSAIALLAVVFFRRSVRHRRHKRKQQSARRVLERIKTL

PGFPQKINYLRKIDPFVFEELLLEGFEAHGFRTIRNKRYTGDGGIDGQVIIGKYRYLI

QAKRYRGHIALQHVQEFEKLLKRHNCRGLFCHTGKTGAGSKSVSIASERMEIISGQRL

IDLLTPGSSFTIATAPQTMMKRTAATLETSTIVKDAGKENRYHES"

CDS 22492..23925

/codon_start=1

/transl_table=11

/product="EcoRIIM"

/translation="MSEFELLAQDLLEKAEAEEQLRQENDKKLLGQVLEIYDQKYVAE

LLRKVGKNEWSRETLNRWINGKCSPKTLTLAEEELLRKMLPEAPAHHPDYAFRFIDLF

AGIGGIRKGFETIGGQCVFTSEWNKEAVRTYKANWFNDAQEHTFNLDIREVTLSDKPE

VPENDAYAYINEHVPDHDVLLAGFPCQPFSLAGVSKKNSLGRAHGFECEAQGTLFFDV

ARIIRAKKPAIFVLENVKNLKSHDKGKTFKVIMDTLDELGYEVADAAEMGKNDPKVID

GKHFLPQHRERIVLVGFRRDLNIHQGFTLRDISRFYPEQRPSFGELLEPVVDSKYILT

PKLWEYLYNYAKKHAAKGNGFGFGLVNPENKESIARTLSARYHKDGSEILIDRGWDMA

TGETDFANEENQAHRPRRLTPRECARLMGFEKVDGRPFRIPVSDTQSYRQFGNSVVVP

VFEAVAKLLEPYILKAVNADSCKVERI"

CDS complement(23959..25167)

/codon_start=1

/transl_table=11

/product="EcoRII"

/translation="MSVFHNWLLEIACENYFVYIKRLSANDTGATGGHQVGLYIPSGI

VEKLFPSINHTRELNPSVFLTAHVSSHDCPDSEARAIYYNSRHFGKTRNEKRITRWGR

GSPLQDPENTGALTLLAFKLDEQGGDCKEVNIWVCASTDEEDVIETAIGEVIPGALIS

GPAGQILGGLSLQQAPVNHKYILPEDWHLRFPSGSEIIQYAASHYVKNSLDPDEQLLD

RRRVEYDIFLLVEELHVLDIIRKGFGSVDEFIALANSVSNRRKSRAGKSLELHLEHLF

IEHGLRHFATQAITEGNKKPDFLFPSAGAYHDTEFPVENLRMLAVKTTCKDRWRQILN

EADKIHQVHLFTLQEGVSLAQYREMRESGVRLVVPSSLHKKYPEAVRAELMTLGAFIA

ELTGLYADIP"

misc_feature complement(25228..25252)

/note="IRt"

mobile_element 25380..25393

/rpt_type=inverted

/mobile_element_type="insertion sequence:IS6100"

CDS complement(25434..26198)

/codon_start=1

/transl_table=11

/product="transposase of IS6100"

/translation="MTDFKWRHFQGDVILWAVRWYCRYPISYRDLEEMLAERGISVDH

TTIYRWVQCYAPEMEKRLRWFWRRGFDPSWRLDETYVKVRGKWTYLYRAVDKRGDTID

FYLSPTRSAKAAKRFLGKALRGLKHWEKPATLNTDKAPSYGAAITELKREGKLDRETA

HRQVKYLNNVIEADHGKLKILIKPVRGFKSIPTAYATIKGFEVMRALRKGQARPWCLQ

PGIRGEVRLVERAFGIGPSALTEAMGMLNHHFAAAA"

mobile_element complement(26246..26259)

/rpt_type=inverted

/mobile_element_type="insertion sequence:IS6100"

CDS complement(27333..28172)

/codon_start=1

/transl_table=11

/product="dihydropteroate synthase"

/translation="MVTVFGILNLTEDSFFDESRRLDPAGAVTAAIEMLRVGSDVVDV

GPAASHPDARPVSPADEIRRIAPLLDALSDQMHRVSIDSFQPETQRYALKRGVGYLND

IQGFPDPALYPDIAEADCRLVVMHSAQRDGIATRTGHLRPEDALDEIVRFFEARVSAL

RRSGVAADRLILDPGMGFFLSPAPETSLHVLSNLQKLKSALGLPLLVSVSRKSFLGAT

VGLPVKDLGPASLAAELHAIGNGADYVRTHAPGDLRSAITFSETLAKFRSRDARDRGL

DHA"

CDS complement(28637..29452)

/note="Streptomycin 3''-O-adenylyltransferase"

/codon_start=1

/transl_table=11

/product="aadA1"

/translation="MAKTKLNIMREVVIAEVSTQLSEVVGVIERHLEPTLLAVHLYGS

AVDGGLKPHSDIDLLVTVTVRLDETTRRALINDLLETSASPGESEILRAVEVTIVVHD

DIIPWRYPAKRELQFGEWQRNDILAGIFEPATIDIDLAILLTKAREHSVALVGPAAEE

LFDPVPEQDLFEALNETLTLWNSPPDWAGDERNVVLTLSRIWYSAVTGKIAPKDVAAD

WAMERLPAQYQPVILEARQAYLGQEEDRLASRADQLEEFVHYVKGEITKVVGK"

CDS complement(29445..29951)

/codon_start=1

/transl_table=11

/product="putative beta-lactamase"

/translation="MKQWERDLTLRGAIQVSAVPVFQQIAREVGEVRMQKYLKKFSYG

NQNISGGIDKFWLEGQLRISAVNQVEFLESLYLNKLSASKENQLIVKEALVTEAAPEY

LVHSKTGFSGVGTESNPGVAWWVGWVEKETEVYFFAFNMDIDNESKLPLRKSIPTKIM

ESEGIIGG"

mobile_element 30242..30258

/rpt_type=inverted

/mobile_element_type="insertion sequence:ISAba125"

CDS 30362..31174

/note="New Delhi metallo-beta-lactamase 1"

/codon_start=1

/transl_table=11

/product="beta-lactamase NDM-1"

/translation="MELPNIMHPVAKLSTALAAALMLSGCMPGEIRPTIGQQMETGDQ

RFGDLVFRQLAPNVWQHTSYLDMPGFGAVASNGLIVRDGGRVLVVDTAWTDDQTAQIL

NWIKQEINLPVALAVVTHAHQDKMGGMDALHAAGIATYANALSNQLAPQEGMVAAQHS

LTFAANGWVEPATAPNFGPLKVFYPGPGHTSDNITVGIDGTDIAFGGCLIKDSKAKSL

GNLGDADTEHYAASARAFGAAFPKASMIVMSHSAPDSRAAITHTARMADKLR"

CDS 31283..31543

/codon_start=1

/transl_table=11

/product="bleomycin resistnace protein"

/translation="MILQRGGLQLEFFPYPDLDPATSSFGCCLRLDDLDAMVALVNAA

GAEEKSTGWPRFKAPQLEASGLRIGYLIDPDCTLVRLIQNPD"

CDS 31548..32186

/codon_start=1

/transl_table=11

/product="phosphoribosylanthranilate isomerase"

/translation="MPAKIKICGISTPEALDATIAARADYAGLVFYPASPRAVTSNVA

GALTSRAAGQIAMVGLFVDADDAVIADALVAAKLNALQLHGSESPERVAQLRARFGKP

VWKALPVASASDVARAAAYAGAADLILFDAKTPKGALPGGMGLAFDWSLLAGYRGALP

WGLAGGLNPTNVAEAIARTGAPLVDTSSGVESAPGVKDTDKITNFAFAVRLA"

CDS complement(32197..32409)

/codon_start=1

/transl_table=11

/product="putative alpha-N-acetylgalactosaminidase"

/translation="MDWFVIHAFVEALKAKAPMPIDIYDALAWSAITPLSEQSIAEGN

RTLDFPDFTRGQWRTRKPIFALNDAY"

misc_feature complement(32552..32573)

/note="oriIS, replication origin of ISCR"

CDS complement(32784..34325)

/codon_start=1

/transl_table=11

/product="ISCR1 (orf513)"

/translation="MSLARNATASQSPTQTNGYERHQPDQTLLYQLVEQHYPAFKASL

EAQGQHLPRYIQQEFNDLLQCGRLEYGFMRVRCEDCHHERLVAFSCKRRGFCPSCGAR

RMAESAALLIDEVFPKEPIRQWVLSFPFQLRFLLARHPQLMGQVLSIVYRTLSTHLIK

KAGYTKASAQTGSVTLIQRFGSALNLNVHYHMLFLDGVYAEDDYGKQRFHRVKAPTYD

ELNTLAHTLSHRIARCMEKRGILERDAENTWLTLEEGEDDTLTQLHGASVTYRIAVGP

QQGRKVFTLQTLPGREDKADSSSRVANHAGFSLHAGVMAEAHQRDKLERLCRYISRPA

VSEKRLALTANGQVRYELKTPYRNGTTHVIFEPLDFIAKLAALVPKPRVNLTRFHGVF

APNSKHRVQVTPAKRGKKPDKSEGLDTNWRDKSPAERHRAMTWMQRLKRVFNIDIEVC

EHCGGHVKVIASIEDPKVIEQILKHLKQKTAKANAAKQRELPPERAPPLTPSLFDPSQ

SRLFD"

CDS 34679..34921

/codon_start=1

/transl_table=11

/product="relaxase/helicase"

/translation="MKTAALDLARERQAHEAGARTRATAHERTPQQERQKAAREAERG

REAWTLGQGMKKPVAGCYGRLTRWKGGGDVVYMALL"

CDS complement(34953..35630)

/note="putative transcriptional regulator, tetR family"

/codon_start=1

/transl_table=11

/product="putative transcriptional regulator"

/translation="MFISDKVSSMTKLQPNTVIRAALDLLNEVGVDGLTTRKLAERLG

VQQPALYWHFRNKRALLDALAEAMLAENHTHSVPRADDDWRSFLIGNARSFRQALLAY

RDGARIHAGTRPGAPQMETADAQLRFLCEAGFSAGDAVNALMTISYFTVGAVLEEQAG

DSDAGERGGTVEQAPLSPLLRAAIDAFDEAGPDAAFEQGLAVIVDGLAKRRLVVRNVE

GPRKGDD"

CDS 35709..36908

/codon_start=1

/transl_table=11

/product="tetracycline efflux protein TetA"

/translation="MKPNRPLIVILSTVALDAVGIGLIMPVLPGLLRDLVHSNDVTAH

YGILLALYALVQFACAPVLGALSDRFGRRPILLVSLAGATVDYAIMATVPFLWVLYIG

RIVAGITGATGAVAGAYIADITDGDERARHFGFMSACFGFGMVAGPVLGGLMGGFSPH

APFFAAAALNGLNFLTGCFLLPESHKGERRPLRREALNPLASFRWARGMTVVAALMAV

FFIMQLVGQVPAALWVIFGEDRFHWDATTIGISLAAFGILHSLAQAMITAPVAARLGE

RRALMLGMIADGTGYILLAFATRGWMAFPIMVLLASGGIGMPALQAMLSRQVDEERQG

QLQGSLAALTSLTSIVGPLLFTAIYAASITTWNGWAWIAGAALYLLCLPALRRGLWSG

AGQRADR"

CDS complement(36940..37638)

/note="permease of the drug/metabolite transporter (DMT)

superfamily"

/codon_start=1

/transl_table=11

/product="putative permease"

/translation="MAWPAAVLPCFISRFLYRPLSVYRLPGGVAATVGAVQPLMVVFI

SAALLGSPIRLMAVLGAICGTAGVALLVLTPNAALDPVGVAAGLAGAVSMAFGTVLTR

KWQPPVPLLTFTAWQLAAGGLLLVPVALVFDPPIPMPTGTNVLGLAWLGLIGAGLTYF

LWFRGISRLEPTVVSLLGFLSPGTAVLLGWLFLDQTLSALQIIGVLLVIGSIWLGQRS

NRTPRARIACRKSP"

CDS complement(37788..37961)

/codon_start=1

/transl_table=11

/product="hypothetical protein"

/translation="MEQTDKRKQDKLKFDRVINLARRLPQPAIHDLLRALILPIQADY

LLGSSQNSENKAR"

CDS complement(37979..41197)

/codon_start=1

/transl_table=11

/product="traI"

/translation="MLDITTITRQNVTSVVGYYSDAKDDYYSKDSSFTSWQGTGAEAL

GLSGDVESARFKELLVGEIDTFTHMQRHVGDAKKERLGYDLTFSAPKGVSMQALIHGD

KTIIEAHEKAVAAAVREAEKLAQARTTRQGKSVTQNTNNLVVATFRHETSRALDPDLH

THAFVMNMTQREDGQWRALKNDELMRNKMHLGDVYKQELALELTKAGYELRYNSKNNT

FDMAHFSDEQIRAFSRRSEQIEKGLAAMGLTRETADAQTKSRVSMATREKKTEHSREE

IHQEWASRAKTLGIDFDNREWQGHGKPLEADIARNMAPDFTSPEVKADRAIQFAVKSL

SERDASFERQKLIQIANKQVLGHATIADVEKAYLKAVQKGAIIEGEARYQSTLKVGAS

VMAETLTRKEWIDSLTNSGMRADKARFAVDDGIKNGRLKKTSHRVTTVEGIRLERSIL

TIESRGRGQMPRQLTAEIAGQLLAGKTLKKEQMRAVTEIVTSKDRFVAAHGYAGTGKS

YMTMAAKELLESQGLKVTALAPYGTQKKALEDDGLPARTVAAFLKAKDKKLDEKSVVF

IDEAGVIPARQMKQLMEVIEKHNARAVFLGDTSQTKAVEAGKPFEQLIKAGMQTSYMK

DIQRQKNEVLLEAVKYAAEGNAARALKNITGVNELKEEAPRLSQLADRYLSLSSEQQD

ATLIISGTNASRKTLNDYIRGNLGLAGTGETFTLLDRVDSTQAERRDSRYFSKGQIII

PEQDYKNGMKRGESYQVLDTGPGNKLTVESSSGEQIAFSPRTHTKLSVYQAVSAELAP

GDKVMVTRNDKTLDVANGDRFTVKTVEGEKLTLEDKKGRTVELDKKQASYLSYAYATT

VHKSQGLTCDRVLFNIDTKSLTTSKDVFYVGISRARHEVEIFTDDKKSLASSVSRDSP

KTTAAEIDRFFGLEARFKDIGRDTSLETRSAEKGLPEATGESMAFNQKPDEHNMTTGT

DYQPVSNAEDAFHLKQNPMDDSVGLRRHEAQQNDAELAHDYAAADDQQWSAQEYADYE

HYAEASDYDFDSSIYDDYAMPQTSQAEQSHTGKEHTHEHEHE"

CDS complement(41197..42726)

/codon_start=1

/transl_table=11

/product="traJ"

/translation="MDDRERGLAFLFAITLPPVMVWFLVAKFTYGIDPSTAKYLIPYL

VKNTFSLWPLWSALIAGWFIGVGGLIAFIIYDKSRVFKGERFKKIYRGTELVSARTLA

DKTRERGVNQLTVANIPIPTYAENLHFSIAGTTGTGKTTIFNELLFKSIIRGGKNIAL

DPNGGFLKNFYRPGDVILNAYDKRTEGWVFFNEIRRSYDYERLVNSIVQESPDMATEE

WFGYGRLIFSEVSKKLHSLYSTVTMEEVIHWACNVDQKKLKEFLMGTPAEAIFSGSEK

AVGSARFVLSKNLAPHLKMPEGNFSLRDWLDDGKPGTLFITWQEEMKRSLNPLISCWL

DSIFSIVLGMGEKESRINVFIDELESLQFLPNLNDALTKGRKSGLCVYAGYQTYSQLV

KVYGRDMAQTILANMRSNIVLGGSRLGDETLDQMSRSLGEIEGEVERKESDPQKPWIV

RKRRDVKVVRAVTPTEISMLPNLTGYLALPGDMPVAKFKAKHVKYHRKNPVPGIELRD

I"

CDS complement(42728..43144)

/codon_start=1

/transl_table=11

/product="traK"

/translation="MPIITAKVSDELLAYIDLVSGGNRSDYLRRCIEAGPGDRESGLK

IVADRLSDVNRKLDYLFDRASDADFGPLRDELKAITETLSGVKFPPAGQMMLHESLAI

ETLILLRSIAEPGKTKAAKAEVERNGYKVWEPKKER"

misc_feature complement(43463..45182)

/note="oriT region"

CDS 43635..44054

/codon_start=1

/transl_table=11

/product="stbA"

/translation="MKPKSIRAALQLMLPEIEEMLSLGVSREEIYKAVSERFGLEGVN

VRSFDTSLYRARQIRKNGMHNTHERMPNNDDSVLHNTQKGGSEKGAEESVLHNTQTPP

EPEPQGSEKKESPGIIDKEFFNKISEDFDPKMFNKKF"

CDS 44063..44779

/codon_start=1

/transl_table=11

/product="hypothetical protein"

/translation="MKVAVINYSGSVGKTLISSYLLAPRLTGAKFYAVETINQSASDL

GIENVSIFKGDDFSRLIEDIVFEDAGIIDIGASNVEAFLMAMSRFDSGANEFDKYVIP

VTPDNKAIDESLKTAHTLSKAGVSSDKIIFVPNRISPDSEVEDVLAPVFEFVKRTKVG

KISKKSVIYNSEVFEYLAYHRISFEALTAEDPEEFKARAKQTTDADERKKLARRYTYM

KQAIPVKANLDKAYAALMGE"

CDS 44781..45149

/codon_start=1

/transl_table=11

/product="stbC"

/translation="MEKQPDKFEVLMDWFLGDAKEITASQKEMTEILSALSEKLAKDT

ESLGETADSLKRTLVENQRSISLAISDDAKAREEFLTKFRRAQVSRAETLTRQILFIT

AGCTIVGAAVGAAIAIILLR"

CDS 45331..45675

/codon_start=1

/transl_table=11

/product="orfD"

/translation="MNDRQREQARIRQARRRARLKEEGASVTVTLTKQEEAMLQELCR

VRRPGRTAYSTNEFFQLLLIRNWQQWQEQKAQLGKCQACGKLKAEGGCGGERQSETFN

CWLAVEANELNV"

CDS complement(45786..46106)

/codon_start=1

/transl_table=11

/product="CcgAII protein"

/translation="MTTQTLEQTLEDFRRQCESFAREQQPRCGLIYELYQRRLSAVID

GYLAGVPAEYREELIAVARREFDYLTQDEIAEEIRQDRENDYCSHGIERNCCPLGCGD

LDDY"

CDS complement(46161..46340)

/codon_start=1

/transl_table=11

/product="CcgAI"

/translation="MTTVTTPSQLKKEVESQKELLLRACLEAFNQLPNQRLQGAFTST

YALAAKLDQLLQQTK"

CDS 46936..47049

/codon_start=1

/transl_table=11

/product="hypothetical protein"

/translation="MASGFSAMAEHELERSPASSSKGAAQPPRWLFADTGD"

CDS complement(47094..47603)

/codon_start=1

/transl_table=11

/product="Antirestriction protein ArdA"

/translation="MTDITTPSVYVGTYHKYNCGSIAGAWLDLTDFDSSEEFYERCRE

LHANEADPEFMFQDWEGIPSDMASECHINWDFINGFKQAREEGNEAAFVAFVDLFNST

DFDLFRDAYMGEAKDEETFAEEYLNDSGLLNEIPESVARYFDIVAYARDLFIGDFSLH

DGHVFNMTC"

CDS complement(48956..49288)

/codon_start=1

/transl_table=11

/product="hypothetical protein"

/translation="MDYQTRLNSDITKEIDYLASLRKQRMVADLRTELVYGSLERLAD

MICNTVTDWSLPCPVLPLSSVQQWHKAREIVLADYEDFGHDAWDFARHYMKTELSFGY

ACYKDDIA"

CDS complement(49497..49781)

/codon_start=1

/transl_table=11

/product="hypothetical protein"

/translation="MYHYLVFVPLALIFSAAALALPWFIVRAGYRDLVQITCACVSVL

GLCAATGVFSYFDNKPLAFLYGCGALACFLYAVDCAIPLYMTRKKPRKQL"

CDS complement(49850..50050)

/codon_start=1

/transl_table=11

/product="hypothetical protein"

/translation="MAGVIVHPVDRPAAKVAWFSERICGKKRYPVSFISDEISAPHNV

IHKGEKFHVTPAQVNLLDAQNL"

CDS complement(50206..50502)

/codon_start=1

/transl_table=11

/product="hypothetical protein"

/translation="MHFTNFLQRYFDIEIEHTFDPTIQGSNETGKDVTKIWIYEKGED

SEPLLTLTEAWWYTETKTAGNWLIGNVYSTLEHGREIHESEFRKLVTAGKVISA"

CDS complement(51637..51972)

/codon_start=1

/transl_table=11

/product="hypothetical protein"

/translation="MFDYRNSDQERYGQQIYHHYRKQGNHRWDTSVHQDSGGQYAIIF

RHSFSKKQADGVKRTMIRDETVIRAGTAQELTEATFPDFQDSDILKASDFFKSLIQRK

AADVTQTDI"

CDS complement(52099..52524)

/codon_start=1

/transl_table=11

/product="antirestriction protein KlcA"

/translation="METIEITARYISENARMNFMPAAFRGAFFSADHFIQSFLNRYAK

DYQGGYWEYLQASNGAFFMEAPQPLWLSLPNYFEGECSAREVGIIVCLYAYSYFCGLA

YEEGKAELNETMANRYHLLREYVNTLENESQNRIYRAID"

CDS 52939..53379

/codon_start=1

/transl_table=11

/product="error-prone repair protein UmuD"

/translation="MKVDIFESSGASRVHSIPFYLQRISAGFPSPAQGYEKQELNLHE

YCVRHPSATYFLRVSGSSMEDGRIHDGDVLVVDRSLTASHGSIVVACIHNEFTVKRLL

LRPRPCLMPMNKDFPVYYIDPDNESVEIWGVVTHSLIEHPVCLR"

CDS 53394..54632

/note="error-prone, lesion bypass DNA polymerase V"

/codon_start=1

/transl_table=11

/product="UmuC"

/translation="MYASCEQAFRPDLANRAVAVLSNNDGNIVARNYLAKKAGLKMGD

PYFKVRPIIERHNIAIFSSNYTLYASMSARFAAVVESLASHVEQYSIDELFVDCKGIT

AAMSLDAFGRQLREEVRRHTTLVCGVGIARTKTLAKLCNHAAKTWPATGGVVALDDGA

RLKKLMSILPVAEVWGVGHRTEKALATMGIKTVLDLARADTRLIRKTFGVVLERTVRE

LRGEACFSLEENPPAKQQIVVSRSFGQRVVALADMQQAITGFAARAAEKLRNERQYCR

VISVFIRTSPYSVRDTQYANQATEKLTVATQDSRTIIQAAQAALARIWREDIAYAKAG

IMLADFSGKEAQLDLFDSATPSAGSEALMAVLDGINRRGKSQLFFAGQGIDNSFAMRR

QMLSPDYTTDWRSIPTATIK"

CDS 54783..55574

/codon_start=1

/transl_table=11

/product="zinc metalloproteinase Mpr protein"

/translation="MNLPTPETYDELQRAYDFFNEKLFSNELPPCLITLQREKRTYGY

CSFKRFVGRESGYTVDEIAMNPVYFSIRTIKATLSTLVHEMVHQWQFHFGEPGRRGYH

NKQWAARMERVGLMPSDTGEPGGRKVGQSMTHYIIAGGPFDMACDELLTGHFRLSWMD

RFPPYQPKPGAVLSPTGKGYIDDEEDDSEHEQEVEEGRDPVELDDEIIEAMRFVTPPP

EAPVNKTNREKYSCPVCHINLWGKPGIVVYCGGEHCNKAALVVLK"

CDS complement(55589..55930)

/codon_start=1

/transl_table=11

/product="hypothetical protein"

/translation="MAQKNRISETEWKQLLPQMASFAHITTDIGYSVLVKGEKSSDVA

TRVGRSKQNISSTVKRIWDLYQNTTLKAENGEPLKLVQVWIPASLAETVLKEAAKYSI

NNITTSEMEKK"

BASE COUNT 13356 a 14874 c 14755 g 13504 t

ORIGIN

1 atggataagt tgctgaacaa aaagataaaa gttaagcagt ctaacgagct taccgaagct

61 gcttactacc tctcgctaaa agcaaagcgc gttctctggt tatgtcttat gcagacgtat

121 ttcacagctt cagtaagcga agatgatgat gagatggctg tactcggtga ctctactttc

181 aaagtaaagg tggctgacta tgagcaaatt tttcaggtaa gccgtaacca ggctatcaag

241 gatgttaaag aaggcgtgtt tgagttaagc cgttctgcgg taatctttta cccgaaagaa

301 gggagttttg actgcgtcgc gcgcccctgg ctaacagagg ctggcagccg atcagctcgt

361 ggtatctggg aaatcgaatt taaccataaa ctcctgcggt acatttacgg cctgacgaac

421 cagttcacca cctactcgct ccgcgattgt ggcagtcttc gaaatccacg gacgatccgc

481 ctttatgaaa gtcttgctca attcaaatct tcaggcttat gggttactac tcatgcttgg

541 ttaaatgacc gtttcctttt gccggaatcc caacagaaga acttggcaga gttgaaacga

601 tctttccttg atcctgcact caagcagata aatgagaaaa cacctttact tgctaagtat

661 agtattgatg attcaggaaa atttctgttc tcaataattg ataagcaaaa tcccgtctga

721 cataaatcag cacacatgag cctgtcattt gacaaatttt tgtcatgaag atgggcggat

781 ttccacacag caccggcgcc cggcaaggtg ggcggattcc cacacggcac cggcgcccgg

841 caaggtgggc ggattcccac acggcaccgg cgcccggcaa ggtgggcgga tttccacaca

901 gcaccggcgc ccggcaaggt gggcggattc ccacacggca ccggcgcccg gcaaggtggg

961 cggattccca cacggcaccg gcgcccggca aggtgggcgg attcccatat cgacatgtat

1021 gtagcttgtg ttatccgtgg attgtgcagc tcagcgggtc gctggtcgta tggcgtagtg

1081 tcccccgtaa ccggccgcgt gcggccgcta actcgcagta cggcgccgcg acccgaaggc

1141 gggccgccgt tcccgcgcgc aggcgcgcgg cgcccactgc gcacccccgt gggggacata

1201 cggcagctgt gtggcggtga gcgggattag ggctttgcag ggagggggct gggtcgggcg

1261 atacgttcag cattgcggtt tccggcgatt tgcggccggt gcccgtttaa ctccggcgtg

1321 gtcgccttcc atgccctgac ggcataagaa aataaaaccg ccatgctgcg gtcattcatg

1381 attttgtggt gtagcgataa atagtcatgc gagaaacgtt gaagcgctta gcaactgcac

1441 caactgtcat ttcaggatca gcaagtaaga ttctaatttg tttaacatct tcttcagaaa

1501 gtgacggttt tctccctccc acacggcccc ttgcgcgtgc agctgcaagg cctgagcgcg

1561 ttctttcaat attgcggttg cgttcaaagc tagagaatat cgccatcaga tgagtataga

1621 tttcccctat aactggcgca tttgtgtcta ttctgtcctt gatggctatg aaagttattc

1681 cgcgtttctt caggtcgtcg agtaaagtaa tgacttgacc caatgaaccg ccgagccgat

1741 ctagtgccca aactactagg gtatctccct cgcgcaatgc tttcaggcag ttctccagtt

1801 ccggcgcacc ttttttgtcg cgctttgggc cgctacgtga ggtctgatcc tgatagattt

1861 gctcacatcc agcttttgtt agttcgtcaa cctggtgcgc cacatcctga agatgcgtag

1921 atttacgtgc atagccgatt ttcattcttt tctcgctaat tagttatggg gttattgtta

1981 tgttgataca gtaacgagtt ttgttacatg aggggagtca tttttcggga gaagtcagga

2041 cttttcaaga ctgtcacaaa aaccatcgtt tttgatacat taatttaacc aataggttgc

2101 agatcaaatc gcctgtaaca gcctttctgg ctgtttgtcg ttttcagaag acggctgcac

2161 tgaacgtcag aagccgactg cactatagca gcggaggggt tggatccatc aggcaacgac

2221 gggctgctgc cggccatcag cggacgcagg gaggactttc cgcaaccggc cgttcgatgc

2281 ggcaccgatg gccttcgcgc aggggtagtg aatccgccag gattgacttg cgctgcccta

2341 cctctcacta gtgaggggcg gcagcgcatc aagcggtgag cgcactccgg caccgccaac

2401 tttcagcaca tgcgtgtaaa tcatcgtcgt agagacgtcg gaatggccga gcagatcctg

2461 cacggttcga atgtcgtaac cgctgcggag caaggccgtc gcgaacgagt ggcggagggt

2521 gtgcggtgtg gcgggcttcg tgatgcctgc ttgttctacg gcacgtttga aggcgcgctg

2581 aaaggtctgg tcatacatgt gatggcgacg cacgacaccg ctccgtggat cggtcgaatg

2641 cgtgtgctgc gcaaaaaccc agaaccacgg ccaggaatgc ccggcgcgcg gatacttccg

2701 ctcaagggcg tcgggaagcg caacgccgct gcggccctcg gcctggtcct tcagccacca

2761 tgcccgtgca cgcgacagct gctcgcgcag gctgggtgcc aagctctcgg gtaacatcaa

2821 ggcccgatcc ttggagccct tgccctcccg cacgatgatc gtgccgtgat cgaaatccag

2881 atccttgacc cgcagttgca aaccctcact gatccgcatg cccgttccat acagaagctg

2941 ggcgaacaaa cgatgctcgc cttccagaaa accgaggatg cgaaccactt catccggggt

3001 cagcaccacc ggcaagcgcc gcgacggccg aggtcttccg atctcctgaa gccagggcag

3061 atccgtgcac agcaccttgc cgtagaagaa cagcaaggcc gccaatgcct gacgatgcgt

3121 ggagaccgaa accttgcgct cgttcgccag ccaggacaga aatgcctcga cttcgctgct

3181 gcccaaggtt gccgggtgac gcacaccgtg gaaacggatg aaggcacgaa cccagtggac

3241 ataagcctgt tcggttcgta agctgtaatg caagtagcgt atgcgctcac gcaactggtc

3301 cagaaccttg accgaacgca gcggtggtaa cggcgcagtg gcggttttca tggcttgtta

3361 tgactgtttt tttgtacagt ctatgcctcg ggcatccaag cagcaagcgc gttacgccgt

3421 gggtcgatgt ttgatgttat ggagcagcaa cgatgttacg cagcagggca gtcgccctaa

3481 aacaaagtta gatgcactaa gcacataatt gctcacagcc aaactatcag gtcaagtctg

3541 cttttattat ttttaagcgt gcataataag ccctacacaa attgggagtt agacatcatg

3601 agcaacgcaa aaacaaagtt aggcatcaca aagtacagca tcgtgaccaa cagcaacgat

3661 tccgtcacac tgcgcctcat gactgagcat gaccttgcga tgctctatga gtggctaaat

3721 cgatctcata tcgtcgagtg gtggggcgga gaagaagcac gcccgacact tgctgacgta

3781 caggaacagt acttgccaag cgttttagcg caagagtccg tcactccata cattgcaatg

3841 ctgaatggag agccgattgg gtatgcccag tcgtacgttg ctcttggaag cggggacgga

3901 cggtgggaag aagaaaccga tccaggagta cgcggaatag accagttact ggcgaatgca

3961 tcacaactgg gcaaaggctt gggaaccaag ctggttcgag ctctggttga gttgctgttc

4021 aatgatcccg aggtcaccaa gatccaaacg gacccgtcgc cgagcaactt gcgagcgatc

4081 cgatgctacg agaaagcggg gtttgagagg caaggtaccg taaccacccc atatggtcca

4141 gccgtgtaca tggttcaaac acgccaggca ttcgagcgaa cacgcagtga tgcctaaccc

4201 ttccatcgag ggggacgtcc aagggctggc gcccttggcc gcccctcatg tcaaacgtta

4261 tgcagccaaa tcccaacaat taagggtctt aaaatggtaa aagattggat tcccatctct

4321 catgataatt acaagcaggt gcaaggaccg ttctatcatg gaaccaaagc caatttggcg

4381 attggtgact tgctaaccac agggttcatc tctcatttcg aggacggtcg tattcttaag

4441 cacatctact tttcagcctt gatggagcca gcagtttggg gagctgaact tgctatgtca

4501 ctgtctggcc tcgagggtcg cggctacata tacatagttg agccaacagg accgttcgaa

4561 gacgatccga atcttacgaa caaaagattt cccggtaatc caacacagtc ctatagaacc

4621 tgcgaaccct tgagaattgt tggcgttgtt gaagactggg aggggcatcc tgttgaatta

4681 ataaggggaa tgttggattc gttggaggac ttaaagcgcc gtggtttaca cgtcattgaa

4741 gactagtcct ttgcataaca aagccatcaa accggacgcc agagattccg cgcctgttgc

4801 gcatggcttc gccattttat gcgcaatagg cgcgccaccc tgtcgccgtt tatggcggcg

4861 ttaacccaaa ggagtatcgt gaaaatatca ctaatggctg caaaagcaag aaatggggtt

4921 attggctgcg gctcggatat cccgtggaac gctaaaggtg agcagctgct ttttaaagca

4981 ataacttaca atcaatggct cttagtcggc cgtaaaacat ttgaggcaat gggggctctc

5041 ccaaatagaa agtatgcagt tgtcagccgc tcaggatcgg tagctactaa cgatgatgtg

5101 gttgtgtttc catctataga agcagcaatg agggagctaa agactcttac gaaccatgtt

5161 gttgtttctg gtggtggaga gatctacaag agtctgatcg cccatgccga cacgctacat

5221 atctcgacaa tagattccga gccagagggc aatgttttct ttccggaaat ccccaaagag

5281 ttcaatgtgg tgttcgagca ggaatttcat tcaaatataa attatcgcta tcaaatctgg

5341 caaaggggtt aaccatccaa gccatcggac acattttgct tcgctgcgct caaaacgcaa

5401 aatgtgccgc tgcttagcgg cgttagatgc actaagcaca taattgctca cagccaaact

5461 atcaggtcaa gtctgctttt attattttta agcgtgcata ataagcccta cacaaattgg

5521 gagttagaca tcatgagcaa cgcagtgccc gccgagattt cggtacagct atcacaggca

5581 ctcaacgtca tcgagcatca tctgggatcg acgttgctgg ccgtgcattt gtacggctct

5641 gcactcgacg gtggcctgaa gccatgcagt gatattgatt tgctggttac tgtgactgca

5701 cagctcgatg agactgtgcg gcaggctctg ttcgtagatt tcctggaagt ttccgcttct

5761 cccggccaaa gtgaagctct ccgtgccttg gaagttacca tcgtcgtgta cggcgatgtt

5821 gttccttggc gttatccagc cagacgggaa ctgcaattcg gggagtggca gcgcaaggac

5881 attcttgcgg gcatcttcga gcccgcgaca accgatgttg atctggctat tctgctaact

5941 aaagcaaggc aacacagcct tgccttggca ggttcggccg cggaagattt cttcaactca

6001 gtcccggaaa gcgatctatt caaagcactg gccgacacct tgaaactatg gaactcacaa

6061 ccggattggg caggcgacga gcggaatgta gtgcttactt tgtctcgcat ttggtacagc

6121 gcagcaaccg gcaagatcgc gccgaaggat gtagctgcca actgggtaat ggaacgcctg

6181 cccgtccaac atcagcccgt gctgcttgaa gcccagcagg cttaccttgg acaagggatg

6241 gattgcttgg cctcacgcgc tgatcagttg actgcgttca tttactttgt gaagcacgaa

6301 gccgccagtc tgctcggctc cacgccaatg atgtctaaca gttcattcaa gccgacgccg

6361 cttcgcggcg cagcttaatt caggcgttag atgcactaag cacataattg ctcacagcca

6421 aactatcagg tcaagtctgc ttttattatt tttaagcgtg cataataagc cctacacaaa

6481 ttgggagata tatcatgaaa ggctggcttt ttcttgttat cgcaatagtt ggcgaagtaa

6541 tcgcaacatc cgcattaaaa tctagcgagg gctttactaa gcttgcccct tccgccgttg

6601 tcataatcgg ttatggcatc gcattttatt ttctttctct ggttctgaaa tccatccctg

6661 tcggtgttgc ttatgcagtc tggtcgggac tcggcgtcgt cataattaca gccattgcct

6721 ggttgcttca tgggcaaaag cttgatgcgt ggggctttgt aggtatgggg ctcataattg

6781 ctgccttttt gctcgcccga tccccatcgt ggaagtcgct gcggaggccg acgccatggt

6841 gacggtgttc ggcattctga atctcaccga ggactccttc ttcgatgaga gccggcggct

6901 agaccccgcc ggcgctgtca ccgcggcgat cgaaatgctg cgagtcggat cagacgtcgt

6961 ggatgtcgga ccggccgcca gccatccgga cgcgaggcct gtatcgccgg ccgatgagat

7021 cagacgtatt gcgccgctct tagacgccct gtccgatcag atgcaccgtg tttcaatcga

7081 cagcttccaa ccggaaaccc agcgctatgc gctcaagcgc ggcgtgggct acctgaacga

7141 tatccaagga tttcctgacc ctgcgctcta tcccgatatt gctgaggcgg actgcaggct

7201 ggtggttatg cactcagcgc agcgggatgg catcgccacc cgcaccggtc accttcgacc

7261 cgaagacgcg ctcgacgaga ttgtgcggtt cttcgaggcg cgggtttccg ccttgcgacg

7321 gagcggggtc gctgccgacc ggctcatcct cgatccgggg atgggatttt tcttgagccc

7381 cgcaccggaa acatcgctgc acgtgctgtc gaaccttcaa aagctgaagt cggcgttggg

7441 gcttccgcta ttggtctcgg tgtcgcggaa atccttcttg ggcgccaccg ttggccttcc

7501 tgtaaaggat ctgggtccag cgagccttgc ggcggaactt cacgcgatcg gcaatggcgc

7561 tgactacgtc cgcacccacg cgcctggaga tctgcgaagc gcaatcacct tctcggaaac

7621 cctcgcgaaa tttcgcagtc gcgacgccag agaccgaggg ttagatcatg cctagcattc

7681 accttccggc cgcccgctaa atatctcctt ttgggttgtt aataaaacat ccaataagtt

7741 gactgtgcgt gaaaaagaaa gttttgtgtg atggcgttga agatcgcacc gttaagctct

7801 tatgtgggat ggtgcagagc tcgacgacta ccgataaaac gcaaccgccg caaacagaca

7861 agaaaaagcc ccaactgata acagttgggg cttcagtatt gtgattggtg gagcaatagc

7921 accctgaacc caaaaccttc tcgctcaacc ggtagtggct gataacaact cgtgagggct

7981 attgcgggtt aagcatttag gggtcgatag agaaaacggg aaataaagca cggtaagaca

8041 gcggcaggcc atgccatgct tggtctttct ggaatggtgt ccagttgaag tacgcctcaa

8101 ccgggcgtca ggcaccccac ctatgttgcg tcagattagg atcgtttttc gccttttctg

8161 acgatctgcg ctcggcctct caggcttcaa cctgacagat caaccgctag cgtgctttat

8221 tttccgaatt ctgagacgac cccttgctgg ccgttggcac ggagggccag gacgcgcgcc

8281 cggacatgaa cgagcgcgag ttcttcttca caaaaatcat ctgggcgatg gattacacgc

8341 acatgaaatc actcaggctt gcagctgaag attttcccct ggcgcttgcg acggccaaga

8401 tcctgccgtg gccgtgggat gaatcaagct accggagtgc tttagccgat ataggcagcg

8461 ctaagggcaa tccgtgggta caggatatta accaccgcgt tactttgtgg ttgccctggc

8521 ggataggctt tgtacgagga gggaaccatt ctattgccag cggcgttctg gccggtgagg

8581 gtgaggtaat acccgatacg gtttatgata tgcggtattt gctcgatatt gtcagcactg

8641 acgggtatta ctggtacatg agcgggaaga tctgcgagag agtcagtgac taccgtacag

8701 ccgccttttt tgaaataggc cgcctgctta cactgtgatt ccttcaaaat atctcaacga

8761 aatttcccga cacctgttta agctatcttg ctgatatttc tgtttttcga tttttgccgg

8821 cgcgatgccg gcgacgttgg ccagctgccc cgtgcgcgat actgagatca gtttctttga

8881 acaaaaaaaa ccgcctttcg gcggtcagta ttcagtagct ggaacggtag tctctccccc

8941 gattccagcg gtcttgccag tgttcgagga atgattcagc gagcttaggc atgttccaga

9001 tcaccaccgc attctccgag tttttcgttt cggccgcttt ggtgaaatta aagctgccag

9061 tttcaacggt cacattatcc acgatgatca ccttgtcatg ctggataggg aaatcactgt

9121 cagtacgcaa agggatgccg ctgttcgcta tgtagttcat ggccgcaatg ctggcgcgcc

9181 ccgtattgcc cctttcatca attacgattt tcacatcaac tccccgtttt ttggctgcta

9241 ccagcgcctt cataatatcc ggggcggtaa aagaataagc catcatccgt atcgaggttt

9301 ttgcggagtc aatggcgctc aggacgagaa cgcgggcgct tccttcaggc gagtagccaa

9361 cctgaacgga tggttccacc gcaaaggcgg gcagggcggc tgtcgtaaat gctgcggcca

9421 gaagccaggt tgctaagttt ttcattacag gctcccgttc acacactgca ttttatattc

9481 agggtcaaaa taaatttctt tgattttccg cttttccata tgcaccacaa cgtcaatggt

9541 ggagtagagc attcgcataa tatcgctcat atcgagcata cggccgatag gggtcgcctt

9601 gataagcagc ccaatacggt taaaggcatc gcgcgcagag ttagcgtgcg ttgacataac

9661 accgcctgga tggccggtat taagtgcttt aagataatcc cacgcagcat catccctaag

9721 ctcagtcatg atgatacggc ccggtgtcag acgcatacag gctcgcaggg catcagtggc

9781 gctgacgcgg ccgatctttc ctgcatcgcc gtacatcata taaacggctt ctacaacgtg

9841 atcgaccgtg acttcgtgaa cgtcctctaa aataattaca cgctcgtctt tatgtagcga

9901 ttttaacagc gcgcgcgtga gtaccgtttt ccccgacccg gtttcaccgc agatcacgat

9961 agtgcgtttc ttctcaacgg cggtttgcag gaatgcgggc catttttcgc tgctgtgcag

10021 ctctttaagg aaaaaatcat catccgttag gctttgcttg ctgccggtaa tcttccggca

10081 gtcactgaaa atcccctcgc tggtcagctg ctccagattt ttatcggccg ccaaatcctt

10141 acgaaacgct acggccgttg taccgtcaat caccgcaggg ggcagacaga taacgcccct

10201 gatcccgcca ggcaggatca cgtcattaat ggcctgcatg gtcagcttgt tgctgctcac

10261 caacgattta gcaaggttcc taataaaatc tgccgtaatt gccgcgttct gcacaaccct

10321 gcggccgctg aacgtatcac agataacttc ctgaaagcag ttaatgcgaa tttcaaaaac

10381 agtaggatct tctaaatact cgcgcagtgg gccaagttga tagaaagctg catcagtcat

10441 gattactcct gaagaaaagc gggcgctaag cgcccacttt tttagttgtc tgcgagcgta

10501 taaacgccgc tgaaatcgag gtcgcgggca acaaaaatgc tcaccgcatc accctgctga

10561 tcgtagaggg taggggggat agacatgtaa gagcgaagtg cttcagacgc cagctgctca

10621 ccgctgtttt ctgtgctgtt gtactgaatg ttattactct gcgtctggtt aaccagcgcc

10681 gttaaggtgt cagagaacaa cgaaatcatg atcgcaccac gcagacgctc ccacatatgg

10741 gtatccacct ggcccggaat ccccgcgctg ccgagtgagt tcgttccggc actgtcaata

10801 ttaacgattg tcccgtcctg gtcattgcgg atacgctccc agagaacaaa cacgcgcgcc

10861 tggccgtctt tgataccacc ggtaatctgc ccgtcaaccc atgagccttt atcaatcagc

10921 ctaacgagtc catcagctga gtaaacgtcc tgtgaaaccc ggcaggaaac ctgacccgga

10981 acagtggtat ccagctcggt gccggtacca caggggatca ttttgccttt cggaacagtc

11041 aggctgggat tagccatgac tccagcgcgg ctagccttca gccttgcagg agtcaggttt

11101 ttagcgagtg ctgaactacc ttcgcttgtt tcgttgtcct ggggctgcac tccgggacta

11161 ttgcttgtag ccgcctgatt agtctgggcc agctcgccgc ccagacgacg ctgcatagcc

11221 agttcttcag gcgaaggttc tttacgctta ttagaggtac gcgcggcggt attgctgctg

11281 cccgcatctg catctgcctg tgcggcagcc tgcacagcac gggcatcagt ggcgctattc

11341 tgcgcagttg caggtttatt aacatcagga tcgctgttaa agctgtagtt tggcagtgta

11401 ttggcctgtt gcgctttacc accgtcttta tcagcttcag ctttagccgg ggtgcgaatt

11461 ttacccatga ccgtaatccc gatgaatacc aaagcaagca gcgccatcag tatgacaaag

11521 gctttcatac caggagccga acggcggtta ctgcctttaa atccgccacg ctcgctttca

11581 aattcaccgt ctccggtgtt ttcatcgagt tcctgatcta catcgacact tttacgggcc

11641 atcagttatc ctccccaatt tgaaccctgc gcacatccgg ggaagccgta ccggttgcta

11701 ccgcaccggc gcccggcgcg aaattattat tacgaacgcc aacgacttta tcgcccagac

11761 gaatacgcca ctctttagcg acggtttcca cctcgatgat gttgcggttc tcacccacaa

11821 catgagagtt aggcagcgtt tctttgccac tggccgagat catgtagacc tgcggtaact

11881 ccgcattggc cggaaactca aaccgggtaa agcggtagtt atcccagacg tgaaccggct

11941 ggatgctgcg catttcaggc tgttcgctca ttacgtactg atagttcttc gcccccgcaa

12001 aagccgtctg cttcagcttc tgcgtaatgc gttttttatc agccgcgctt ttggcttttt

12061 cctgctgctc aaacggatat tcataggtca gctgaagaac ggcctggcgc acagcccacg

12121 gcgtttcaat aaaggatttt gataccgtac cgtctgcatt tttcttcgtt tcttcaccga

12181 tgaaatggag gacgatgtta taggtgcgct tatcggtgac gatcaccagg ttggtatcac

12241 tcatggcctg tttcggcttc acaaaaaaat ggttcatttt gtgcgcaaac gtccagcttt

12301 cagaatcgcc aaaagcatga gtgatatagg tttcgtcagg cgcgacaaca atgtgggtag

12361 ccacaccggc gatagcgtca attttgacca cattaacagg gttataaaca acgcttttaa

12421 tgcgatagtc ataaggagaa ttgcggccaa cctcaagcgc cataacgtta gtggccgcgc

12481 ctcccaggac tgacaaaacg actgctgaaa gaagtagttt tttcatgggg cagccctcag

12541 ttaacttcag ggttgacgcg ataactcgtc acgcggaaac ccagcgggtt gacataacgc

12601 tgctcagcat tcatcgccag cgatttatat tcatacccca taatggcaat ccagcgctgc

12661 ggctgatcat caacgggatt gctgcgcacg cggcgaaccg tagtaaagcg tatcgttgct

12721 acgccgtgcg gtttatcgag gatcacagag ttaatcttca cgcgggtcgt ttcactgtcg

12781 cccagaacct tatcaagacc gttgcggccc ttgaacttgc tctggtaaga ctctgccacg

12841 ttcggcgtgg acattaagcc aacggccgta tagtcgacct gaactgaata gaagtcatag

12901 ctctcacggt gaatgacata ttgtgtcagc cagaacttat caatttcgtc accataagag

12961 gtctggtcgc gggtcagctt gacctgctgt acttcgtgag tggcctcgtt gagcgttagc

13021 agatgtgcgg ggattggctg gctgtactta tgcaccacgt aaccaactaa agagagtgca

13081 aaaacagtta ccaccgctga accggtggca accatccagg cggtacgccg ggacttcagc

13141 acttcatcca tcagatcaac ttcaagccct ttacggcttt cgttgaactc tttaatggct

13201 tcacgtgtaa gccctgtttt tttattagct ttcatttgca ccaccttgcg tatcaaccgg

13261 gattgttttg tttactggaa cggtgttgct ccagtccggc tccggtggcg gtttatgccc

13321 gctggaacag gcgctaatca gaagaactcc cataagcaat aagctgcgca ttacggcatc

13381 cattatcggt tgttgaacgt actaattata ataagtcgcc gaaattgcaa tttcaacgac

13441 ttttgtttta attttatgca gccttcttcc cgcgactgcc ccttgcctta ccacttaaat

13501 tagaaccact gcttcctccg ctgttgctgc cacttcctcc acctgccgat tgctggccgc

13561 cgccactgtt accgcctctg aacgcattac cgccaaacat gccgtggctg cccatattgc

13621 caagcgcctg catggaagaa cccgtagagc gtgcggcgtc agcaactccg gcgctgatac

13681 cgctacccca gctggcggca atttgcggaa tctggaacag aacgaaaacg gaaatgaccg

13741 tcagtaataa ggcggagata gaaccagtga ttgatgagta agcggcatca gagttcatcg

13801 aggacaggag gttgtcgaac atctgcatga tgaaaccaaa cacgagcgca agaatgacga

13861 cgacaaggcc atagttaatg accgacgcca gccagcgagc aaagatgttt tttgtcgctc

13921 cccacagcag gcagaagatt gcaatcgggc caaaacaaag cgtaacggcc agaaggatct

13981 tagccatgat cacaaagccc gcaccgaggc cgcccagcac aacggtagca atcatcataa

14041 tgccgccaat ggcatacgcg gccaggccgc tcgatgaaaa cacgtctgcg gcttcccatg

14101 cggtgttgac gatcttgata cctttttcaa taccgctatc aataatcgcc ggtacgccac

14161 ttgcaccgac tttattaggg gcagacagta tcccggcaaa atcatccggc aggtgaagcg

14221 ccacgttgac cagttcctgt tgataccagc cgcccgccgt tgcaaagctc aggataaggg

14281 ctatggaaag atactcttta atcagcgaac tcaggctgtc gcccgccccc ggattaaacg

14341 ctgagtacat cccctgaacc atcagcttga ttgtcagaca ggtggcaatc agaggcgtta

14401 catcagagat aatggtggca acattggcgc tcaccattga cgtaatcgcc ccgtctactt

14461 ttgcgaaaat gtctgcgact agggtgaatg ccatattgcc tccttactcc taaattttcg

14521 gtgaattgat ttcgccgtct tttttgccga acacaaacag ctgacgatcg gcttcgcgcg

14581 cgtttttgca atcaggtttc tgaagttcat ccgcgtcttt cttgcattcc tgaattgttg

14641 ccttgcgctc tttctcatgt tttttgtacc actccacgtc atgcgaggca tcgcaggcca

14701 ccaggaggaa agggataaca agcagtagtt ttttcataat tacctcagtt aattgaaggt

14761 gacgcggtaa cgtccccgcc ggttccaaaa acaaaattgt gggtggcacg ttctttctgc

14821 gcacgtaata gcttgtcctg tgactgctgc aacatgttca tcagattcag cttcgcctgc

14881 tcaccctgaa tagcaccctg tgacgtctgg atacgggcct gtaagtcagc aatcgatttc

14941 aggtctggag tcgttttaat ctgctccgtc agctcctgca tatcggttag ttcctgcatc

15001 tggttgttgt aggctttttc tgccataacg cggtcataag cgcctttttc agccagcttt

15061 ttgttcatgt aggtaattgc ttcgctgggc gtcatgtcgt caacttccgc attaaactgg

15121 cccatcatgc tgttaactga aggcgtgacg gaagaactgg agttcatggc gtcgctgtag

15181 atctccttcc agttgtccgg caaattattt gccagcgtgc tggtagacgt ccccagcaga

15241 tcgcccaggt tggttgtctt tgccatcgac tcatacatat ttttctgcgt ttgcagctgg

15301 cttttaagct gctccagctg ctgcgccatt tgctgaagct gctcgacctg tttagccagc

15361 tcagtagggt tggtcacgat gatgcctgcg gaagcgcttt gcgcgcctcc cagaatcagg

15421 ccggtggtca gcaatactgc cgtcagtgtt tttttcatgg tgttttgcct cgttgttaag

15481 ccgtcaggcg ccagtattct ttgagccata cttcagggtc attaccgagc cgttcaacca

15541 gctcatgcgc aatttcggcg ttttgtggtt caccggacaa cacgctaaga acgttgtcca

15601 ttgtcttaat atctgcatca atatcgtcgc tgttgcgagg gtagagattg aaagaagcaa

15661 tcgcagactg ttgcccttgt ttaaccagga actgtctgga gtgttcggta atcgacatca

15721 gcgcgtcgta ttcagcatca gtaaggaagg cataatcctc tcggatagct tccggatcgc

15781 gcaggcagat ttttgtgacg gtctgcgaca tgatcgtgcg gccaatacgg ctggacagcg

15841 catcgttcgg ctcctgcgtg gcaaacacat agatagcgtc tttcttacgg tcagttttga

15901 taccacgctt aacttcacgc tcgataaccg gatcgtcaag gtaggcgtgg aactcgtcaa

15961 agcactgaat gacgcggcgt ttgccgtcga tggagtcacg tacccggtac agaaggtaca

16021 tcatgagcgg agtacgggcc gggctggata cttcctcttt ggctgcgata aactcggata

16081 aatcgaagcc aaaaatatca ttcgcgctga gatccaggct gtctttatca ttgtcaaaca

16141 gccagccata ctggccttcg cgcgtccatt cgcgcagcag ccctttcagt gatacgccat

16201 tttccacaac ttcattcacg tacccgtcca ggatagtaac ggtgcgcgcc tcgcgtggaa

16261 taagtgagcc ttcccccata accgcatcaa cgccttcagc cagttcggta gccatcgttg

16321 cgctgatagg cccgttattg gtggtttcca cacaaatgcg gaacaggttt ttaatgaggg

16381 caatattgcg tttggtcggt tcaatctgaa gcggggcaaa cccggacggc ataccctgtt

16441 gcagaacttt atagtagcca ccaacgcttc gaatgaacgg ctccataccg cggtcacggt

16501 cataaacaaa aagccgcggg ttgtacttca ttgactgcgc cagcaggaag ttaagcagcg

16561 tggttttacc ttcccccgac atacccgtta ttaacgcatg gcccagcggg cgtttaccgt

16621 aggaaagttc ttcaagcggg gtcacatgga aattaaaata gagtggcgta ccgctgatcg

16681 tgcggaacat ggtcagcgct ggcccccacg ggttattgtc aggcttgcca cgcataaaat

16741 tgtggaacgg gctgaagtgc aggaagttcc atgagtttat cggaaccggg cgcggcgccc

16801 atttctggtt gccaggcagt ctcgcataat atgcagcctc agaggccagg ctgatagtcc

16861 cgccaaccac gccacagccg gttagcataa ccttcacacg acgcgctttg cgctgtaccg

16921 cgttttggtc attatcccag acatgcacgg ttccatgatg gtagcccatc acgaactctc

16981 tggacgtcag catatcgagc gcagtaccaa gctgtgccag ctggctttgc gcacggtcgc

17041 gcgtttcctg caaagatttt tcctgatgcg tcagaaacgt tttagctgaa gattcagaga

17101 ggcaagaaaa actctgcgtc agaaggtatt caaaatcggc ttctttaagc atgttaagct

17161 ggcctggctc tgtatcttct tcgtattcac gaaattcaat gccggtggta tagaagttgt

17221 gatctaccgt tctgatctgg acaacatccc cccacaggga gctaacaggg cggttgtcca

17281 tgatgtactc acggatacga tcacggcaaa caggcacgat ggcccattcc atatttgcga

17341 ggaaataaag gaactccagc gcttttgaat aagcgtgagc ctgcgaaggt tcaggctcgt

17401 tgcgttcgat aacaatggct tcgtcaaaaa tgtctgattc atcgacttca gcaagttctt

17461 cacgttcttt tttatcaggc gcaggaattt caacaccgcg tttgtcacga taatagatac

17521 ccaactgctg aatgccatac ggcttcattg cttccaggat ttgttcagaa atatcttcca

17581 gaccttcaag cgcctcattc tgcatttgct gaatttcgtc acgagtcggc ttttcaaatt

17641 tcgccagaaa cttctgtgtt ttatccccta cctgtttgta aataacggtc agataaaggt

17701 cattaatcag ctgcttggaa tcaccgtgca gcttacggtt atattgatca acataagcag

17761 ggaaaaaatg gtcatactca ccatccgggt actctttagc ctcatggtga tattcatgcg

17821 tccacagctc tacatggtct gttccgaagc tcttgaccag cgtattaagg tctttatgcc

17881 aggtgaccag ttcccgatct gatgcgcagt catgtgtgcg gccatccagc ttgaaaaaag

17941 ccatcagatc gccgttttcc atcgaaatca cgtaatcatt gaggtgataa gaatagggca

18001 aatacttttt atttactgat ggctccttac ggtaggcatc aatttttttt ggcttcgtag

18061 cggtggcagc tctcattatt ttaaaccctt acgtttgtag tcaacagagg aataagacga

18121 tcctccccac tgcttaaacg gagaatcaaa ccaattactg aatttggttt tcaaccacag

18181 gcccataatt cgaaacatgc ggtcgtcata ttttgttaat gcagctgaag gaatccacaa

18241 aaagacgaac accagaatcg cccacatatg aataatcata aaaagcgagg cagaaatcat

18301 gaatatcatc atagctacat tacgtggtac acccagcgcg cgaggtaagc gagtcgcacc

18361 tttgaaaagc ggtcttttcc cgtcaacgaa catctttgta gtcctcactt tgggggacaa

18421 aagtcccccc ttttcgcgtt gttcagcttc ctacgcccgt cagggaaacc agaaatgatg

18481 cagaaccaat accaatcagc gagattacga tacgaggaat aaagctggct gggattacgt

18541 gaagcatcca cataaagcaa ctaaccatga tcgcaatagc acaaccaatt ggaatccatg

18601 tgctcaacca cgtctggatt gatgtagcgg ttgattcacc agtatcggtg ccagcggcca

18661 gcgcaatttg cggcagggca atggacaaaa cgcccataac taccgcaggg ccatacttct

18721 taaacaacgt ggtcatactt tctccttact cactatcttc tgttgattgc agatttaacc

18781 ggcattcttc aacgccacac ttatctttca aaaactctgc cagtagcctt aaatcagccc

18841 aggttcgtag ctcatgctct ttgttaaccc taatggaaca tgcgatctgg cccagctgtt

18901 catcgtctat ctgaaaaacg ggataccact tctttaatga aacccgttct atcgttacct

18961 ccttcacttt tcctgcttca aatagctcca ggatctgtga ttgcaataca aggtacaaac

19021 gttccatcca taacctcatt cccccttcgc tgtttcctgt ctggttaaaa aggcatccgt

19081 attatttcgg ctgaaggcat cgccatcacc cgaaccaaaa acatcttgtt caccgtcata

19141 ctgcggcgcc gtactttttg cctgctgagg ctcagtcgcg gtgctgtcct cactggtctg

19201 gccgtcaggt agcagcgtag ggattttcaa atcagttgat tgacgcgcca cgttgataac

19261 tttcgtgaca tacccgttag aaatcccgtt tgtgagtgag ccggtgttgt agcaggaaag

19321 cgcgtgtctc agcgcaacct gcccggctgg gtaggatttc agggcgctat cataacaggc

19381 tttaaggatg gtctggctcg cccgcaggtt gatgcagggt ttgaaaatat cgtcaaccga

19441 aagacccagg cccactaaat tatttgagtt aatttgtgca aggcccatat caaaactttt

19501 attatccttc agcagaactt tcgcaacgct gacggcctca gcttcagtac gcggttgctg

19561 ttttaactga atactaccgt taatattgat cctgtacggc ccatttgagg actcatggcc

19621 gacgatgtac gccatcgtta agggtgaaac atcaggcgca catctctgcg caagcctggc

19681 aacttcatcg gaggcaggcg cagcactggc acggccagca caagcaaggc aggccagagc

19741 gagaaccagg agttttggat gtttactcat ttatgtttca ccgcgtaatt gtaatttcga

19801 cgacttttgt ttatgataag ggctaatgag atcgtgtcaa gaagaaacga aaagaaaacc

19861 gctgaaggaa ggaaggtgat gctggttccc ctgaaatcag aaaaacgacc aaaaggcgaa

19921 cccgtgtatc gtgacccgga taaccctttt aatacgtgga ctggtatagg gaagcgcccg

19981 gcctggctaa ctgcaaaatt ggacgctggc attagcctgg aagccatgaa gatgcagggc

20041 gtagccaacc ccagagaaca tagaccagca aaataccgcg accccaggaa cgcagaaaat

20101 acctggtccg ggactggccg ccgacccaca tggctcaaag agctgcttga tagtggttta

20161 tcacttgatg atctgaagat ataaccggag ggtataaaaa tgggcgaact tattgatttt

20221 gcggaaaggc aaaaaagcag aaggaagaaa aaggcatctt ccattccgcc catttttaga

20281 aaatttcgtg tccatgcgat caggttactc gccagcatca tcaaatccgg ctcttattca

20341 gttgcttata ttgttaagaa aattacagga aagttaatca agttttacac aattttaacg

20401 attttcgttt ttgttgtcga atatattgca ggcgatatag gttacaaatc tatttataac

20461 gcagcgttat tattaatatt gcttaccgtt atcaacattc tggcgagtgt atatctgaac

20521 aaactgttaa ggacaaaaca atgaagaaac tcttaatacc tctgatagca gctggtagtc

20581 tgctttatat tcctgccagc catgctgaag atccctgcaa agttattatg tgcatggcgg

20641 gcaagctcac cggcgatagc ggcggaagcg agtgtaacag tgctgaagct gctttcttca

20701 atatcgttaa aaagaacaag cacggctttt tacccaacca cacgaaggat gccaggaagg

20761 cttttcttaa tgaatgcccg gataatggcg aaggtggaag taaccagtcg atgataagcc

20821 agatcataag taaatacggg aaagttcgct tataggcgaa ctaggaataa tctcaattta

20881 aggagccaac gtgaaaaaaa taatattaag tgcattagcc tgtacagctc tcttaactgg

20941 ttgcgtcagc caggataaag gtaatgcgat gcagagccag ctgaataacc agcaacgcca

21001 gattaacgaa ttatccgttc gtttgcagtc tgcggagtcc cggctatcaa agcaggaaga

21061 aaagctgcgc aacgaactgc tgcaatccag cggctattgc tatctgaatg gcgcccgcta

21121 ctcgaccggc accgtacttt acgggcggat ttgccagaat cagtcaggca gcgcttcgtg

21181 gcaggtatac agccgtcgct aaacaccagg gcggtttaac cgcccttttc tttaccctca

21241 gaaagcccac tatgaccatg atccccttcc ccactacaga aaaccttatt ctatgggctt

21301 gcagcgccat cgcactgctt gccgttgtat tcttccggcg ttcagtacgg cacaggcgac

21361 acaaaaggaa gcagcaaagt gcgcggcggg tgctggagcg cataaagacg ttgccgggct

21421 tcccacaaaa aattaactac ctgaggaaaa ttgatccttt tgtgtttgaa gaactgttgc

21481 tggaaggatt tgaagcgcat ggcttcagaa ccatcagaaa caaacgctat accggcgatg

21541 gaggcattga cggccaggta ataataggaa aatatcgcta tcttattcag gctaaacgct

21601 atcgcggcca tattgcttta cagcacgtac aggagttcga gaagttgctt aaacgtcata

21661 actgtcgcgg tctgttttgc cataccggga aaaccggcgc aggttcaaaa tctgtcagta

21721 ttgccagtga acggatggag attatcagcg gccagcgcct gatagatttg ctcacgcccg

21781 gcagctcctt cactatcgca accgccccgc agacgatgat gaagcgtacc gcagcaacac

21841 tagaaacgag caccattgtt aaagatgccg gtaaagaaaa tcgataccat gagagttaat

21901 taaatgaagt cagtaactat agaagcaaaa acatttgctg aaatgttagg aataacagaa

21961 ggtgagttaa tctttgccat taagaaaact ggcacattca aaaacaagac catcccacaa

22021 cctcatgagc cacataaatc aaataataga tttttatatt cagacgtaat gaggtttata

22081 gaatcactaa aagacaaaga gaaccggtaa tgactccaac ttactgatag tgttttatgt

22141 tcagataatg cccgatgacc ttgtcatgca gctccaccga ttttgagaac gacagtgact

22201 tccgtcccag ccttgccaga tgttgtctca gattcaggtt atgtcgctca atgcgctgag

22261 tgtaacgctt gctgataacg tgcagctttc ccttcaggcg gggttcatac agcggccagc

22321 catccgtcat ccataccacg acctcaaagg ccgacagcag gcccagaaga cgctccagcg

22381 tggccaacgt gcgttaccta acaataaacc tgtttaaata tccagataaa aacattcaat

22441 ctgggtcaaa tgagtgatac agtttcaccc ataagaccca atggaggcaa tatgtctgaa

22501 tttgaattac tggcgcagga tctgcttgag aaagcagaag cggaagaaca actgcgacag

22561 gaaaatgata aaaagctgct cgggcaggtg ctggaaatct atgaccagaa gtacgtggct

22621 gaactgctta gaaaagttgg taaaaatgag tggagtcgcg agactcttaa tcgctggatt

22681 aatggtaagt gctcacctaa gacgctgacg ttagccgaag aggaacttct acgaaaaatg

22741 cttccggaag cgcctgcaca tcaccctgac tatgccttcc ggtttattga cctgtttgct

22801 gggattggag gtatacggaa gggcttcgaa accatcggtg gccagtgcgt ttttaccagt

22861 gaatggaata aagaggctgt gcgcacatat aaagctaact ggtttaacga tgctcaggaa

22921 cacactttca atctcgatat tcgtgaagtc acgctcagtg ataaacctga agtacctgaa

22981 aacgatgcct atgcttacat taatgagcat gtgccggatc atgatgtact tctagcaggt

23041 ttcccctgtc aaccgtttag ccttgcgggc gtaagcaaga aaaactcgct cgggcgcgcg

23101 catggtttcg aatgtgaggc tcagggaacg cttttcttcg atgtggcgcg tattatccgc

23161 gcaaaaaaac ctgccatctt tgttcttgaa aacgttaaaa acctgaagag ccatgacaag

23221 ggtaaaacct ttaaagtcat catggatacc ctcgacgaac tgggctatga agttgcggat

23281 gcagctgaga tgggcaaaaa cgatcctaaa gttatcgacg gaaagcactt tttacctcag

23341 caccgagaac gtatcgtttt ggtcggtttc cgccgtgatc tgaacattca ccagggcttt

23401 accctgcgcg atattagtcg tttttatccg gaacagcgtc cgtcatttgg cgaactgctg

23461 gaacccgtgg ttgacagcaa atatatactg acgccgaaac tctgggagta tctctataac

23521 tacgccaaaa agcacgcagc taagggtaac ggattcggtt ttggcctcgt taatcctgaa

23581 aataaagaaa gcattgcccg tacgctttct gctcgctatc acaaagacgg gtctgaaatt

23641 ctgatagacc gtggctggga tatggccaca ggtgaaacag acttcgcgaa cgaagaaaat

23701 caggcgcatc ggccccgcag gctgactccg cgagagtgcg cgcgccttat gggttttgaa

23761 aaagtagatg gcaggccttt tcgcattcct gtgtcagaca ctcagtcgta caggcagttc

23821 ggtaactccg tagtggtgcc cgtgtttgaa gccgtagcca aactgcttga accttatatc

23881 ctgaaagcgg ttaatgccga ttcgtgcaag gttgaacgaa tctgatcgct cctcccggta

23941 tttatgccgg gagataatct atggaatatc tgcgtaaagc cctgtcagct cagcaataaa

24001 cgcacctagc gtcattagct cagctctcac cgcctccggg tattttttgt gcagagatga

24061 tggcacgacc aatctgacac ccgactcccg catctcccta tattgagcca gagaaactcc

24121 ctcctggagt gtaaacagat gcacctgatg aattttatcg gcctcattca gtatctgacg

24181 ccagcgatcc ttacaggtag tcttgactgc cagcatgcgc agattttcta cgggaaactc

24241 agtatcgtgg taagcccctg cggaagggaa aaggaaatcg ggttttttat taccttctgt

24301 gatggcctgc gtcgcaaagt gtcgcaggcc gtgctcaatg aatagatgct ccaggtgcag

24361 ttccagcgac ttcccggctc tggatttacg gcgattgctg acagaattgg ccagcgcaat

24421 aaattcatcc acagagccaa atcctttccg gatgatatcc agaacatgca gttcctcaac

24481 caatagaaat atgtcgtact ccacgcgccg gcggtcaaga agttgctcat ccggatcaag

24541 ggaatttttc acataatggc tggctgcata ctgaataatt tcacttcccg acggaaagcg

24601 caggtgccag tcttcaggta gaatatattt atgatttact ggcgcttgct gtagagatag

24661 tccgcctaga atctgtcctg cggggccgga tataagcgct ccgggtataa cttcaccaat

24721 agcggtctca atgacgtcct cttcatcagt gctggcgcat acccaaatat ttacttcctt

24781 acagtccccc ccttgctcat caagcttgaa agccaggagc gtcagagccc ctgtattttc

24841 aggatcctga agtgggctgc ctctacccca gcgggtaatc cttttttcat tccgggtttt

24901 accaaaatga cggctgttat aataaattgc cctggcttcg ctgtcagggc aatcatgcga

24961 tgacacatgt gcggtgagaa aaaccgaagg gttcagttca cgggtatggt tgatagacgg

25021 aaagagtttt tcaacgatac ctgaagggat ataaagccct acctggtgac cacctgttgc

25081 gccggtatcg ttggcggaaa ggcgtttgat gtagacgaag taattctcac atgcgatctc

25141 aagtagccag ttgtggaaaa ccgacataag catcccctgt taccctgaaa ctctactcac

25201 cattttttca tgattatata caaacagtgt cattttcaga agacgactgc accagttgat

25261 tgggcgtaat ggctgttgtg cagccagctc ctgacagttc aatatcagaa gtgatctgca

25321 ccaatctcga ctatgctcaa tactcgtgtg caccaaagcg aggtgagcat ggcgacggag

25381 gctctgttgc aaagattggc ggcagtcaga ggtaggctgt cgctctgcgc cgatcaggcg

25441 gctgctgcga aatggtggtt gagcatgccc atggcctccg tcagcgccga gggcccaatg

25501 ccaaaagctc tctccacaag gcgcacctcg cccctgatgc cgggctgcag gcaccagggg

25561 cgagcctgtc ctttgcgcag ggctcgcatg acttcgaatc ccttgatcgt ggcataggcc

25621 gtggggatcg atttgaaacc gcgcaccggc ttgatcagta tcttgagctt tccgtgatcg

25681 gcctcgatca cgttattgag atacttcacc tgccggtggg ccgtctcccg gtccagcttt

25741 ccttcgcgct tcaattcggt gatcgctgca ccatagctcg gcgctttgtc ggtattgagc

25801 gtggcaggct tttcccagtg cttcaggcct cgcagggcct tgcccaggaa ccgcttcgct

25861 gccttggcgc tgcgggtcgg cgacaggtag aaatcgatcg tgtcgccccg cttgtcgact

25921 gcccggtaca ggtaggtcca cttgccccgc accttgacgt aggtttcatc caggcgccag

25981 ctcggatcaa agccacgccg ccagaaccag cgcagccgct tctccatctc cggggcgtag

26041 cactggaccc agcgatagat cgtcgtatgg tcgaccgaaa tgccgcgttc cgccagcatt

26101 tcctcaaggt cgcgatagct gatcggatag cgacaatacc agcgcaccgc ccacaggatc

26161 acatcaccct ggaaatggcg ccacttgaaa tccgtcatcg ttccgtccgt ccaatctccg

26221 ccaagcatgc tcaagcttca cgatttttgc aacagagccc acacgagtat tgagcatagt

26281 cgagattggt gcagatcact tctgatattg aactgtcagg agctggctgc acaacagcca

26341 ttacgcccaa tcaactggtg cagtcgtctt ctgaaaatga catttggtat ctctcataaa

26401 cggatgtttt tgagagaact atcttcggcc ttcacacgca cgaaaggcgg cgaagctccg

26461 ccgttaatcc gtccgccgga gatctcgccc aggcaggctg aaggccgagc aagcctgaca

26521 ggcccgaaaa gcccggcacg ggcgtcggcg gcgatgacgg cggcggcatt atccagggtt

26581 gatgatggaa gtggaggata tcgacaacct ctcgcgcaac caagacatcg cggtcggact

26641 gcaagtgatc ttgaagccac gggcccgtcc caccccgaca tggacctcga tgcccgaacg

26701 gacgttagat ttcgagttct aggcgttctg cgatgaaggt tggatcccag ccgggattga

26761 aagtgtcgac gtgggtgaat ccgagccgct cgtataggcc acgcaggttc gggtggcagt

26821 cgagccgcag cttggcgcac ccctgcgttc gcgcggcatg gcggcaagcc tcgatcagcg

26881 cggagctgac accccggccc gcatgtgtcc gtcgcaccgc gagcttgtgc agatatgcgg

26941 cctccccctt gagggcgtcg ggccagaact cgggatcctc ggccgacaag gtgcaacagc

27001 cgacgatgcc gtcgctgcaa ctcgcgacta ggagctcgga tctcaggacg aaggtctccg

27061 cgaatgtccg gtcgatccgc gcgacgtccc aggcgggcgt tcccttggcg gacatccacg

27121 ccgcagcgtc gtgcatcagc cgcacaacct cgtcgatatc acccgagcag gcgacccgaa

27181 cgttcggagg ctcctcgctg tccattcgct cccctggcgc ggtatgaacc gccgcctcat

27241 agtgcagttt gatcctgacg agcccagcat gtctgcgccc accttcgcgg aacctgacca

27301 gggtccgcta gcgggcggcc ggaaggtgaa tgctaggcat gatctaaccc tcggtctctg

27361 gcgtcgcgac tgcgaaattt cgcgagggtt tccgagaagg tgattgcgct tcgcagatct

27421 ccaggcgcgt gggtgcggac gtagtcagcg ccattgccga tcgcgtgaag ttccgccgca

27481 aggctcgctg gacccagatc ctttacagga aggccaacgg tggcgcccaa gaaggatttc

27541 cgcgacaccg agaccaatag cggaagcccc aacgccgact tcagcttttg aaggttcgac

27601 agcacgtgca gcgatgtttc cggtgcgggg ctcaagaaaa atcccatccc cggatcgagg

27661 atgagccggt cggcagcgac cccgctccgt cgcaaggcgg aaacccgcgc ctcgaagaac

27721 cgcacaatct cgtcgagcgc gtcttcgggt cgaaggtgac cggtgcgggt ggcgatgcca

27781 tcccgctgcg ctgagtgcat aaccaccagc ctgcagtccg cctcagcaat atcgggatag

27841 agcgcagggt caggaaatcc ttggatatcg ttcaggtagc ccacgccgcg cttgagcgca

27901 tagcgctggg tttccggttg gaagctgtcg attgaaacac ggtgcatctg atcggacagg

27961 gcgtctaaga gcggcgcaat acgtctgatc tcatcggccg gcgatacagg cctcgcgtcc

28021 ggatggctgg cggccggtcc gacatccacg acgtctgatc cgactcgcag catttcgatc

28081 gccgcggtga cagcgccggc ggggtctagc cgccggctct catcgaagaa ggagtcctcg

28141 gtgagattca gaatgccgaa caccgtcacc atggcgtcgg cctccgcagc gacttccacg

28201 atggggatcg ggcgagcaaa aaggcagcaa ttatgagccc catacctaca aagccccacg

28261 catcaagctt ttgcccatga agcaaccagg caatggctgt aattatgacg acgccgagtc

28321 ccgaccagac tgcataagca acaccgacag ggatggattt cagaaccaga gaaagaaaat

28381 aaaatgcgat gccataaccg attatgacaa cggcggaagg ggcaagctta gtaaagccct

28441 cgctagattt taatgcggat gttgcgatta cttcgccaac tattgcgata acaagaaaaa

28501 gccagccttt catgatatat ctcccaattt gtgtagggct tattatgcac gcttaaaaat

28561 aataaaagca gacttgacct gatagtttgg ctgtgagcaa ttatgtgctt agtgcatcta

28621 acgcttgagt tagacattat ttgccgacta ccttggtgat ctcgcctttc acgtagtgaa

28681 caaattcttc caactgatct gcgcgcgagg ccaagcgatc ttcttcttgt ccaagataag

28741 cctgtctagc ttcaagtatg acgggctgat actgggccgg caggcgctcc attgcccagt

28801 cggcagcgac atccttcggc gcgattttgc cggttactgc gctgtaccaa atgcgggaca

28861 acgtaagcac tacatttcgc tcatcgccag cccagtcggg cggcgagttc catagcgtta

28921 aggtttcatt tagcgcctca aatagatcct gttcaggaac cggatcaaag agttcctccg

28981 ccgctggacc taccaaggca acgctatgtt ctcttgcttt tgtcagcaag atagccagat

29041 caatgtcgat cgtggctggc tcgaagatac ctgcaagaat gtcattgcgc tgccattctc

29101 caaattgcag ttcgcgctta gctggataac gccacggaat gatgtcgtcg tgcacaacaa

29161 tggtgacttc tacagcgcgg agaatctcgc tctctccagg ggaagccgaa gtttccaaaa

29221 ggtcgttgat caaagctcgc cgcgttgttt catcaagcct tacggtcacc gtaaccagca

29281 aatcaatatc actgtgtggc ttcaggccgc catccactgc ggagccgtac aaatgtacgg

29341 ccagcaacgt cggttcgaga tggcgctcga tgacgccaac tacctctgat agttgagtcg

29401 atacttcggc gatcaccact tccctcatga tgtttaactt tgttttagcc accaatgatg

29461 ccctcacttt ccatgatttt ggtgggaatg gattttctta gcggcaactt actttcgttg

29521 tctatatcca tgttaaaggc gaaaaagtaa acctctgtct ccttctcaac ccacccaacc

29581 caccatgcga caccaggatt tgactcagtt cccacaccag aaaaaccagt ttttgaatgc

29641 actagatatt caggtgccgc ctccgttacc aaagcctctt ttactattag ctggttttct

29701 ttagatgctg acaatttatt taaatataga gactctagaa actccacttg attaactgcg

29761 gaaattctaa gctggccttc caaccagaat ttgtcaatgc caccactgat attctggttg

29821 ccataggaaa attttttaag gtatttctgc attcttactt cgccaacttc tctggcgatt

29881 tgttgaaata cgggaacagc tgaaacttgt attgcccctc ttaaggtcaa gtctctttcc

29941 cattgcttca tggctcttgg ctttccgtcc catttgaaaa cctgatgctc attctttatg

30001 acaccagttt ctaggccgat aattgcgttg gggatcttaa atgttgatgc tggaagatat

30061 tcctttgatg cacgagctaa gtcattggta gcgcaggatt tactgctact tttacaaagc

30121 acgaagacac cattgacggc ttcggcagag aactctttgt tccaagacgt attttctgta

30181 attgaaccag ctaatgccgt actcgaaaga cagcttgttg attatcatat ggcttttgaa

30241 actgtcgcac ctcatgtttg aattcgcccc atatttttgc tacagtgaac caaattaaga

30301 tcatctattt actaggcctc gcatttgcgg ggtttttaat gctgaataaa aggaaaactt

30361 gatggaattg cccaatatta tgcacccggt cgcgaagctg agcaccgcat tagccgctgc

30421 attgatgctg agcgggtgca tgcccggtga aatccgcccg acgattggcc agcaaatgga

30481 aactggcgac caacggtttg gcgatctggt tttccgccag ctcgcaccga atgtctggca

30541 gcacacttcc tatctcgaca tgccgggttt cggggcagtc gcttccaacg gtttgatcgt

30601 cagggatggc ggccgcgtgc tggtggtcga taccgcctgg accgatgacc agaccgccca

30661 gatcctcaac tggatcaagc aggagatcaa cctgccggtc gcgctggcgg tggtgactca

30721 cgcgcatcag gacaagatgg gcggtatgga cgcgctgcat gcggcgggga ttgcgactta

30781 tgccaatgcg ttgtcgaacc agcttgcccc gcaagagggg atggttgcgg cgcaacacag

30841 cctgactttc gccgccaatg gctgggtcga accagcaacc gcgcccaact ttggcccgct

30901 caaggtattt taccccggcc ccggccacac cagtgacaat atcaccgttg ggatcgacgg

30961 caccgacatc gcttttggtg gctgcctgat caaggacagc aaggccaagt cgctcggcaa

31021 tctcggtgat gccgacactg agcactacgc cgcgtcagcg cgcgcgtttg gtgcggcgtt

31081 ccccaaggcc agcatgatcg tgatgagcca ttccgccccc gatagccgcg ccgcaatcac

31141 tcatacggcc cgcatggccg acaagctgcg ctgagccatg gctgaccacg tcacccccaa

31201 tctgccatcg cgcgatttcg atgtgacaga ggcgttttat gcgaagctgg gctttgcgac

31261 gagttggaag gatcgcggct ggatgatcct gcagcgcggc ggtttgcagc tcgaattctt

31321 cccctatcct gacctcgacc cagctacgag ctcgttcggc tgttgcctgc ggttggatga

31381 tctcgatgcc atggtggcat tggtgaacgc ggcgggagcc gaggaaaaaa gcaccggctg

31441 gccgcgcttc aaagctccgc aactggaggc gagcggcctg aggatcggct acctgatcga

31501 tcccgactgc acgctggtgc ggctgatcca gaaccccgac tgaccgcatg cccgcgaaaa

31561 tcaagatttg cgggatcagc acacccgagg cgctcgatgc gaccatcgcg gcgcgggcgg

31621 actatgccgg gttggtgttc tatccagcgt cgccccgtgc ggttacgtcg aatgtcgcgg

31681 gcgctttgac atcgcgcgca gctggccaga tcgccatggt cggtttgttc gtcgatgcgg

31741 atgatgctgt catcgccgac gcactggtgg cagccaagct gaacgcgctg cagctgcacg

31801 gttcggaatc gcccgaacgc gtggcccagt tgcgcgcgcg gtttggcaag ccggtgtgga

31861 aggcgctgcc cgtcgccagc gccagcgatg tcgcacgcgc cgcagcctat gccggggcgg

31921 cggacttgat cttgttcgac gccaagaccc ccaaaggcgc gctgcccggc ggcatggggt

31981 tggcgttcga ctggtcgctg ctggccggat atcgcggtgc cttgccgtgg gggctggcag

32041 gcgggctaaa tccgacgaat gttgccgagg cgattgcgcg caccggagcg ccgctggtcg

32101 atacctccag cggcgtcgaa agcgcgccgg gcgtcaagga taccgacaag attaccaatt

32161 tcgcctttgc ggtgcgcttg gcctaaatcg cgtcgatcaa taggcgtcgt tcagcgcaaa

32221 gatcggcttg cgggtgcgcc actgccctcg ggtgaagtcg ggaaaatcta acgtgcgatt

32281 gccctcagca atcgattgtt ccgacagagg cgtgatcgcg ctccaggcca gcgcgtcgta

32341 aatgtcgatt ggcatcgggg ccttggcctt cagcgcctcg acaaaagcgt ggatcacgaa

32401 ccagtccatc ccgccatgcc cggcccctgc cgccagatcg gcgtagcgtt tccatagcgg

32461 gtgatcgtat ttcgcaaacc agccctcggc aggctcccag cggtgcggct gtgggctctt

32521 gccctccaga tagatcgact tgttgacgtc cgggtatagg aagtataaac cacctttttg

32581 ctcctcatcc gaagtatctt acctgaaatt ccctcactcg tttaccgctc aagccccaat

32641 tttaactgcc ggtccagcct aaaccgctct aataaggttc gatttggcgg taaaatctct

32701 agcctgatag ctcgagagat acaaactgcc ccaccgcccc gtttaaaagt tggcagtgtt

32761 gagcagtgtt ggatttgggg tcgtcagtca aagagacgac tctgtgatgg atcgaacagg

32821 ctgggagtca gtggcggcgc tcgttctggt ggcagctcac gctgcttggc ggcattcgcc

32881 ttggctgttt tctgtttcag atgcttgaga atctgctcaa tgaccttcgg atcttcgatg

32941 ctggcaatca ctttgacgtg accgccgcag tgttcgcaga cttcaatatc aatattgaag

33001 actcgcttga ggcgttgcat ccaggtcatg gcgcggtggc gctctgcagg actcttgtca

33061 cgccagttag tatcgagacc ttccgatttg tcgggcttct tgccccgctt ggcgggtgtt

33121 acttgaactc ggtgtttgct gttcggtgca aagacgccgt ggaagcgtgt gaggttgact

33181 cgcggcttag gtaccaacgc agcgagtttg gcgatgaagt ccagcggctc gaagatcaca

33241 tgggtggtgc cattgcggta cggagttttg agctcgtaac gcacctgccc attggcggtt

33301 aatgccagac gtttttctga aaccgctggc cgactaatgt agcgacacaa gcgctcaagc

33361 ttatcccgct gatgcgcttc ggccatcaca ccggcgtgta gcgagaaacc agcatggttg

33421 gctactcgac tgcttgagtc ggctttatcc tcacgccctg gcaaggtttg cagggtgaag

33481 actttgcgcc cttgctgggg gccgacggca atgcgatacg taaccgaagc accatgtaat

33541 tgagtcagcg tatcgtcttc gccctcttcc agtgtcaacc acgtattctc ggcatcacgc

33601 tccaaaatcc cacgcttttc catgcagcga gcgatgcgat ggctgagggt gtgagcgagc

33661 gtattcagct catcgtaagt gggtgccttg acacgatgga agcgttgctt gccatagtca

33721 tcttcggcat agacaccatc gagaaacagc atgtggtagt ggacattgag atttagcgcg

33781 gagccaaagc gttggataag agtcactgag ccagtttgtg cagaggcttt ggtgtaaccg

33841 gcttttttga tcagatgagt tgagagtgta cgatagacga tactcaagac ctggcccatc

33901 agctggggat ggcgagccag caaaaagcgt agctggaaag gaaagctgag cacccactgg

33961 cgaatgggct ccttggggaa gacttcgtct atcagcagcg ccgcactctc ggccatccgg

34021 cgggcaccgc agctagggca aaagccgcgt cgtttacagc tgaaggcgac cagacgctcg

34081 tgatgacaat cctcgcagcg aacccgcatg aaaccatact ccagacggcc acattggagg

34141 aggtcgttga attcttgttg gatgtagcga ggcaggtgtt gaccttgggc ttcgagtgag

34201 gctttgaagg ctgggtagtg ctgctcaacc agctggtaga gcagcgtctg gtcgggttgg

34261 tggcgttcgt aaccgtttgt ttgagtgggc gattgactcg ccgtggcgtt ccttgccagc

34321 gacatgggta tcctccgctg atactgtggt tatgtacagt atcagcggct tgcgttcaga

34381 cgtccagtct ggccctagac atcgctaaat ggggtcgtct cagaattcgg aaaataaagc

34441 acgctagcgg ttgatctgtc aggttgaagc ctgagaggcc gagcgcagat cgtcagaaaa

34501 ggcgaaaaac gatcctaatc tgacgcaaca taggtggggt gcctgacgcc cggttgaggc

34561 gtacttcaac tggacaccat tccagaaaga ccaagcatgg catggcctgc cgctgtctta

34621 ccgtgcttta tttcccgttt tctctatcga cccctatctc atctgcgcaa ggcagaacgt

34681 gaagacggcc gccctggacc tcgcccgcga gcgccaggcg cacgaggccg gcgcgcggac

34741 ccgcgccacg gcccacgagc ggacgccgca gcaggagcgc cagaaggccg ccagagaggc

34801 cgagcgcggc cgtgaggctt ggacgctagg gcagggcatg aaaaagcccg tagcgggctg

34861 ctacgggcgt ctgacgcggt ggaaaggggg aggggatgtt gtctacatgg ctctgctgta

34921 gtgagtgggt tgcgctccgg cagcggtcct gatcaatcgt caccctttct cggtccttca

34981 acgttcctga caacgagcct ccttttcgcc aatccatcga caatcaccgc gagtccctgc

35041 tcgaacgctg cgtccggacc ggcttcgtcg aaggcgtcta tcgcggcccg caacagcggc

35101 gagagcggag cctgttcaac ggtgccgccg cgctcgccgg catcgctgtc gccggcctgc

35161 tcctcaagca cggccccaac agtgaagtag ctgattgtca tcagcgcatt gacggcgtcc

35221 ccggccgaaa aacccgcctc gcagaggaag cgaagctgcg cgtcggccgt ttccatctgc

35281 ggtgcgcccg gtcgcgtgcc ggcatggatg cgcgcgccat cgcggtaggc gagcagcgcc

35341 tgcctgaagc tgcgggcatt cccgatcaga aatgagcgcc agtcgtcgtc ggctctcggc

35401 accgaatgcg tatgattctc cgccagcatg gcttcggcca gtgcgtcgag cagcgcccgc

35461 ttgttcctga agtgccagta aagcgccggc tgctgaaccc ccaaccgttc cgccagtttg

35521 cgtgtcgtca gaccgtctac gccgacctcg ttcaacaggt ccagggcggc acggatcact

35581 gtattcggct gcaactttgt catgcttgac actttatcac tgataaacat aatatgtcca

35641 ccaacttatc agtgataaag aatccgcgcg ttcaatcgga ccagcggagg ctggtccgga

35701 ggccagacgt gaaacccaac agacccctga tcgtaattct gagcactgtc gcgctcgacg

35761 ctgtcggcat cggcctgatt atgccggtgc tgccgggcct cctgcgcgat ctggttcact

35821 cgaacgacgt caccgcccac tatggcattc tgctggcgct gtatgcgttg gtgcaatttg

35881 cctgcgcacc tgtgctgggc gcgctgtcgg atcgtttcgg gcggcggcca atcttgctcg

35941 tctcgctggc cggcgccact gtcgactacg ccatcatggc gacagtgcct ttcctttggg

36001 ttctctatat cgggcggatc gtggccggca tcaccggggc gactggggcg gtagccggcg

36061 cttatattgc cgatatcact gatggcgatg agcgcgcgcg gcacttcggc ttcatgagcg

36121 cctgtttcgg gttcgggatg gtcgcgggac ctgtgctcgg tgggctgatg ggcggtttct

36181 ccccccacgc tccgttcttc gccgcggcag ccttgaacgg cctcaatttc ctgacgggct

36241 gtttcctttt gccggagtcg cacaaaggcg aacgccggcc gttacgccgg gaggctctca

36301 acccgctcgc ttcgttccgg tgggcccggg gcatgaccgt cgtcgccgcc ctgatggcgg

36361 tcttcttcat catgcaactt gtcggacagg tgccggccgc gctttgggtc attttcggcg

36421 aggatcgctt tcactgggac gcgaccacga tcggcatttc gcttgccgca tttggcattc

36481 tgcattcact cgcccaggca atgatcaccg cccctgtagc cgcccggctc ggcgaaaggc

36541 gggcactcat gctcggaatg attgccgacg gcacaggcta catcctgctt gccttcgcga

36601 cacggggatg gatggcgttc ccgatcatgg tcctgcttgc ttcgggtggc atcggaatgc

36661 cggcgctgca agcaatgttg tccaggcagg tggatgagga acgtcagggg cagctgcaag

36721 gctcactggc ggcgctcacc agcctgacct cgatcgtcgg acccctcctc ttcacggcga

36781 tctatgcggc ttctataaca acgtggaacg ggtgggcatg gattgcaggc gctgccctct

36841 acttgctctg cctgccggcg ctgcgtcgcg ggctttggag cggcgcaggg caacgagccg

36901 atcgctgatc gtggaaacga taggcctatg ccatgcgggt caaggcgact tccggcaagc

36961 tatacgcgcc ctaggagtgc ggttggaacg ttggcccagc cagatactcc cgatcacgag

37021 caggacgccg atgatttgaa gcgcactcag cgtctgatcc aagaacaacc atcctagcaa

37081 cacggcggtc cccgggctga gaaagcccag taaggaaaca actgtaggtt cgagtcgcga

37141 gatcccccgg aaccaaagga agtaggttaa acccgctccg atcaggccga gccacgccag

37201 gccgagaaca ttggttcctg taggcatcgg gattggcgga tcaaacacta aagctactgg

37261 aacgagcaga agtcctccgg ccgccagttg ccaggcggta aaggtgagca gaggcacggg

37321 aggttgccac ttgcgggtca gcacggttcc gaacgccatg gaaaccgccc ccgccaggcc

37381 cgctgcgacg ccgacaggat ctagcgctgc gtttggtgtc aacaccaaca gcgccacgcc

37441 cgcagttccg caaatagccc ccaggaccgc catcaatcgt atcgggctac ctagcagagc

37501 ggcagagatg aacacgacca tcagcggctg cacagcgcct accgtcgccg cgaccccgcc

37561 cggcaggcgg tagaccgaaa ggggtcgata gagaaaacgg gaaataaagc acggtaagac

37621 agcggcaggc catgccatgc ttggtctttc tggaatggtg tccagttgaa gtacgcctca

37681 accgggcgtc aggcacccca cctatgttgc gtcagattag gatcgttttt cgccttttct

37741 gacgatctgc gctcggcctc tcaggcttca acctgacaga tcaaccgcta gcgtgcttta

37801 ttttccgaat tctgagacga ccccagcaag taatcggcct gaattggcag tatcagcgcg

37861 cgtagtaagt catggatcgc cggttgcggc agtctgcgcg ccagattaat tacccggtcg

37921 aacttcagtt tatcctgctt gcgcttgtcg gtctgctcca tcagatttca tggccccctt

37981 attcatgctc atgctcatgg gtgtgttctt ttccggtatg gctctgttcc gcctgagacg

38041 tctgcggcat ggcgtaatcg tcgtaaatgc tgctgtcaaa gtcgtagtct gatgcttcgg

38101 cataatgctc gtaatcggca tactcctgcg cgctccactg ctgatcgtcg gcagcagcat

38161 aatcatgggc cagttctgca tcattttgct gtgcttcatg acgccgcagg ccaacggaat

38221 catccatagg gttctgctta agatgaaagg cgtcctctgc gttgctcacc ggctgataat

38281 cagtgccggt tgtcatgtta tgttcatcgg gtttctggtt aaacgccatg ctttcccccg

38341 tggcttctgg cagacctttt tcagctgatc gggtttctaa actggtatcg cggccaatat

38401 ccttaaacct ggcctcaagc ccaaagaaac ggtcaatttc tgcggccgtg gttttcgggc

38461 tgtcgcggct cacgctcgat gccaaagatt ttttatcgtc ggtaaaaatt tccacctcat

38521 gacgcgcacg cgaaatacca acataaaaaa cgtccttaga agtggtaagc gatttggtat

38581 ctatgttgaa caacacgcga tcacaggtaa gcccctggga tttgtggacg gtggttgcat

38641 aagcatagga aagataagaa gcctgttttt tgtccagctc aaccgtgcgc ccttttttgt

38701 cctcaagcgt cagtttttca ccctccacgg ttttcaccgt gaagcggtcg ccgttggcaa

38761 cgtccagcgt tttatcgtta cgcgttacca taaccttatc gcccggcgcc agttcggcgc

38821 tgactgcctg gtatacagac agcttggtgt gtgtacgcgg gctgaaagcg atctgctcac

38881 cgctgctgct ttcaaccgtc aatttgttgc ccggcccggt atcaagaacc tggtaagact

38941 cgccccgctt cataccattt ttgtaatcct gttcggggat aatgatttgc cctttactga

39001 aataacggct gtcgcggcgt tccgcctgtg tcgaatccac gcggtcaagt agcgtgaacg

39061 tttcgccggt tccggcaagc cccagattgc cccggatgta gtcattgagg gttttgcgtg

39121 aggcgttcgt accagagatt atcagggtgg catcctgttg ttctgaggac agagacaggt

39181 agcgatcggc tagttgagag agtcggggcg cttcttcctt cagttcgttc acgccggtga

39241 tatttttcag ggcgcgcgcg gcattacctt cagcggcata cttaaccgcc tcaagcaaaa

39301 cttcattctt ctgtcgctga atgtctttca tgtagctggt ctgcatacct gctttaatca

39361 gctgctcaaa aggcttaccg gcttctaccg ctttcgtctg tgacgtatcc cccaggaata

39421 ccgcgcgagc gttatgcttc tcgatcacct ccatcagctg tttcatctgt cgggcgggta

39481 taaccccggc ttcatcaatg aatacgactg atttttcatc cagcttttta tccttcgctt

39541 tgaggaaagc ggcaacggtg cgggccggta atccatcatc ttcaagcgct tttttctgtg

39601 tcccataggg ggccagcgcc gtgaccttca gcccttgtga ctccagcagc tctttagcgg

39661 ccatcgtcat atagctttta ccggtaccgg cgtaaccatg tgcggccaca aaccgatctt

39721 tgctcgtcac aatttctgta accgcgcgca tctgctcctt cttgagggtt ttcccggcaa

39781 gcagctggcc tgcaatctct gcggtcagct gtcgcggcat ctgcccccgg ccgcgtgatt

39841 cgatagtcag aatggaacgc tcaaggcgaa taccctccac ggtagtgacg cggtggctgg

39901 tctttttaag cctgccgttt ttaataccat catctaccgc aaaacgggct ttatccgcac

39961 gcatcccgct attcgtcagc gagtcgatcc actctttgcg cgtcagagtt tcggccataa

40021 ctgaagcacc gaccttcaga gttgattgat accgggcttc gccctcgatg atggcgccct

40081 tctgtaccgc cttcaggtac gctttttcaa catcggctat tgtggcatgg cccagcacct

40141 gcttattagc gatttgaatc agcttctggc gttcaaagct ggcatcgcgc tctgacagcg

40201 acttaactgc aaactggata gcccggtcag ctttaacctc cgggctggta aaatccgggg

40261 ccatgttgcg cgctatatca gcctccagag gtttaccgtg tccctgccat tcacggttat

40321 caaaatcaat gccgagcgtt ttggcgcggc tggcccattc ctggtgaatt tcttcacggg

40381 aatgctctgt tttcttttca cgcgtagcca tcgagacgcg gcttttcgtc tgagcatcgg

40441 cggtttcccg cgtcagcccc attgcagcga gtcccttttc aatttgctcc gaccggcggg

40501 aaaaagcgcg aatctgttca tctgaaaaat gggccatatc gaacgtgtta tttttgctgt

40561 tgtaacgcag ctcataaccg gctttggtca actccaacgc cagctcctgt ttgtaaacat

40621 cgcccaggtg cattttatta cgcatcagct catcattttt gagcgcgcgc cactggccgt

40681 cctcgcgctg ggtcatgttc atgacaaaag cgtgtgtgtg caaatcagga tctagcgccc

40741 tggacgtttc gtggcggaaa gtagcgacga caaggttatt ggtattctgg gttactgatt

40801 tcccctggcg agtcgtccgg gcctgcgcga gtttttcagc ttcacgcaca gcagcggcaa

40861 cagctttttc atgagcctcg ataatggttt tatcgccgtg tatcagcgcc tgcatggata

40921 cccctttagg cgctgaaaac gtcaggtcgt agcccagacg ctcttttttg gcatcaccca

40981 cgtgtcgctg catatgcgtg aaggtatcta tctcaccgac aagcagctct ttaaaccggg

41041 ctgattcaac gtccccggat aagccgaggg cttcagctcc ggttccctgc caggacgtga

41101 atgatgaatc cttactgtag taatcatcct ttgcatcaga gtagtagccc acaacgctag

41161 tgacgttctg gcgggtaatc gtggttatat caagcatcag atatccctca gttcaatgcc

41221 aggaacaggg tttttgcgat ggtatttaac gtgtttagcc ttgaacttag cgacgggcat

41281 atcaccaggc aacgccagat agccggtgag gtttggcaac attgatattt cggtaggcgt

41341 tacggcacga acaactttaa cgtcgcggcg tttacggaca atccagggct tctgaggatc

41401 ggattcttta cgctcaactt cgccttctat ctcaccgagt gagcgcgaca tttgatccaa

41461 cgtttcatca ccgagacggc tgccgcccag cacgatgtta gaacgcatgt tagccagaat

41521 tgtctgagcc atatcccgac cataaacctt aaccagctga gaataggttt gatagccagc

41581 ataaacacac agaccgcttt tacgcccttt ggtcagtgca tcgttgaggt ttggcagaaa

41641 ctggagtgat tccagctcgt caataaatac attaatgcgg ctttcttttt cacccatacc

41701 cagcacgata gaaaaaatcg aatccagcca gcaggaaatt agcggattaa gtgacctttt

41761 catttcttcc tgccaggtga taaacaaggt tcccggcttt ccatcatcaa gccagtcacg

41821 cagggaaaaa ttaccttccg gcattttcaa atgtggggca agattcttac tgagaacaaa

41881 tcgcgcgctt ccaactgctt tttcagaccc ggaaaaaata gcttcggcag gcgtccccat

41941 taaaaattct tttaattttt tctggtcaac gttacaggcc cagtgaataa cttcttccat

42001 agttactgtg ctgtataggc tgtgaagttt tttcgaaact tcactaaaaa taagacggcc

42061 atagccgaac cattcttcag tagccatatc agggctttcc tgaacaatag agttcactaa

42121 gcgctcgtaa tcatatgaac ggcgaatttc attgaaaaac acccagcctt cagtgcgttt

42181 atcataggcg tttaaaataa catcgccggg acgatagaaa ttctttaaga accccccatt

42241 tggatctaaa gcaatatttt tgccgcctct aatgatgctc ttaaataaca gttcattgaa

42301 aattgtggtt ttaccagtac cggttgtacc ggcaatcgaa aaatgcaagt tctcagcgta

42361 tgtaggtatg gggatattag ccacggttaa ctggttgaca cctctttcgc gtgttttatc

42421 agcgagtgtt ctggcgctaa caagctctgt accacgataa atctttttga atctttcgcc

42481 tttaaacacg cgtgatttat cataaatgat aaaagcgatc agaccgccaa caccaataaa

42541 ccagccagca attaaagctg accataaagg ccatagcgaa aaagtattct taaccagata

42601 cggaatcagg tatttagccg tggatggatc aataccgtag gtaaattttg caactagaaa

42661 ccataccatc actggaggca aagtaattgc aaataaaaat gctaagcctc tttctctatc

42721 gtccatttca gcgctccttt tttggttccc agactttgta gccgttacgt tcaacctctg

42781 cttttgccgc tttggttttg cccggttctg ctatcgagcg caggaggatt agcgtttcaa

42841 tagcgagtga ttcatgcaac atcatctgtc ctgccggtgg gaattttacg ccagatagcg

42901 tttcggttat tgccttcagc tcatcgcgca gtgggccaaa atccgcatct gaagcacggt

42961 caaaaagata atccagtttg cgatttacgt cgctcagccg gtcggcaact attttcaacc

43021 cggactcccg atcacctggg ccagcttcaa tgcagcgccg cagataatct gaccgattac

43081 ctcctgaaac caggtctata taggccaaaa gttcatctga tacttttgcg gttattattg

43141 gcattcagtc ctcacattgt gcatttttta aacaaaaaat tgggatctaa caagctgaaa

43201 tcttagtatt accaaagtaa taaagcaaac tcattataaa acaatgagtt attgggtgtt

43261 tttaatacct aattattacc gaatattgtt gctatttatt tttttatctt ttaaatcagt

43321 atgatagcgt gatttatcgc gctgcgttag gtgtatagca ggttaaggga taaaaaatca

43381 tcttttttgg taggggcgat ctacgtaggt taaggactaa ctggctaaaa agcgttcaat

43441 attccgtatt catgcttgca tgaataccag tacaacaaaa gtacatcaaa attacatcaa

43501 aattacatca cttgaaggtt gacagtacaa caaaattaca tcattctttg gtcatgaggt

43561 agccagtaca acaaaagtac atcaaaagta catcaaaatt acatcaaaat tacatcattc

43621 taaatgaggg tactatgaag cccaaaagta tcagggcggc acttcagttg atgttgccgg

43681 aaatagaaga aatgctgtca ctgggagttt ccagggagga aatttataag gcagtttctg

43741 aacgcttcgg cctggaaggt gtgaacgttc gtagctttga tacgtcccta tatagagcgc

43801 ggcaaatccg gaaaaatgga atgcacaata cacatgaaag gatgccgaac aatgatgata

43861 gtgtattgca caatacacaa aaaggaggta gcgagaaagg tgcagaggaa agtgtattgc

43921 acaatacaca aacgccgcct gagcctgaac cgcaaggaag tgaaaaaaaa gaaagtcccg

43981 gcattattga taaagagttc ttcaataaaa tcagtgaaga tttcgaccct aagatgttca

44041 ataaaaaatt ctgaggtgat ttatgaaagt agcggtaatt aattacagtg gcagcgttgg

44101 taaaacctta atttcatcct acctgttagc cccgcgtctg actggagcaa aattctatgc

44161 ggttgaaact atcaaccagt ctgcttccga tctggggatt gagaatgttt ccatttttaa

44221 aggtgatgac ttttcacggt tgattgaaga tattgttttt gaagatgccg ggattattga

44281 tattggtgcg tcaaacgtag aagcgttcct gatggcaatg tcccgctttg acagtggcgc

44341 gaatgaattt gataagtatg taatcccggt tacgcctgat aataaggcca ttgatgaaag

44401 cctgaaaaca gcacatacgt taagtaaggc aggtgtaagc agcgataaaa tcatctttgt

44461 tccaaaccgc attagtccag atagtgaagt agaggatgta ctggcgccgg tatttgaatt

44521 tgtcaaacga acgaaagttg gcaaaatcag caagaaatct gttatttata acagtgaagt

44581 tttcgaatat ctcgcgtatc accgtatctc attcgaagca ttgaccgctg aagatccaga

44641 agaatttaaa gcccgcgcta aacaaacaac ggatgctgac gagcgcaaaa aactggcccg

44701 ccgttatacc tacatgaaac aggcaattcc tgttaaagct aatctcgata aagcatatgc

44761 ggctttaatg ggagaataaa atggaaaagc agccagataa atttgaagtt ctgatggatt

44821 ggtttttagg tgacgcgaag gaaatcaccg caagtcagaa agaaatgact gagatacttt

44881 ctgcgctttc ggaaaagctg gcaaaagaca ccgaaagttt aggagagacg gcagactctc

44941 ttaaacggac tttagtagaa aaccagcgtt caattagcct ggcaattagt gatgatgcta

45001 aggcgcgtga ggaatttctg acgaagttcc gccgcgcgca ggtgtccaga gctgagacgt

45061 taacccgtca gatccttttt attacagctg gctgcactat tgtgggcgcc gccgtgggtg

45121 ccgcgatagc cataattcta ctgagataat gtaaaccggg catgtcccgg ttttttttca

45181 agcgaagcgc ggaggccgca ggccggaggc attagtggcc gccgcccgaa ggggcgagac

45241 gcgtagcggc tcgatgcgca gcacggcaga acggccccgc aggggtaatg cccggtttaa

45301 ttcaacgtga cagtcacgtg gaggaaaaat atgaatgacc gacagcgtga acaagcccgt

45361 attcgccagg cccggcgccg cgcgcgactc aaagaggaag gcgctagcgt gacagtcacg

45421 ctaacaaaac aggaagaagc aatgttacag gagctttgcc gggttcgtcg tccaggacga

45481 acagcctatt ccacgaatga gtttttccaa ctgctgctga tccgaaactg gcaacagtgg

45541 caggagcaaa aggcgcagct ggggaaatgc caggcttgcg gaaagctgaa agcggaggga

45601 ggttgcggcg gcgaacggca gagcgaaacc tttaactgct ggctagccgt cgaagcaaac

45661 gagcttaatg tgtagtgtat tgtgctatac agaaattgcc aaaagcagcg cggagtaata

45721 actgaaacgg ccgcgcagcg gaaagaaaaa agcccggtca atccgggttt ttttctttcg

45781 gcggctcagt aatcgtctag atctccgcag cccagcgggc aacaattacg ctcaataccg

45841 tggctgcaat aatcgttttc ccgatcctgc cggatttctt ccgcgatctc gtcttgggtc

45901 agatagtcaa attcacgccg cgctaccgcg atcagttctt cccgatactc ggccggaacg

45961 ccagcaagat agccgtcaat aaccgcgctc aggcggcgct gatacagttc ataaatcagg

46021 ccgcagcggg gctgctgttc gcgcgcaaaa ctctcacact ggcgacgaaa atcttcaaga

46081 gtctgctcaa gggtttgtgt ggtcatgtcg tttgtcctcg atctttctgt taaagacccg

46141 gcttggccgg gtcgtcagtg ctatttagtt tgttgcagca gctggtctaa ttttgccgcc

46201 agtgcatacg tcgatgtgaa agcgccttgc aggcgctgat taggcagctg gttgaacgct

46261 tcgaggcagg cgcgcagcaa taattctttc tgagattcga cttctttttt cagctgggaa

46321 ggggtggtaa cagtggtcat gcttactcct tagtgatccg ataccggcaa tttttcgggt

46381 ggcggtattg cctcccgatg atttaattat cggtgattac gcctttaaag tcaatacaag

46441 tacggaattt atttacatgt ttttatgccc gtcagggcat ggaaggcgac cgcgccggac

46501 tccaccggac accggccgca aatcgccgga aactgcggga ctgaccggag caacaggcca

46561 accccccttc ctgctaagcc ataacccagc ccgccgccac gcagctgccg cacgtccccc

46621 acgggggtgc gcagtgggcg ccgcgcgcct gcgcgcgggt acggcggccc gcctgcgggt

46681 cgcggcgccg tactgcgagt tagcggccgc cgcgcggccg gttacggggg acaccgcacc

46741 gtcacggcca gcgccccaca ccgcaccgtc acggccagcg ccccgctgag ctgcacaatc

46801 cacggataac acaatagcgc actggcaaag gatgccgacg cctgaagggc gttggcaccc

46861 cgaaggggcg gggcgagacg ggaaccggct cgatgcgcag cacagcagag cggccccgaa

46921 ggggtaacgc cctgtgtggc atcaggattt agcgcaatgg cagaacatga gctggagaga

46981 tcaccggcaa gcagcagcaa aggggcggcg cagccgccca gatggctgtt tgccgatacc

47041 ggcgattaat tagagcggtg tttaatatcc cccgcgtggc gggggactag gtttcagcaa

47101 gtcatgttaa atacgtgtcc gtcatgtaaa ctgaaatccc caataaacag atcccgcgca

47161 taggctacga tgtcaaaata tcgggctacg gattcaggaa tttcattcag tagaccgcta

47221 tcattcaggt attcctctgc aaaagtttct tcgtccttag cttcgcccat ataggcatct

47281 ctaaacaggt cgaaatcagt gctattaaac agatcaacaa aggccacaaa cgccgcttcg

47341 tttccttcct cgcgggcttg tttaaagccg ttaataaaat cccagttgat atggcactct

47401 gacgccatat cagacggaat accctcccaa tcctggaaca taaattctgg atcagcctca

47461 tttgcgtgta actcgcggca gcgctcgtaa aactcctctg agctatcaaa atcggtcaga

47521 tcgagccagg ctcccgcaat gcttccgcag ttgtatttat ggtaagtgcc aacataaaca

47581 gaaggggtcg taatatcagt catggtgtac tccttaaagc gccgataccg gcaatttttc

47641 gggcggcggt attgcctccc gatgatttaa ttatcggtga ttacgccttt aaagtcaata

47701 caagtacgga atttatttac atgtttttat gcccgtcagg gcatggaagg cgaccgcgcc

47761 ggactccacc ggacaccggc cgcaaatcgc cggaaactgc gggactgacc ggagcaacag

47821 gccaaccccc cttcctgcta agccataacc cagcccgccg ccacgcagct gccgcacgtc

47881 ccccacgggg gtgcgcagtg ggcgccgcgc gcctgcgcgc gggtacggcg gcccgcctgc

47941 gggtcgcggc gccgtactgc gagttagcgg ccgccgcgcg gccggttacg ggggacaccg

48001 caccgtcacg gccagcgccc cgctgagctg cacaatccac ggataacaca atagcgcact

48061 ggcaaaggat gccgacgcct gaagggcgtt ggcaccccga aggggcgggg cggccgcttg

48121 cggccgggcg agtccggcgc agggtgtggc ctgccaagcg gagcgcggag gccgaaggcc

48181 ggaggcgtta gcggccgctg cccgcgtaag cggggcgaga cgggaaccgg ctcgatgcgc

48241 agcacagcag agcggccccg aaggggtaac gccctgtgtg gcatcaggat ttagcacaat

48301 gtcagaacat aaactggaga gatcaccggc aagcagctgc aaaggggcgg cacagccgcc

48361 ccgatggctg ttacttgtct ttgtcgcgta gcactttgat taggccggtt acggccgtaa

48421 tcagagcggc cagcgaggtg atgatttgcg gtaggttttc gaggatggta gaggtcatat

48481 agcacctgta gagaagttgg cggggtgtcg tttccgacgg ccgcactgta accgggcgaa

48541 taaggcaggt tgtcaacagc ttgagcgaag cgtctgttga caacctgccg cgcccggttt

48601 cactgcggtc ataggcggaa cgacccacgc caacggaacg gctttatgac cgggcagctg

48661 agataccggc gaacctggct ggcggctgac gccagccgcc aagcgccagc gcggagggcg

48721 aagcccggag gccaagcgga gcgcggaggc cgaaggccgg aggcgttagc ggccgctgcc

48781 cgcgtaagcg gggcgagacg ggaaccggct cgatgcgcag cacagcagag cggccccgaa

48841 ggggtaacgc ccggagtctg ccgctgttta tctctcgttc catctgaaat cggcggtaag

48901 gccattaaaa gggtcagttt atcagggggg cgttagcccc ccatgttgtt aatcatcagg

48961 caatatcgtc tttgtagcag gcataaccga agctaagctc tgttttcata tagtggcggg

49021 caaagtccca ggcatcgtgg ccgaagtcct cataatctgc caggacgatt tcgcgggctt

49081 tgtgccattg ctgtacggaa gaaagcggca gaacagggca gggaagcgac cagtcagtga

49141 cggtgttgca gatcatatct gccagacgct ccagggagcc gtaaaccagc tctgtgcgca

49201 gatccgccac catgcgttgt ttacgcagtg atgccagata atcaatctct ttggttatat

49261 cagaatttaa gcgggtctgg taatccatga tgtactcctt tgcgcgccga taccggcaat

49321 tttgcgggcg acggtgttgc ctcccgatga tttaattatc ggtgattatg tcctcaaagt

49381 caatataagt acggaatgtg catactaaat tttatatctt gcaaagcgtt catagagtgc

49441 ctgaatcgct ttctgacagc ctcaataaaa aaggcgggga ttacccgcct tttttcttac

49501 agctgcttac gtggcttttt acgcgtcata tacaacggta tcgcgcagtc taccgcgtac

49561 aaaaagcacg ccagcgcgcc gcaaccgtac agaaacgcaa gcggcttatt atcgaagtag

49621 ctgaaaaccc ctgtcgccgc acacaggccc agaacagaga cgcaggcgca ggtgatctgc

49681 accagatccc tgtatcccgc acgaacgata aaccagggca gggcaagcgc agcggcgcta

49741 aaaattaatg cgagagggac aaaaacgaga tagtgataca tgtgaactcc ttgatggttg

49801 ccgataccgg cgattgttcg ggcggcggta ttgccacccg atgatttaat tagaggtttt

49861 gcgcgtccag gagattgacc tgagccgggg taacgtgaaa cttttcccct ttatggatca

49921 cgttatgcgg ggcgctaatt tcatcactga taaagctaac cgggtaacgt tttttaccgc

49981 aaatccgctc gctaaaccac gccacttttg ccgctggccg atccactgga tgaacaatca

50041 caccggccat gctgcaaccc gttgcgggtt cgtccagcgt aatgcttacc gggactgtat

50101 cccggtaaaa aaacttaacc ggcggcacac ctgcctgcgt agcggctgcg acaactgcaa

50161 gcccgataac cgctatccga ttaataagca ttttattccc cttactcatg ctgatatcac

50221 cttgccagct gttaccagtt tacgaaattc gctttcatga atttcacgcc catgctcaag

50281 cgttgaatac acgttgccga taagccagtt accagccgtt tttgtctcgg tataccacca

50341 tgcttctgtc agagtaagca gcggttcgct atcctcgccc ttctcataga tccagatttt

50401 agtgacgtct ttacccgttt cgttgctgcc ctgaatggta gggtcaaagg tatgttcaat

50461 ctctatatcg aagtaacgct gaaggaagtt agtaaagtgc atgacgactc ctgtaagcgc

50521 cgataccggc aatttttcgg gtggcggtgt tgcctcccga tgatttaatt atcggtgatt

50581 acgcctttaa agtcaataca agtacggaat ttatttacat gtttttatgc ccgtcagggc

50641 atggaaggcg accgcgccgg actccaccgg acaccggccg caaatcgccg gaaactgcgg

50701 gactgaccgg agcaacaggc caacccccct tcctgctaag ccataaccca gcccgccgcc

50761 acgcagctgc cgcacgtccc ccacgggggt gcgcagtggg cgccgcgcgc ctgcgcgcgg

50821 gtacggcggc ccgcctgcgg gtcgcggcgc cgtactgcga gttagcggcc gccgcgcggc

50881 cggttacggg ggacaccgca ccgtcacggc cagcgcccca ctgagctgca caatccacgg

50941 ataatgcagg agacgaatca tgataggagg ctgaagggga aatgagcggc agcaggggaa

51001 ggggttgcca agcggagcgc ggaggccgca ggccggaggc gtcagtggca gctgcccgcg

51061 tgagcggggc gagacgcgta gcggctcgat gcgcagcaca gcagaacggc cccggagggg

51121 tgacgtccgg gggttcgctt ttaaagattt tcgaccacat cagtaaatcg tagtgacacc

51181 atgaagcaaa agtatcgtgc agaccagaat caacaacagt aacagcagac ctttttttcg

51241 acgtaataaa accggcccca aagcgcccgc aagaagcaca ctcaacacga tttccgttat

51301 gctaatcgta ttcataatct ctcacgtttc cctttttaga actctgccac acacggataa

51361 aaccttataa caaagcaaca aaacccgtta ttcagacacg gtaatgccta gtttttttgc

51421 cagaatttca gatgacaaag aaacccttat cgacttcccg gttacaaggt gaatgattgt

51481 ggcttcatgc ccttcaacag agtgggatat taactcacgc tgaatgatat gcgtctctcc

51541 atcatttccc gtcacggcta ttaggctctc tgtagaacag gggttgatac tactaatgtt

51601 ggtgttgttt tcggttttgt tcagcatcgc tgatcctcaa atatcggttt gtgttacgtc

51661 tgccgctttg cgctggataa gcgacttaaa gaaatccgac gccttcagaa tatcgctatc

51721 ctggaagtcg ggaaatgtcg cttctgtcag ctcctgcgcc gtacctgccc gtataacggt

51781 ttcatcgcgg atcatcgttc gcttaacgcc atcagcctgt tttttgctga aggagtgccg

51841 gaaaataatc gcgtattggc cgccgctatc ctgatgaaca gaagtatccc agcgatgatt

51901 accctgttta cgatagtggt gatagatttg ctggccgtag cgctcctgat cggaattacg

51961 gtaatcgaac attaacagtt ccaaaagcat taaccgatct ggcaattgcc aggcggtagg

52021 gtgttgaagt gtcgctaaca tagtttcccc tgagcgtgac agtcacgata aggcgggctt

52081 tgcccgcctg gttatcagtt aatcaatggc acgataaata cgattctggc tttcgttctc

52141 cagcgtatta acgtactccc gcaacaggtg ataccggtta gccatcgttt cgttcagttc

52201 ggctttgcct tcttcatatg ccagcccgca aaagtaactg taggcataca gacaaacaat

52261 aatccctact tcgcgcgcgc tgcattcacc ttcaaaatag ttaggtaacg agagccaaag

52321 aggttggggc gcttccataa aaaacgcgcc attgctggcc tgaaggtatt cccaataccc

52381 tccctggtag tctttagcgt aacgattcag aaaggactga atgaagtgat ctgcgctgaa

52441 gaaagcgcca cgaaatgccg caggcatgaa gttcatgcgg gcgttttcag aaatgtagcg

52501 ggcggtgatt tcgatagttt ccatgatact tcctctttaa gccgataccg gcgatggtta

52561 agcggcaggc acatcacctg ccacttttta attatcgtac aatggggcgt taaagtcaat

52621 acaagtacgg attatattta cctaatttta tgcccgtcag agcatggaag gcgacctcgc

52681 cggactccac cggacaccgg gggcaaatcg ccggaaactg cgggactgac cggagcgaca

52741 ggccaccccc ctccctgcta gcccgccgcc acgcggccgg ttacagggga cactgagaaa

52801 gcagaaagcc aacaaacact atatatagcg ttcgttggca gctgaagcag cactacatat

52861 agtagagtac ctgtaaaact tgccaacctg accataacag cgatactgta taagtaaaca

52921 gtgatttgga agatcgctat gaaggtcgat atttttgaaa gctccggcgc cagccgggta

52981 cacagcatcc ctttttatct gcaaagaatt tctgcggggt tccccagccc ggcccagggc

53041 tatgaaaagc aggagttaaa cctgcatgag tattgtgttc gtcacccttc agcaacttac

53101 ttcctgcggg tttctggctc gtcaatggaa gatggccgca tccatgatgg tgacgtactg

53161 gttgtggatc gctcgctgac ggccagccac ggctcaatcg tagtcgcctg catccataat

53221 gaatttaccg tgaagcggct actgctgagg cccagaccct gcctgatgcc gatgaacaaa

53281 gattttcctg tgtactacat tgacccggat aatgagagcg ttgaaatctg gggagtggtt

53341 acgcattccc ttatcgagca tccggtatgt ttgcgctgat tgatgtcaat ggcatgtacg

53401 ccagctgtga gcaggcattt aggccagatc tggcaaaccg agcagtggcc gttttatcca

53461 acaatgacgg caacattgtg gcccgtaatt acctggcgaa gaaagcgggc ctgaaaatgg

53521 gcgatccgta cttcaaagtc agacccataa tcgagcgtca taacatcgct atttttagct

53581 ctaattacac tctctatgcc tccatgtcgg cccggttcgc ggccgtagtt gagtcccttg

53641 caagccacgt cgaacagtat tcaatcgacg agctttttgt tgactgcaaa gggataacgg

53701 ccgccatgag ccttgacgct ttcgggcgcc aactgcgcga ggaagtcagg cgacacacaa

53761 cgctggtatg cggggtcggt attgcccgta ctaagacgct ggcgaagctg tgtaaccacg

53821 ctgcaaaaac atggcccgct actggcgggg tggttgctct ggacgatggc gccagactga

53881 agaaattaat gagcatcctg ccggttgcgg aagtctgggg cgtcggccat cgtacagaga

53941 aagcactcgc cacaatgggg atcaaaacgg tgctggattt agccagggca gatacgcgcc

54001 taatccgtaa gacgtttggt gttgtgcttg aaagaacggt acgggagcta cgcggcgagg

54061 cttgcttcag cctggaagaa aaccctccgg cgaagcagca gattgttgta tcgcgctcat

54121 tcggccaacg cgtagtagcc ctggcggata tgcagcaggc gatcaccgga tttgcagcgc

54181 gcgcagctga aaaactgcgt aatgagcggc aatactgccg cgtcataagc gtctttatcc

54241 gcaccagtcc ttattcagtg cgtgatacac agtatgccaa tcaggcaacc gaaaaactga

54301 cggtggcaac ccaggacagc cgcacgataa ttcaggcggc acaagccgcg ctggcgcgga

54361 tctggcggga agatattgcg tatgcaaaag cagggatcat gctggccgat ttcagcggaa

54421 aggaggccca gctggattta ttcgactctg ctacgccttc agctggcagc gaggcgttaa

54481 tggctgttct cgatggcata aaccggcgtg gcaagagcca gctgtttttt gcaggccagg

54541 gcatcgataa ctcctttgcc atgcgccgtc agatgttgtc acctgattac acgacagact

54601 ggcgctcgat accaacagca accatcaaat aattaccggc gccgcacgcg ggccggtcaa

54661 cccctcaacc ggccgaaacg agtttcggcg cggttgaggg gttttcggta aaaggcgttt

54721 catctgtata aaagatcagc taaattatgt gtattgcaca atacatatat gtgaggttaa

54781 cagtgaattt gcctacgccc gaaacctacg atgaacttca gagagcctac gattttttta

54841 atgagaagct attcagcaac gagctgccgc catgcctgat aacgttgcag cgtgagaagc

54901 gaacgtatgg ctattgttcc tttaagcgtt tcgtcggccg tgagagtggg tacacggtag

54961 acgagatcgc tatgaatccg gtgtatttct cgatcagaac cataaaggcc acgctttcaa

55021 cactggtgca tgagatggtt catcagtggc aattccattt tggcgagcct ggccgccgtg

55081 gctatcacaa caaacagtgg gcggcccgga tggaacgggt aggactaatg ccttctgata

55141 ccggcgaacc gggaggcagg aaagtgggcc agagcatgac ccattatatt attgccggtg

55201 gccctttcga tatggcctgt gatgaactgc tgacaggcca tttccggctt tcctggatgg

55261 acaggtttcc gccttaccag cctaagcctg gcgctgtgct aagccctaca ggaaaaggct

55321 atattgacga cgaggaagat gatagcgaac acgaacagga ggtggaggaa gggcgcgacc

55381 cggttgaact cgacgacgag atcatagagg ccatgcgatt tgtaaccccg ccgcctgaag

55441 cgccggtgaa caaaacaaac cgggaaaagt acagctgccc ggtgtgtcat atcaatctct

55501 ggggtaaacc ggggatagtg gtttactgtg gtggcgagca ctgtaataaa gccgcgttag

55561 tagtcttaaa ataaagtcct ttcggacttt attttttttc catttccgag gtcgtgatgt

55621 tattaatgct gtacttcgcg gcttctttta aaacagtttc agcaaggctt gctggtatcc

55681 agacctgaac taattttaat ggttcgccgt tctcggcttt aagagtggtg ttctggtaca

55741 aatcccagat tcgcttaacg gtgctggaaa tgttttgctt ggaacggcct actcgcgtgg

55801 ctacgtctga tgatttctca cctttgacaa gcacggaata gccaatatct gttgtgatgt

55861 gtgcaaagga agccatttgc ggcagcagct gtttccattc tgtttctgaa attctgtttt

55921 tctgagccat ctgtggcgcc tccgtagttt tggttacaga aaggatatac tcagaataaa

55981 ctggggtcaa tacaagtacg atttttataa actttatttt atttgagggt gaggcccggt

56041 gcggcagcag cgcgggcctc gatggtgccg cgaaggtgct ggcgccatgc ttggattaaa

56101 acatgaaccg tgaagaactg cgaaacttgt tttcgcggtt ctgaggggtt gaccgagccg

56161 cgaagcggcg ctggtaagcg atgatatgca catatccaca ggcatatttt taaaaggtat

56221 tttatagatt ttttatcttt ttaaagtctt ttagagctat ataactcatt gatttaaaat

56281 cataaataag tgttatctct gggaatccgc ccaccttgtt atgggaattg gcccacctat

56341 ctatgggaaa caccccacct tactatggga attagcccac cttgttatgg gaattggccc

56401 accttagacg aaactgtaaa aaatgtattt acttgtttga actttgtggt agtgtggaga

56461 gtaattttta acccacaaag gcaaggctc

//
