# Supplementary material for: Closely Related NDM-1-Encoding Plasmids from Escherichia coli and Klebsiella pneumoniae in Taiwan
Source: PLoS One. 2014 Aug 21;9(8):e104899. doi: 10.1371/journal.pone.0104899 (PMC4140731; doi:10.1371/journal.pone.0104899)
Supplement: Sequence S2 — Complete nucleotide sequences and annotations of plasmids pLK78. (DOCX) [file pone.0104899.s002.docx]

LOCUS pLK78 56072 bp DNA circular BCT 09-FEB-2014

DEFINITION Klebsiella pneumoniae plasmid pLK78, whole genome shotgun sequence.

ACCESSION

VERSION

KEYWORDS WGS.

SOURCE Klebsiella pneumoniae

ORGANISM Klebsiella pneumoniae

Bacteria; Proteobacteria; Gammaproteobacteria; Enterobacteriales;

Enterobacteriaceae; Klebsiella.

REFERENCE 1 (bases 1 to 56072)

AUTHORS Chen,Y.-T., Lin,A.-C. and Siu,K.

TITLE Sequence of closely related NDM-1-encoding plasmids from

Escherichia coli and Klebsiella pneumoniae in Taiwan

JOURNAL Unpublished

REFERENCE 2 (bases 1 to 56072)

AUTHORS Chen,Y.-T., Lin,A.-C. and Siu,K.

TITLE Direct Submission

JOURNAL Submitted (09-FEB-2014) Institute of Molecular and Genomic

Medicine, National Health Research Institutes, 35, Keyan Road,

Zhunan Township, Miaoli County, Taiwan 350, Taiwan

COMMENT ##Assembly-Data-START##

Assembly Method :: Newbler v. 2.7

Sequencing Technology :: 454; Illumina

##Assembly-Data-END##

FEATURES Location/Qualifiers

source 1..56072

/organism="Klebsiella pneumoniae"

/mol_type="genomic DNA"

/db_xref="taxon:573"

/plasmid="pLK78"

/country="Taiwan"

CDS 1..720

/codon_start=1

/transl_table=11

/product="repA"

/translation="MDKLLNKKIKVKQSNELTEAAYYLSLKAKRVLWLCLMQTYFTAS

VSEDDDEMAVLGDSTFKVKVADYEQIFQVSRNQAIKDVKEGVFELSRSAVIFYPKEGS

FDCVARPWLTEAGSRSARGIWEIEFNHKLLRYIYGLTNQFTTYSLRDCGSLRNPRTIR

LYESLAQFKSSGLWVTTHAWLNDRFLLPESQQKNLAELKRSFLDPALKQINEKTPLLA

KYSIDDSGKFLFSIIDKQNPV"

CDS complement(2227..3240)

/codon_start=1

/transl_table=11

/product="integrase IntIPac"

/translation="MKTATAPLPPLRSVKVLDQLRERIRYLHYSLRTEQAYVHWVRAF

IRFHGVRHPATLGSSEVEAFLSWLANERKVSVSTHRQALAALLFFYGKVLCTDLPWLQ

EIGRPRPSRRLPVVLTPDEVVRILGFLEGEHRLFAQLLYGTGMRISEGLQLRVKDLDF

DHGTIIVREGKGSKDRALMLPESLAPSLREQLSRARAWWLKDQAEGRSGVALPDALER

KYPRAGHSWPWFWVFAQHTHSTDPRSGVVRRHHMYDQTFQRAFKRAVEQAGITKPATP

HTLRHSFATALLRSGYDIRTVQDLLGHSDVSTTMIYTHVLKVGGAGVRSPLDALPPLT

SER"

misc_feature 3321..3381

/note="attI"

CDS 4708..5241

/codon_start=1

/transl_table=11

/product="dihydrofolate reductase"

/translation="MRNRRATLSPFMAALTQRSIVKISLMAAKARNGVIGCGSDIPWN

AKGEQLLFKAITYNQWLLVGRKTFEAMGALPNRKYAVVSRSGSVATNDDVVVFPSIEA

AMRELKTLTNHVVVSGGGEIYKSLIAHADTLHISTIDSEPEGNVFFPEIPKEFNVVFE

QEFHSNINYRYQIWQRG"

CDS 5293..6267

/note="Streptomycin 3''-O-adenylyltransferase"

/codon_start=1

/transl_table=11

/product="aadA16"

/translation="MPLLSGVRCTKHIIAHSQTIRSSLLLLFLSVHNKPYTNWELDIM

SNAVPAEISVQLSQALNVIEHHLGSTLLAVHLYGSALDGGLKPCSDIDLLVTVTAQLD

ETVRQALFVDFLEVSASPGQSEALRALEVTIVVYGDVVPWRYPARRELQFGEWQRKDI

LAGIFEPATTDVDLAILLTKARQHSLALAGSAAEDFFNSVPESDLFKALADTLKLWNS

QPDWAGDERNVVLTLSRIWYSAATGKIAPKDVAANWVMERLPVQHQPVLLEAQQAYLG

QGMDCLASRADQLTAFIYFVKHEAASLLGSTPMMSNSSFKPTPLRGAA"

CDS 6725..7564

/codon_start=1

/transl_table=11

/product="dihydropteroate synthase"

/translation="MVTVFGILNLTEDSFFDESRRLDPAGAVTAAIEMLRVGSDVVDV

GPAASHPDARPVSPADEIRRIAPLLDALSDQMHRVSIDSFQPETQRYALKRGVGYLND

IQGFPDPALYPDIAEADCRLVVMHSAQRDGIATRTGHLRPEDALDEIVRFFEARVSAL

RRSGVAADRLILDPGMGFFLSPAPETSLHVLSNLQKLKSALGLPLLVSVSRKSFLGAT

VGLPVKDLGPASLAAELHAIGNGADYVRTHAPGDLRSAITFSETLAKFRSRDARDRGL

DHA"

CDS 7969..9510

/codon_start=1

/transl_table=11

/product="ISCR1 (orf513)"

/translation="MSLARNATASQSPTQTNGYERHQPDQTLLYQLVEQHYPAFKASL

EAQGQHLPRYIQQEFNDLLQCGRLEYGFMRVRCEDCHHERLVAFSCKRRGFCPSCGAR

RMAESAALLIDEVFPKEPIRQWVLSFPFQLRFLLARHPQLMGQVLSIVYRTLSTHLIK

KAGYTKASAQTGSVTLIQRFGSALNLNVHYHMLFLDGVYAEDDYGKQRFHRVKAPTYD

ELNTLAHTLSHRIARCMEKRGILERDAENTWLTLEEGEDDTLTQLHGASVTYRIAVGP

QQGRKVFTLQTLPGREDKADSSSRVANHAGFSLHAGVMAEAHQRDKLERLCRYISRPA

VSEKRLALTANGQVRYELKTPYRNGTTHVIFEPLDFIAKLAALVPKPRVNLTRFHGVF

APNSKHRVQVTPAKRGKKPDKSEGLDTNWRDKSPAERHRAMTWMQRLKRVFNIDIEVC

EHCGGHVKVIASIEDPKVIEQILKHLKQKTAKANAAKQRELPPERAPPLTPSLFDPSQ

SRLFD"

misc_feature 9721..9742

/note="oriIS, replication origin of ISCR"

CDS 9885..10097

/codon_start=1

/transl_table=11

/product="putative alpha-N-acetylgalactosaminidase"

/translation="MDWFVIHAFVEALKAKAPMPIDIYDALAWSAITPLSEQSIAEGN

RTLDFPDFTRGQWRTRKPIFALNDAY"

CDS complement(10108..10746)

/codon_start=1

/transl_table=11

/product="phosphoribosylanthranilate isomerase"

/translation="MPAKIKICGISTPEALDATIAARADYAGLVFYPASPRAVTSNVA

GALTSRAAGQIAMVGLFVDADDAVIADALVAAKLNALQLHGSESPERVAQLRARFGKP

VWKALPVASASDVARAAAYAGAADLILFDAKTPKGALPGGMGLAFDWSLLAGYRGALP

WGLAGGLNPTNVAEAIARTGAPLVDTSSGVESAPGVKDTDKITNFAFAVRLA"

CDS complement(10751..11011)

/codon_start=1

/transl_table=11

/product="bleomycin resistance protein"

/translation="MILQRGGLQLEFFPYPDLDPATSSFGCCLRLDDLDAMVALVNAA

GAEEKSTGWPRFKAPQLEASGLRIGYLIDPDCTLVRLIQNPD"

CDS complement(11120..11932)

/note="New Delhi metallo-beta-lactamase 1"

/codon_start=1

/transl_table=11

/product="beta-lactamase NDM-1"

/translation="MELPNIMHPVAKLSTALAAALMLSGCMPGEIRPTIGQQMETGDQ

RFGDLVFRQLAPNVWQHTSYLDMPGFGAVASNGLIVRDGGRVLVVDTAWTDDQTAQIL

NWIKQEINLPVALAVVTHAHQDKMGGMDALHAAGIATYANALSNQLAPQEGMVAAQHS

LTFAANGWVEPATAPNFGPLKVFYPGPGHTSDNITVGIDGTDIAFGGCLIKDSKAKSL

GNLGDADTEHYAASARAFGAAFPKASMIVMSHSAPDSRAAITHTARMADKLR"

mobile_element 12036..12052

/rpt_type=inverted

/mobile_element_type="insertion sequence:ISAba125"

CDS 12343..12849

/codon_start=1

/transl_table=11

/product="putative beta-lactamase"

/translation="MKQWERDLTLRGAIQVSAVPVFQQIAREVGEVRMQKYLKKFSYG

NQNISGGIDKFWLEGQLRISAVNQVEFLESLYLNKLSASKENQLIVKEALVTEAAPEY

LVHSKTGFSGVGTESNPGVAWWVGWVEKETEVYFFAFNMDIDNESKLPLRKSIPTKIM

ESEGIIGG"

CDS 12842..13657

/note="Streptomycin 3''-O-adenylyltransferase"

/codon_start=1

/transl_table=11

/product="aadA1"

/translation="MAKTKLNIMREVVIAEVSTQLSEVVGVIERHLEPTLLAVHLYGS

AVDGGLKPHSDIDLLVTVTVRLDETTRRALINDLLETSASPGESEILRAVEVTIVVHD

DIIPWRYPAKRELQFGEWQRNDILAGIFEPATIDIDLAILLTKAREHSVALVGPAAEE

LFDPVPEQDLFEALNETLTLWNSPPDWAGDERNVVLTLSRIWYSAVTGKIAPKDVAAD

WAMERLPAQYQPVILEARQAYLGQEEDRLASRADQLEEFVHYVKGEITKVVGK"

CDS 14122..14961

/codon_start=1

/transl_table=11

/product="dihydropteroate synthase"

/translation="MVTVFGILNLTEDSFFDESRRLDPAGAVTAAIEMLRVGSDVVDV

GPAASHPDARPVSPADEIRRIAPLLDALSDQMHRVSIDSFQPETQRYALKRGVGYLND

IQGFPDPALYPDIAEADCRLVVMHSAQRDGIATRTGHLRPEDALDEIVRFFEARVSAL

RRSGVAADRLILDPGMGFFLSPAPETSLHVLSNLQKLKSALGLPLLVSVSRKSFLGAT

VGLPVKDLGPASLAAELHAIGNGADYVRTHAPGDLRSAITFSETLAKFRSRDARDRGL

DHA"

mobile_element 16035..16049

/rpt_type=inverted

/mobile_element_type="insertion sequence:IS6100"

CDS 16096..16860

/codon_start=1

/transl_table=11

/product="transposase of IS6100"

/translation="MTDFKWRHFQGDVILWAVRWYCRYPISYRDLEEMLAERGISVDH

TTIYRWVQCYAPEMEKRLRWFWRRGFDPSWRLDETYVKVRGKWTYLYRAVDKRGDTID

FYLSPTRSAKAAKRFLGKALRGLKHWEKPATLNTDKAPSYGAAITELKREGKLDRETA

HRQVKYLNNVIEADHGKLKILIKPVRGFKSIPTAYATIKGFEVMRALRKGQARPWCLQ

PGIRGEVRLVERAFGIGPSALTEAMGMLNHHFAAAA"

mobile_element complement(16901..16914)

/rpt_type=inverted

/mobile_element_type="insertion sequence:IS6100"

CDS 17127..18335

/codon_start=1

/transl_table=11

/product="EcoRII"

/translation="MSVFHNWLLEIACENYFVYIKRLSANDTGATGGHQVGLYIPSGI

VEKLFPSINHTRELNPSVFLTAHVSSHDCPDSEARAIYYNSRHFGKTRNEKRITRWGR

GSPLQDPENTGALTLLAFKLDEQGGDCKEVNIWVCASTDEEDVIETAIGEVIPGALIS

GPAGQILGGLSLQQAPVNHKYILPEDWHLRFPSGSEIIQYAASHYVKNSLDPDEQLLD

RRRVEYDIFLLVEELHVLDIIRKGFGSVDEFIALANSVSNRRKSRAGKSLELHLEHLF

IEHGLRHFATQAITEGNKKPDFLFPSAGAYHDTEFPVENLRMLAVKTTCKDRWRQILN

EADKIHQVHLFTLQEGVSLAQYREMRESGVRLVVPSSLHKKYPEAVRAELMTLGAFIA

ELTGLYADIP"

CDS complement(18369..19802)

/codon_start=1

/transl_table=11

/product="EcoRIIM"

/translation="MSEFELLAQDLLEKAEAEEQLRQENDKKLLGQVLEIYDQKYVAE

LLRKVGKNEWSRETLNRWINGKCSPKTLTLAEEELLRKMLPEAPAHHPDYAFRFIDLF

AGIGGIRKGFETIGGQCVFTSEWNKEAVRTYKANWFNDAQEHTFNLDIREVTLSDKPE

VPENDAYAYINEHVPDHDVLLAGFPCQPFSLAGVSKKNSLGRAHGFECEAQGTLFFDV

ARIIRAKKPAIFVLENVKNLKSHDKGKTFKVIMDTLDELGYEVADAAEMGKNDPKVID

GKHFLPQHRERIVLVGFRRDLNIHQGFTLRDISRFYPEQRPSFGELLEPVVDSKYILT

PKLWEYLYNYAKKHAAKGNGFGFGLVNPENKESIARTLSARYHKDGSEILIDRGWDMA

TGETDFANEENQAHRPRRLTPRECARLMGFEKVDGRPFRIPVSDTQSYRQFGNSVVVP

VFEAVAKLLEPYILKAVNADSCKVERI"

CDS complement(20395..21186)

/codon_start=1

/transl_table=11

/product="mrr"

/translation="MAPATRPAPYFTGGFARISQAALRGRYTAVAKHQGGLTALFFTL

RKPTMTMIPFPTTENLILWACSAIALLAVVFFRRSVRHRRHKRKQQSARRVLERIKTL

PGFPQKINYLRKIDPFVFEELLLEGFEAHGFRTIRNKRYTGDGGIDGQVIIGKYRYLI

QAKRYRGHIALQHVQEFEKLLKRHNCRGLFCHTGKTGAGSKSVSIASERMEIISGQRL

IDLLTPGSSFTIATAPQTMMKRTAATLETSTIVKDAGKENRYHES"

CDS complement(21439..21753)

/codon_start=1

/transl_table=11

/product="kikA"

/translation="MKKLLIPLIAAGSLLYIPASHAEDPCKVIMCMAGKLTGDSGGSE

CNSAEAAFFNIVKKNKHGFLPNHTKDARKAFLNECPDNGEGGSNQSMISQIISKYGKV

RL"

CDS complement(21750..22094)

/codon_start=1

/transl_table=11

/product="hypothetical protein"

/translation="MGELIDFAERQKSRRKKKASSIPPIFRKFRVHAIRLLASIIKSG

SYSVAYIVKKITGKLIKFYTILTIFVFVVEYIAGDIGYKSIYNAALLLILLTVINILA

SVYLNKLLRTKQ"

CDS complement(22110..22415)

/codon_start=1

/transl_table=11

/product="korB"

/translation="MLVPLKSEKRPKGEPVYRDPDNPFNTWTGIGKRPAWLTAKLDAG

ISLEAMKMQGVANPREHRPAKYRDPRNAENTWSGTGRRPTWLKELLDSGLSLDDLKI"

CDS 22524..23258

/codon_start=1

/transl_table=11

/product="traL"

/translation="MSKHPKLLVLALACLACAGRASAAPASDEVARLAQRCAPDVSPL

TMAYIVGHESSNGPYRININGSIQLKQQPRTEAEAVSVAKVLLKDNKSFDMGLAQINS

NNLVGLGLSVDDIFKPCINLRASQTILKACYDSALKSYPAGQVALRHALSCYNTGSLT

NGISNGYVTKVINVARQSTDLKIPTLLPDGQTSEDSTATEPQQAKSTAPQYDGEQDVF

GSGDGDAFSRNNTDAFLTRQETAKGE"

CDS 23255..23548

/codon_start=1

/transl_table=11

/product="korA"

/translation="MRLWMERLYLVLQSQILELFEAGKVKEVTIERVSLKKWYPVFQI

DDEQLGQIACSIRVNKEHELRTWADLRLLAEFLKDKCGVEECRLNLQSTEDSE"

CDS 23558..23851

/codon_start=1

/transl_table=11

/product="traM"

/translation="MTTLFKKYGPAVVMGVLSIALPQIALAAGTDTGESTATSIQTWL

STWIPIGCAIAIMVSCFMWMLHVIPASFIPRIVISLIGIGSASFLVSLTGVGS"

CDS 23901..24218

/codon_start=1

/transl_table=11

/product="traA"

/translation="MFVDGKRPLFKGATRLPRALGVPRNVAMMIFMISASLFMIIHMW

AILVFVFLWIPSAALTKYDDRMFRIMGLWLKTKFSNWFDSPFKQWGGSSYSSVDYKRK

GLK"

CDS 24218..26818

/codon_start=1

/transl_table=11

/product="traB"

/translation="MRAATATKPKKIDAYRKEPSVNKKYLPYSYHLNDYVISMENGDL

MAFFKLDGRTHDCASDRELVTWHKDLNTLVKSFGTDHVELWTHEYHHEAKEYPDGEYD

HFFPAYVDQYNRKLHGDSKQLINDLYLTVIYKQVGDKTQKFLAKFEKPTRDEIQQMQN

EALEGLEDISEQILEAMKPYGIQQLGIYYRDKRGVEIPAPDKKEREELAEVDESDIFD

EAIVIERNEPEPSQAHAYSKALEFLYFLANMEWAIVPVCRDRIREYIMDNRPVSSLWG

DVVQIRTVDHNFYTTGIEFREYEEDTEPGQLNMLKEADFEYLLTQSFSCLSESSAKTF

LTHQEKSLQETRDRAQSQLAQLGTALDMLTSREFVMGYHHGTVHVWDNDQNAVQRKAR

RVKVMLTGCGVVGGTISLASEAAYYARLPGNQKWAPRPVPINSWNFLHFSPFHNFMRG

KPDNNPWGPALTMFRTISGTPLYFNFHVTPLEELSYGKRPLGHALITGMSGEGKTTLL

NFLLAQSMKYNPRLFVYDRDRGMEPFIRSVGGYYKVLQQGMPSGFAPLQIEPTKRNIA

LIKNLFRICVETTNNGPISATMATELAEGVDAVMGEGSLIPREARTVTILDGYVNEVV

ENGVSLKGLLREWTREGQYGWLFDNDKDSLDLSANDIFGFDLSEFIAAKEEVSSPART

PLMMYLLYRVRDSIDGKRRVIQCFDEFHAYLDDPVIEREVKRGIKTDRKKDAIYVFAT

QEPNDALSSRIGRTIMSQTVTKICLRDPEAIREDYAFLTDAEYDALMSITEHSRQFLV

KQGQQSAIASFNLYPRNSDDIDADIKTMDNVLSVLSGEPQNAEIAHELVERLGNDPEV

WLKEYWRLTA"

CDS 26836..27549

/codon_start=1

/transl_table=11

/product="traC"

/translation="MKKTLTAVLLTTGLILGGAQSASAGIIVTNPTELAKQVEQLQQM

AQQLEQLKSQLQTQKNMYESMAKTTNLGDLLGTSTSTLANNLPDNWKEIYSDAMNSSS

SVTPSVNSMMGQFNAEVDDMTPSEAITYMNKKLAEKGAYDRVMAEKAYNNQMQELTDM

QELTEQIKTTPDLKSIADLQARIQTSQGAIQGEQAKLNLMNMLQQSQDKLLRAQKERA

THNFVFGTGGDVTASPSIN"

CDS 27557..27784

/note="entry exclusion protein"

/codon_start=1

/transl_table=11

/product="eex"

/translation="MKKLLLVIPFLLVACDASHDVEWYKKHEKERKATIQECKKDADE

LQKPDCKNAREADRQLFVFGKKDGEINSPKI"

CDS 27857..28840

/codon_start=1

/transl_table=11

/product="traD"

/translation="MVSANVATIISDVTPLIATCLTIKLMVQGMYSAFNPGAGDSLSS

LIKEYLSIALILSFATAGGWYQQELVNVALHLPDDFAGILSAPNKVGASGVPAIIDSG

IEKGIKIVNTAWEAADVFSSSGLAAYAIGGIMMIATVVLGGLGAGFVIMAKILLAVTL

CFGPIAIFCLLWGATKNIFARWLASVINYGLVVVILALVFGFIMQMFDNLLSSMNSDA

AYSSITGSISALLLTVISVFVLFQIPQIAASWGSGISAGVADAARSTGSSMQALGNMG

SHGMFGGNAFRGGNSGGGQQSAGGGSGSNSGGSSGSNLSGKARGSRGKKAA"

CDS 29059..29757

/codon_start=1

/transl_table=11

/product="traE"

/translation="MKANKKTGLTREAIKEFNESRKGLEVDLMDEVLKSRRTAWMVAT

GSAVVTVFALSLVGYVVHKYSQPIPAHLLTLNEATHEVQQVKLTRDQTSYGDEIDKFW

LTQYVIHRESYDFYSVQVDYTAVGLMSTPNVAESYQSKFKGRNGLDKVLGDSETTRVK

INSVILDKPHGVATIRFTTVRRVRSNPVDDQPQRWIAIMGYEYKSLAMNAEQRYVNPL

GFRVTSYRVNPEVN"

CDS 29768..30652

/codon_start=1

/transl_table=11

/product="traO"

/translation="MKKLLLSAVVLSVLGGAATNVMALEVGRNSPYDYRIKSVVYNPV

NVVKIDAIAGVATHIVVAPDETYITHAFGDSESWTFAHKMNHFFVKPKQAMSDTNLVI

VTDKRTYNIVLHFIGEETKKNADGTVSKSFIETPWAVRQAVLQLTYEYPFEQQEKAKS

AADKKRITQKLKQTAFAGAKNYQYVMSEQPEMRSIQPVHVWDNYRFTRFEFPANAELP

QVYMISASGKETLPNSHVVGENRNIIEVETVAKEWRIRLGDKVVGVRNNNFAPGAGAV

ATGTASPDVRRVQIGEDN"

CDS 30652..31812

/codon_start=1

/transl_table=11

/product="traF"

/translation="MARKSVDVDQELDENTGDGEFESERGGFKGSNRRSAPGMKAFVI

LMALLALVFIGITVMGKIRTPAKAEADKDGGKAQQANTLPNYSFNSDPDVNKPATAQN

SATDARAVQAAAQADADAGSSNTAARTSNKRKEPSPEELAMQRRLGGELAQTNQAATS

NSPGVQPQDNETSEGSSALAKNLTPARLKASRAGVMANPSLTVPKGKMIPCGTGTELD

TTVPGQVSCRVSQDVYSADGLVRLIDKGSWVDGQITGGIKDGQARVFVLWERIRNDQD

GTIVNIDSAGTNSLGSAGIPGQVDTHMWERLRGAIMISLFSDTLTALVNQTQSNNIQY

NSTENSGEQLASEALRSYMSIPPTLYDQQGDAVSIFVARDLDFSGVYTLADN"

CDS 31854..32849

/codon_start=1

/transl_table=11

/product="traG"

/translation="MTDAAFYQLGPLREYLEDPTVFEIRINCFQEVICDTFSGRRVVQ

NAAITADFIRNLAKSLVSSNKLTMQAINDVILPGGIRGVICLPPAVIDGTTAVAFRKD

LAADKNLEQLTSEGIFSDCRKITGSKQSLTDDDFFLKELHSSEKWPAFLQTAVEKKRT

IVICGETGSGKTVLTRALLKSLHKDERVIILEDVHEVTVDHVVEAVYMMYGDAGKIGR

VSATDALRACMRLTPGRIIMTELRDDAAWDYLKALNTGHPGGVMSTHANSARDAFNRI

GLLIKATPIGRMLDMSDIMRMLYSTIDVVVHMEKRKIKEIYFDPEYKMQCVNGSL"

CDS 32849..33382

/note="plasmid conjugative transfer endonuclease"

/codon_start=1

/transl_table=11

/product="nuc"

/translation="MKNLATWLLAAAFTTAALPAFAVEPSVQVGYSPEGSARVLVLSA

IDSAKTSIRMMAYSFTAPDIMKALVAAKKRGVDVKIVIDERGNTGRASIAAMNYIANS

GIPLRTDSDFPIQHDKVIIVDNVTVETGSFNFTKAAETKNSENAVVIWNMPKLAESFL

EHWQDRWNRGRDYRSSY"

CDS 34319..34561

/codon_start=1

/transl_table=11

/product="relaxase/helicase"

/translation="MKTAALDLARERQAHEAGARTRATAHERTPQQERQKAAREAERG

REAWTLGQGMKKPVAGCYGRLTRWKGGGDVVYMALL"

CDS complement(34593..35270)

/note="putative transcriptional regulator, TetR family"

/codon_start=1

/transl_table=11

/product="putative transcriptional regulator"

/translation="MFISDKVSSMTKLQPNTVIRAALDLLNEVGVDGLTTRKLAERLG

VQQPALYWHFRNKRALLDALAEAMLAENHTHSVPRADDDWRSFLIGNARSFRQALLAY

RDGARIHAGTRPGAPQMETADAQLRFLCEAGFSAGDAVNALMTISYFTVGAVLEEQAG

DSDAGERGGTVEQAPLSPLLRAAIDAFDEAGPDAAFEQGLAVIVDGLAKRRLVVRNVE

GPRKGDD"

CDS 35349..36548

/codon_start=1

/transl_table=11

/product="tetracycline efflux protein TetA"

/translation="MKPNRPLIVILSTVALDAVGIGLIMPVLPGLLRDLVHSNDVTAH

YGILLALYALVQFACAPVLGALSDRFGRRPILLVSLAGATVDYAIMATVPFLWVLYIG

RIVAGITGATGAVAGAYIADITDGDERARHFGFMSACFGFGMVAGPVLGGLMGGFSPH

APFFAAAALNGLNFLTGCFLLPESHKGERRPLRREALNPLASFRWARGMTVVAALMAV

FFIMQLVGQVPAALWVIFGEDRFHWDATTIGISLAAFGILHSLAQAMITAPVAARLGE

RRALMLGMIADGTGYILLAFATRGWMAFPIMVLLASGGIGMPALQAMLSRQVDEERQG

QLQGSLAALTSLTSIVGPLLFTAIYAASITTWNGWAWIAGAALYLLCLPALRRGLWSG

AGQRADR"

CDS complement(36580..37278)

/note="permease of the drug/metabolite transporter (DMT)

superfamily"

/codon_start=1

/transl_table=11

/product="putative permease"

/translation="MAWPAAVLPCFISRFLYRPLSVYRLPGGVAATVGAVQPLMVVFI

SAALLGSPIRLMAVLGAICGTAGVALLVLTPNAALDPVGVAAGLAGAVSMAFGTVLTR

KWQPPVPLLTFTAWQLAAGGLLLVPVALVFDPPIPMPTGTNVLGLAWLGLIGAGLTYF

LWFRGISRLEPTVVSLLGFLSPGTAVLLGWLFLDQTLSALQIIGVLLVIGSIWLGQRS

NRTPRARIACRKSP"

CDS complement(37428..37601)

/codon_start=1

/transl_table=11

/product="hypothetical protein"

/translation="MEQTDKRKQDKLKFDRVINLARRLPQPAIHDLLRALILPIQADY

LLGSSQNSENKAR"

CDS complement(37601..40837)

/codon_start=1

/transl_table=11

/product="traI"

/translation="MLDITTITRQNVTSVVGYYSDAKDDYYSKDSSFTSWQGTGAEAL

GLSGDVESARFKELLVGEIDTFTHMQRHVGDAKKERLGYDLTFSAPKGVSMQALIHGD

KTIIEAHEKAVAAAVREAEKLAQARTTRQGKSVTQNTNNLVVATFRHETSRALDPDLH

THAFVMNMTQREDGQWRALKNDELMRNKMHLGDVYKQELALELTKAGYELRYNSKNNT

FDMAHFSDEQIRAFSRRSEQIEKGLAAMGLTRETADAQTKSRVSMATREKKTEHSREE

IHQEWASRAKTLGIDFDNREWQGHGKPLEADIARNMAPDFTSPEVKADRAIQFAVKSL

SERDASFERQKLIQIANKQVLGHATIADVEKAYLKAVQKGAIIEGEARYQSTLKVGAS

VMAETLTRKEWIDSLTNSGMRADKARFAVDDGIKNGRLKKTSHRVTTVEGIRLERSIL

TIESRGRGQMPRQLTAEIAGQLLAGKTLKKEQMRAVTEIVTSKDRFVAAHGYAGTGKS

YMTMAAKELLESQGLKVTALAPYGTQKKALEDDGLPARTVAAFLKAKDKKLDEKSVVF

IDEAGVIPARQMKQLMEVIEKHNARAVFLGDTSQTKAVEAGKPFEQLIKAGMQTSYMK

DIQRQKNEVLLEAVKYAAEGNAARALKNITGVNELKEEAPRLSQLADRYLSLSSEQQD

ATLIISGTNASRKTLNDYIRGNLGLAGTGETFTLLDRVDSTQAERRDSRYFSKGQIII

PEQDYKNGMKRGESYQVLDTGPGNKLTVESSSGEQIAFSPRTHTKLSVYQAVSAELAP

GDKVMVTRNDKTLDVANGDRFTVKTVEGEKLTLEDKKGRTVELDKKQASYLSYAYATT

VHKSQGLTCDRVLFNIDTKSLTTSKDVFYVGISRARHEVEIFTDDKKSLASSVSRDSP

KTTAAEIDRFFGLEARFKDIGRDTSLETRSAEKGLPEATGESMAFNQKPDEHNMTTGT

DYQPVSNAEDAFHLKQNPMDDSVGLRRHEAQQNDAELAHDYAAADDQQWSAQEYADYE

HYAEASDYDFDSSIYDDYAMPQTSQAEQSHTGKEHTHEHEHEEGGHEI"

CDS complement(40837..42366)

/codon_start=1

/transl_table=11

/product="traJ"

/translation="MDDRERGLAFLFAITLPPVMVWFLVAKFTYGIDPSTAKYLIPYL

VKNTFSLWPLWSALIAGWFIGVGGLIAFIIYDKSRVFKGERFKKIYRGTELVSARTLA

DKTRERGVNQLTVANIPIPTYAENLHFSIAGTTGTGKTTIFNELLFKSIIRGGKNIAL

DPNGGFLKNFYRPGDVILNAYDKRTEGWVFFNEIRRSYDYERLVNSIVQESPDMATEE

WFGYGRLIFSEVSKKLHSLYSTVTMEEVIHWACNVDQKKLKEFLMGTPAEAIFSGSEK

AVGSARFVLSKNLAPHLKMPEGNFSLRDWLDDGKPGTLFITWQEEMKRSLNPLISCWL

DSIFSIVLGMGEKESRINVFIDELESLQFLPNLNDALTKGRKSGLCVYAGYQTYSQLV

KVYGRDMAQTILANMRSNIVLGGSRLGDETLDQMSRSLGEIEGEVERKESDPQKPWIV

RKRRDVKVVRAVTPTEISMLPNLTGYLALPGDMPVAKFKAKHVKYHRKNPVPGIELRD

I"

CDS complement(42368..42784)

/codon_start=1

/transl_table=11

/product="traK"

/translation="MPIITAKVSDELLAYIDLVSGGNRSDYLRRCIEAGPGDRESGLK

IVADRLSDVNRKLDYLFDRASDADFGPLRDELKAITETLSGVKFPPAGQMMLHESLAI

ETLILLRSIAEPGKTKAAKAEVERNGYKVWEPKKER"

misc_feature 43103..44822

/note="oriT region"

CDS 43275..43694

/codon_start=1

/transl_table=11

/product="stbA"

/translation="MKPKSIRAALQLMLPEIEEMLSLGVSREEIYKAVSERFGLEGVN

VRSFDTSLYRARQIRKNGMHNTHERMPNNDDSVLHNTQKGGSEKGAEESVLHNTQTPP

EPEPQGSEKKESPGIIDKEFFNKISEDFDPKMFNKKF"

CDS 43703..44419

/codon_start=1

/transl_table=11

/product="stability protein StdB"

/translation="MKVAVINYSGSVGKTLISSYLLAPRLTGAKFYAVETINQSASDL

GIENVSIFKGDDFSRLIEDIVFEDAGIIDIGASNVEAFLMAMSRFDSGANEFDKYVIP

VTPDNKAIDESLKTAHTLSKAGVSSDKIIFVPNRISPDSEVEDVLAPVFEFVKRTKVG

KISKKSVIYNSEVFEYLAYHRISFEALTAEDPEEFKARAKQTTDADERKKLARRYTYM

KQAIPVKANLDKAYAALMGE"

CDS 44421..44789

/codon_start=1

/transl_table=11

/product="stbC"

/translation="MEKQPDKFEVLMDWFLGDAKEITASQKEMTEILSALSEKLAKDT

ESLGETADSLKRTLVENQRSISLAISDDAKAREEFLTKFRRAQVSRAETLTRQILFIT

AGCTIVGAAVGAAIAIILLR"

CDS 44971..45315

/codon_start=1

/transl_table=11

/product="orfD"

/translation="MNDRQREQARIRQARRRARLKEEGASVTVTLTKQEEAMLQELCR

VRRPGRTAYSTNEFFQLLLIRNWQQWQEQKAQLGKCQACGKLKAEGGCGGERQSETFN

CWLAVEANELNV"

CDS complement(45426..45746)

/codon_start=1

/transl_table=11

/product="CcgAII protein"

/translation="MTTQTLEQTLEDFRRQCESFAREQQPRCGLIYELYQRRLSAVID

GYLAGVPAEYREELIAVARREFDYLTQDEIAEEIRQDRENDYCSHGIERNCCPLGCGD

LDDY"

CDS complement(45801..45980)

/codon_start=1

/transl_table=11

/product="CcgAI"

/translation="MTTVTTPSQLKKEVESQKELLLRACLEAFNQLPNQRLQGAFTST

YALAAKLDQLLQQTK"

CDS 46499..46612

/codon_start=1

/transl_table=11

/product="hypothetical protein"

/translation="MASGFSAMAEHELERSPASSSKGAAQPPRWLFADTGD"

CDS complement(46657..47166)

/codon_start=1

/transl_table=11

/product="antirestriction protein ArdA"

/translation="MTDITTPSVYVGTYHKYNCGSIAGAWLDLTDFDSSEEFYERCRE

LHANEADPEFMFQDWEGIPSDMASECHINWDFINGFKQAREEGNEAAFVAFVDLFNST

DFDLFRDAYMGEAKDEETFAEEYLNDSGLLNEIPESVARYFDIVAYARDLFIGDFSLH

DGHVFNMTC"

CDS complement(48519..48851)

/codon_start=1

/transl_table=11

/product="hypothetical protein"

/translation="MDYQTRLNSDITKEIDYLASLRKQRMVADLRTELVYGSLERLAD

MICNTVTDWSLPCPVLPLSSVQQWHKAREIVLADYEDFGHDAWDFARHYMKTELSFGY

ACYKDDIA"

CDS complement(49060..49344)

/codon_start=1

/transl_table=11

/product="hypothetical protein"

/translation="MYHYLVFVPLALIFSAAALALPWFIVRAGYRDLVQITCACVSVL

GLCAATGVFSYFDNKPLAFLYGCGALACFLYAVDCAIPLYMTRKKPRKQL"

CDS complement(49413..49613)

/codon_start=1

/transl_table=11

/product="hypothetical protein"

/translation="MAGVIVHPVDRPAAKVAWFSERICGKKRYPVSFISDEISAPHNV

IHKGEKFHVTPAQVNLLDAQNL"

CDS complement(49769..50065)

/codon_start=1

/transl_table=11

/product="hypothetical protein"

/translation="MHFTNFLQRYFDIEIEHTFDPTIQGSNETGKDVTKIWIYEKGED

SEPLLTLTEAWWYTETKTAGNWLIGNVYSTLEHGREIHESEFRKLVTAGKVISA"

CDS complement(51220..51555)

/codon_start=1

/transl_table=11

/product="hypothetical protein"

/translation="MFDYRNSDQERYGQQIYHHYRKQGNHRWDTSVHQDSGGQYAIIF

RHSFSKKQADGVKRTMIRDETVIRAGTAQELTEATFPDFQDSDILKASDFFKSLIQRK

AADVTQTDI"

CDS complement(51682..52107)

/codon_start=1

/transl_table=11

/product="antirestriction protein KlcA"

/translation="METIEITARYISENARMNFMPAAFRGAFFSADHFIQSFLNRYAK

DYQGGYWEYLQASNGAFFMEAPQPLWLSLPNYFEGECSAREVGIIVCLYAYSYFCGLA

YEEGKAELNETMANRYHLLREYVNTLENESQNRIYRAID"

CDS 52522..52962

/codon_start=1

/transl_table=11

/product="error-prone repair protein UmuD"

/translation="MKVDIFESSGASRVHSIPFYLQRISAGFPSPAQGYEKQELNLHE

YCVRHPSATYFLRVSGSSMEDGRIHDGDVLVVDRSLTASHGSIVVACIHNEFTVKRLL

LRPRPCLMPMNKDFPVYYIDPDNESVEIWGVVTHSLIEHPVCLR"

CDS 52977..54215

/note="error-prone, lesion bypass DNA polymerase UmuC"

/codon_start=1

/transl_table=11

/product="DNA polymerase UmuC"

/translation="MYASCEQAFRPDLANRAVAVLSNNDGNIVARNYLAKKAGLKMGD

PYFKVRPIIERHNIAIFSSNYTLYASMSARFAAVVESLASHVEQYSIDELFVDCKGIT

AAMSLDAFGRQLREEVRRHTTLVCGVGIARTKTLAKLCNHAAKTWPATGGVVALDDGA

RLKKLMSILPVAEVWGVGHRTEKALATMGIKTVLDLARADTRLIRKTFGVVLERTVRE

LRGEACFSLEENPPAKQQIVVSRSFGQRVVALADMQQAITGFAARAAEKLRNERQYCR

VISVFIRTSPYSVRDTQYANQATEKLTVATQDSRTIIQAAQAALARIWREDIAYAKAG

IMLADFSGKEAQLDLFDSATPSAGSEALMAVLDGINRRGKSQLFFAGQGIDNSFAMRR

QMLSPDYTTDWRSIPTATIK"

CDS 54366..55157

/codon_start=1

/transl_table=11

/product="zinc metalloproteinase Mpr protein"

/translation="MNLPTPETYDELQRAYDFFNEKLFSNELPPCLITLQREKRTYGY

CSFKRFVGRESGYTVDEIAMNPVYFSIRTIKATLSTLVHEMVHQWQFHFGEPGRRGYH

NKQWAARMERVGLMPSDTGEPGGRKVGQSMTHYIIAGGPFDMACDELLTGHFRLSWMD

RFPPYQPKPGAVLSPTGKGYIDDEEDDSEHEQEVEEGRDPVELDDEIIEAMRFVTPPP

EAPVNKTNREKYSCPVCHINLWGKPGIVVYCGGEHCNKAALVVLK"

CDS complement(55172..55513)

/codon_start=1

/transl_table=11

/product="hypothetical protein"

/translation="MAQKNRISETEWKQLLPQMASFAHITTDIGYSVLVKGEKSSDVA

TRVGRSKQNISSTVKRIWDLYQNTTLKAENGEPLKLVQVWIPASLAETVLKEAAKYSI

NNITTSEMEKK"

BASE COUNT 13411 a 14498 c 14874 g 13289 t

ORIGIN

1 atggataagt tgctgaacaa aaagataaaa gttaagcagt ctaacgagct taccgaagct

61 gcttactacc tctcgctaaa agcaaagcgc gttctctggt tatgtcttat gcagacgtat

121 ttcacagctt cagtaagcga agatgatgat gagatggctg tactcggtga ctctactttc

181 aaagtaaagg tggctgacta tgagcaaatt tttcaggtaa gccgtaacca ggctatcaag

241 gatgttaaag aaggcgtgtt tgagttaagc cgttctgcgg taatctttta cccgaaagaa

301 gggagttttg actgcgtcgc gcgcccctgg ctaacagagg ctggcagccg atcagctcgt

361 ggtatctggg aaatcgaatt taaccataaa ctcctgcggt acatttacgg cctgacgaac

421 cagttcacca cctactcgct ccgcgattgt ggcagtcttc gaaatccacg gacgatccgc

481 ctttatgaaa gtcttgctca attcaaatct tcaggcttat gggttactac tcatgcttgg

541 ttaaatgacc gtttcctttt gccggaatcc caacagaaga acttggcaga gttgaaacga

601 tctttccttg atcctgcact caagcagata aatgagaaaa cacctttact tgctaagtat

661 agtattgatg attcaggaaa atttctgttc tcaataattg ataagcaaaa tcccgtctga

721 cataaatcag cacacatgag cctgtcattt gacaaatttt tgtcatgaag gtgggcggat

781 ttccacacag caccggcgcc cggcaaggtg ggcggattcc cacacggcac cggcgcccgg

841 caaggtgggc ggatttccac acggcaccgg cgcccggcaa ggtgggcgga ttcccatatc

901 gacatgtatg tagcttgtgt tatccgtgga ttgtgcagct cagcgggtcg ctggtcgtat

961 ggcgtagtgt cccccgtaac cggccgcgtg cggccgctaa ctcgcagtac ggcgccgcga

1021 cccgaaggcg ggccgccgtt cccgcgcgca ggcgcgcggc gcccactgcg cacccccgtg

1081 ggggacatac ggcagctgtg tggcggtgag cgggattagg gctttgcagg gagggggctg

1141 ggtcgggcga tacgttcagc attgcggttt ccggcgattt gcggccggtg cccgtttaac

1201 tccggcgtgg tcgccttcca tgccctgacg gcataagaaa ataaaaccgc catgctgcgg

1261 tcattcatga ttttgtggtg tagcgataaa tagtcatgcg agaaacgttg aagcgcttag

1321 caactgcacc aactgtcatt tcaggatcag caagtaagat tctaatttgt ttaacatctt

1381 cttcagaaag tgacggtttt ctccctccca cacggcccct tgcgcgtgca gctgcaaggc

1441 ctgagcgcgt tctttcaata ttgcggttgc gttcaaagct agagaatatc gccatcagat

1501 gagtatagat ttcccctata actggcgcat ttgtgtctat tctgtccttg atggctatga

1561 aagttattcc gcgtttcttc aggtcgtcga gtaaagtaat gacttgaccc aatgaaccgc

1621 cgagccgatc tagtgcccaa actactaggg tatctccctc gcgcaatgct ttcaggcagt

1681 tctccagttc cggcgcacct tttttgtcgc gctttgggcc gctacgtgag gtctgatcct

1741 gatagatttg ctcacatcca gcttttgtta gttcgtcaac ctggtgcgcc acatcctgaa

1801 gatgcgtaga tttacgtgca tagccgattt tcattctttt ctcgctaatt agttatgggg

1861 ttattgttat gttgatacag taacgagttt tgttacatga ggggagtcat ttttcgggag

1921 aagtcaggac ttttcaagac tgtcacaaaa accatcgttt ttgatacatt aatttaacca

1981 ataggttgca gatcaaatcg cctgtaacag cctttctggc tgtttgtcgt tttcagaaga

2041 cggctgcact gaacgtcaga agccgactgc actatagcag cggaggggtt ggatccatca

2101 ggcaacgacg ggctgctgcc ggccatcagc ggacgcaggg aggactttcc gcaaccggcc

2161 gttcgatgcg gcaccgatgg ccttcgcgca ggggtagtga atccgccagg attgacttgc

2221 gctgccctac ctctcactag tgaggggcgg cagcgcatca agcggtgagc gcactccggc

2281 accgccaact ttcagcacat gcgtgtaaat catcgtcgta gagacgtcgg aatggccgag

2341 cagatcctgc acggttcgaa tgtcgtaacc gctgcggagc aaggccgtcg cgaacgagtg

2401 gcggagggtg tgcggtgtgg cgggcttcgt gatgcctgct tgttctacgg cacgtttgaa

2461 ggcgcgctga aaggtctggt catacatgtg atggcgacgc acgacaccgc tccgtggatc

2521 ggtcgaatgc gtgtgctgcg caaaaaccca gaaccacggc caggaatgcc cggcgcgcgg

2581 atacttccgc tcaagggcgt cgggaagcgc aacgccgctg cggccctcgg cctggtcctt

2641 cagccaccat gcccgtgcac gcgacagctg ctcgcgcagg ctgggtgcca agctctcggg

2701 taacatcaag gcccgatcct tggagccctt gccctcccgc acgatgatcg tgccgtgatc

2761 gaaatccaga tccttgaccc gcagttgcaa accctcactg atccgcatgc ccgttccata

2821 cagaagctgg gcgaacaaac gatgctcgcc ttccagaaaa ccgaggatgc gaaccacttc

2881 atccggggtc agcaccaccg gcaagcgccg cgacggccga ggtcttccga tctcctgaag

2941 ccagggcaga tccgtgcaca gcaccttgcc gtagaagaac agcaaggccg ccaatgcctg

3001 acgatgcgtg gagaccgaaa ccttgcgctc gttcgccagc caggacagaa atgcctcgac

3061 ttcgctgctg cccaaggttg ccgggtgacg cacaccgtgg aaacggatga aggcacgaac

3121 ccagtggaca taagcctgtt cggttcgtaa gctgtaatgc aagtagcgta tgcgctcacg

3181 caactggtcc agaaccttga ccgaacgcag cggtggtaac ggcgcagtgg cggttttcat

3241 ggcttgttat gactgttttt ttgtacagtc tatgcctcgg gcatccaagc agcaagcgcg

3301 ttacgccgtg ggtcgatgtt tgatgttatg gagcagcaac gatgttacgc agcagggcag

3361 tcgccctaaa acaaagttag atgcactaag cacataattg ctcacagcca aactatcagg

3421 tcaagtctgc ttttattatt tttaagcgtg cataataagc cctacacaaa ttgggagtta

3481 gacatcatga gcaacgcaaa aacaaagtta ggcatcacaa agtacagcat cgtgaccaac

3541 agcaacgatt ccgtcacact gcgcctcatg actgagcatg accttgcgat gctctatgag

3601 tggctaaatc gatctcatat cgtcgagtgg tggggcggag aagaagcacg cccgacactt

3661 gctgacgtac aggaacagta cttgccaagc gttttagcgc aagagtccgt cactccatac

3721 attgcaatgc tgaatggaga gccgattggg tatgcccagt cgtacgttgc tcttggaagc

3781 ggggacggac ggtgggaaga agaaaccgat ccaggagtac gcggaataga ccagttactg

3841 gcgaatgcat cacaactggg caaaggcttg ggaaccaagc tggttcgagc tctggttgag

3901 ttgctgttca atgatcccga ggtcaccaag atccaaacgg acccgtcgcc gagcaacttg

3961 cgagcgatcc gatgctacga gaaagcgggg tttgagaggc aaggtaccgt aaccacccca

4021 tatggtccag ccgtgtacat ggttcaaaca cgccaggcat tcgagcgaac acgcagtgat

4081 gcctaaccct tccatcgagg gggacgtcca agggctggcg cccttggccg cccctcatgt

4141 caaacgttat gcagccaaat cccaacaatt aagggtctta aaatggtaaa agattggatt

4201 cccatctctc atgataatta caagcaggtg caaggaccgt tctatcatgg aaccaaagcc

4261 aatttggcga ttggtgactt gctaaccaca gggttcatct ctcatttcga ggacggtcgt

4321 attcttaagc acatctactt ttcagccttg atggagccag cagtttgggg agctgaactt

4381 gctatgtcac tgtctggcct cgagggtcgc ggctacatat acatagttga gccaacagga

4441 ccgttcgaag acgatccgaa tcttacgaac aaaagatttc ccggtaatcc aacacagtcc

4501 tatagaacct gcgaaccctt gagaattgtt ggcgttgttg aagactggga ggggcatcct

4561 gttgaattaa taaggggaat gttggattcg ttggaggact taaagcgccg tggtttacac

4621 gtcattgaag actagtcctt tgcataacaa agccatcaaa ccggacgcca gagattccgc

4681 gcctgttgcg catggcttcg ccattttatg cgcaataggc gcgccaccct gtcgccgttt

4741 atggcggcgt taacccaaag gagtatcgtg aaaatatcac taatggctgc aaaagcaaga

4801 aatggggtta ttggctgcgg ctcggatatc ccgtggaacg ctaaaggtga gcagctgctt

4861 tttaaagcaa taacttacaa tcaatggctc ttagtcggcc gtaaaacatt tgaggcaatg

4921 ggggctctcc caaatagaaa gtatgcagtt gtcagccgct caggatcggt agctactaac

4981 gatgatgtgg ttgtgtttcc atctatagaa gcagcaatga gggagctaaa gactcttacg

5041 aaccatgttg ttgtttctgg tggtggagag atctacaaga gtctgatcgc ccatgccgac

5101 acgctacata tctcgacaat agattccgag ccagagggca atgttttctt tccggaaatc

5161 cccaaagagt tcaatgtggt gttcgagcag gaatttcatt caaatataaa ttatcgctat

5221 caaatctggc aaaggggtta accatccaag ccatcggaca cattttgctt cgctgcgctc

5281 aaaacgcaaa atgtgccgct gcttagcggc gttagatgca ctaagcacat aattgctcac

5341 agccaaacta tcaggtcaag tctgctttta ttatttttaa gcgtgcataa taagccctac

5401 acaaattggg agttagacat catgagcaac gcagtgcccg ccgagatttc ggtacagcta

5461 tcacaggcac tcaacgtcat cgagcatcat ctgggatcga cgttgctggc cgtgcatttg

5521 tacggctctg cactcgacgg tggcctgaag ccatgcagtg atattgattt gctggttact

5581 gtgactgcac agctcgatga gactgtgcgg caggctctgt tcgtagattt cctggaagtt

5641 tccgcttctc ccggccaaag tgaagctctc cgtgccttgg aagttaccat cgtcgtgtac

5701 ggcgatgttg ttccttggcg ttatccagcc agacgggaac tgcaattcgg ggagtggcag

5761 cgcaaggaca ttcttgcggg catcttcgag cccgcgacaa ccgatgttga tctggctatt

5821 ctgctaacta aagcaaggca acacagcctt gccttggcag gttcggccgc ggaagatttc

5881 ttcaactcag tcccggaaag cgatctattc aaagcactgg ccgacacctt gaaactatgg

5941 aactcacaac cggattgggc aggcgacgag cggaatgtag tgcttacttt gtctcgcatt

6001 tggtacagcg cagcaaccgg caagatcgcg ccgaaggatg tagctgccaa ctgggtaatg

6061 gaacgcctgc ccgtccaaca tcagcccgtg ctgcttgaag cccagcaggc ttaccttgga

6121 caagggatgg attgcttggc ctcacgcgct gatcagttga ctgcgttcat ttactttgtg

6181 aagcacgaag ccgccagtct gctcggctcc acgccaatga tgtctaacag ttcattcaag

6241 ccgacgccgc ttcgcggcgc agcttaattc aggcgttaga tgcactaagc acataattgc

6301 tcacagccaa actatcaggt caagtctgct tttattattt ttaagcgtgc ataataagcc

6361 ctacacaaat tgggagatat atcatgaaag gctggctttt tcttgttatc gcaatagttg

6421 gcgaagtaat cgcaacatcc gcattaaaat ctagcgaggg ctttactaag cttgcccctt

6481 ccgccgttgt cataatcggt tatggcatcg cattttattt tctttctctg gttctgaaat

6541 ccatccctgt cggtgttgct tatgcagtct ggtcgggact cggcgtcgtc ataattacag

6601 ccattgcctg gttgcttcat gggcaaaagc ttgatgcgtg gggctttgta ggtatggggc

6661 tcataattgc tgcctttttg ctcgcccgat ccccatcgtg gaagtcgctg cggaggccga

6721 cgccatggtg acggtgttcg gcattctgaa tctcaccgag gactccttct tcgatgagag

6781 ccggcggcta gaccccgccg gcgctgtcac cgcggcgatc gaaatgctgc gagtcggatc

6841 agacgtcgtg gatgtcggac cggccgccag ccatccggac gcgaggcctg tatcgccggc

6901 cgatgagatc agacgtattg cgccgctctt agacgccctg tccgatcaga tgcaccgtgt

6961 ttcaatcgac agcttccaac cggaaaccca gcgctatgcg ctcaagcgcg gcgtgggcta

7021 cctgaacgat atccaaggat ttcctgaccc tgcgctctat cccgatattg ctgaggcgga

7081 ctgcaggctg gtggttatgc actcagcgca gcgggatggc atcgccaccc gcaccggtca

7141 ccttcgaccc gaagacgcgc tcgacgagat tgtgcggttc ttcgaggcgc gggtttccgc

7201 cttgcgacgg agcggggtcg ctgccgaccg gctcatcctc gatccgggga tgggattttt

7261 cttgagcccc gcaccggaaa catcgctgca cgtgctgtcg aaccttcaaa agctgaagtc

7321 ggcgttgggg cttccgctat tggtctcggt gtcgcggaaa tccttcttgg gcgccaccgt

7381 tggccttcct gtaaaggatc tgggtccagc gagccttgcg gcggaacttc acgcgatcgg

7441 caatggcgct gactacgtcc gcacccacgc gcctggagat ctgcgaagcg caatcacctt

7501 ctcggaaacc ctcgcgaaat ttcgcagtcg cgacgccaga gaccgagggt tagatcatgc

7561 ctagcattca ccttccggcc gcccgctaaa tatctccttt tgggttgtta ataaaacatc

7621 caataagttg actgtgcgtg aaaaagaaag ttttgtgtga tggcgttgaa gatcgcaccg

7681 ttaagctctt atgtgggatg gtgcagagct cgacgactac cgataaaacg caaccgccgc

7741 aaacagacaa gaaaaagccc caactgataa cagttggggc ttcagtattg tgattggtgg

7801 agcaatagca ccctgaaccc aaaaccttct cgctcaaccg gtagtggctg ataacaactc

7861 gtgagggcta ttgcgggtta agcatttagc gatgtctagg gccagactgg acgtctgaac

7921 gcaagccgct gatactgtac ataaccacag tatcagcgga ggatacccat gtcgctggca

7981 aggaacgcca cggcgagtca atcgcccact caaacaaacg gttacgaacg ccaccaaccc

8041 gaccagacgc tgctctacca gctggttgag cagcactacc cagccttcaa agcctcactc

8101 gaagcccaag gtcaacacct gcctcgctac atccaacaag aattcaacga cctcctccaa

8161 tgtggccgtc tggagtatgg tttcatgcgg gttcgctgcg aggattgtca tcacgagcgt

8221 ctggtcgcct tcagctgtaa acgacgcggc ttttgcccta gctgcggtgc ccgccggatg

8281 gccgagagtg cggcgctgct gatagacgaa gtcttcccca aggagcccat tcgccagtgg

8341 gtgctcagct ttcctttcca gctacgcttt ttgctggctc gccatcccca gctgatgggc

8401 caggtcttga gtatcgtcta tcgtacactc tcaactcatc tgatcaaaaa agccggttac

8461 accaaagcct ctgcacaaac tggctcagtg actcttatcc aacgctttgg ctccgcgcta

8521 aatctcaatg tccactacca catgctgttt ctcgatggtg tctatgccga agatgactat

8581 ggcaagcaac gcttccatcg tgtcaaggca cccacttacg atgagctgaa tacgctcgct

8641 cacaccctca gccatcgcat cgctcgctgc atggaaaagc gtgggatttt ggagcgtgat

8701 gccgagaata cgtggttgac actggaagag ggcgaagacg atacgctgac tcaattacat

8761 ggtgcttcgg ttacgtatcg cattgccgtc ggcccccagc aagggcgcaa agtcttcacc

8821 ctgcaaacct tgccagggcg tgaggataaa gccgactcaa gcagtcgagt agccaaccat

8881 gctggtttct cgctacacgc cggtgtgatg gccgaagcgc atcagcggga taagcttgag

8941 cgcttgtgtc gctacattag tcggccagcg gtttcagaaa aacgtctggc attaaccgcc

9001 aatgggcagg tgcgttacga gctcaaaact ccgtaccgca atggcaccac ccatgtgatc

9061 ttcgagccgc tggacttcat cgccaaactc gctgcgttgg tacctaagcc gcgagtcaac

9121 ctcacacgct tccacggcgt ctttgcaccg aacagcaaac accgagttca agtaacaccc

9181 gccaagcggg gcaagaagcc cgacaaatcg gaaggtctcg atactaactg gcgtgacaag

9241 agtcctgcag agcgccaccg cgccatgacc tggatgcaac gcctcaagcg agtcttcaat

9301 attgatattg aagtctgcga acactgcggc ggtcacgtca aagtgattgc cagcatcgaa

9361 gatccgaagg tcattgagca gattctcaag catctgaaac agaaaacagc caaggcgaat

9421 gccgccaagc agcgtgagct gccaccagaa cgagcgccgc cactgactcc cagcctgttc

9481 gatccatcac agagtcgtct ctttgactga cgaccccaaa tccaacactg ctcaacactg

9541 ccaactttta aacggggcgg tggggcagtt tgtatctctc gagctatcag gctagagatt

9601 ttaccgccaa atcgaacctt attagagcgg tttaggctgg accggcagtt aaaattgggg

9661 cttgagcggt aaacgagtga gggaatttca ggtaagatac ttcggatgag gagcaaaaag

9721 gtggtttata cttcctatac ccggacgtca acaagtcgat ctatctggag ggcaagagcc

9781 cacagccgca ccgctgggag cctgccgagg gctggtttgc gaaatacgat cacccgctat

9841 ggaaacgcta cgccgatctg gcggcagggg ccgggcatgg cgggatggac tggttcgtga

9901 tccacgcttt tgtcgaggcg ctgaaggcca aggccccgat gccaatcgac atttacgacg

9961 cgctggcctg gagcgcgatc acgcctctgt cggaacaatc gattgctgag ggcaatcgca

10021 cgttagattt tcccgacttc acccgagggc agtggcgcac ccgcaagccg atctttgcgc

10081 tgaacgacgc ctattgatcg acgcgattta ggccaagcgc accgcaaagg cgaaattggt

10141 aatcttgtcg gtatccttga cgcccggcgc gctttcgacg ccgctggagg tatcgaccag

10201 cggcgctccg gtgcgcgcaa tcgcctcggc aacattcgtc ggatttagcc cgcctgccag

10261 cccccacggc aaggcaccgc gatatccggc cagcagcgac cagtcgaacg ccaaccccat

10321 gccgccgggc agcgcgcctt tgggggtctt ggcgtcgaac aagatcaagt ccgccgcccc

10381 ggcataggct gcggcgcgtg cgacatcgct ggcgctggcg acgggcagcg ccttccacac

10441 cggcttgcca aaccgcgcgc gcaactgggc cacgcgttcg ggcgattccg aaccgtgcag

10501 ctgcagcgcg ttcagcttgg ctgccaccag tgcgtcggcg atgacagcat catccgcatc

10561 gacgaacaaa ccgaccatgg cgatctggcc agctgcgcgc gatgtcaaag cgcccgcgac

10621 attcgacgta accgcacggg gcgacgctgg atagaacacc aacccggcat agtccgcccg

10681 cgccgcgatg gtcgcatcga gcgcctcggg tgtgctgatc ccgcaaatct tgattttcgc

10741 gggcatgcgg tcagtcgggg ttctggatca gccgcaccag cgtgcagtcg ggatcgatca

10801 ggtagccgat cctcaggccg ctcgcctcca gttgcggagc tttgaagcgc ggccagccgg

10861 tgcttttttc ctcggctccc gccgcgttca ccaatgccac catggcatcg agatcatcca

10921 accgcaggca acagccgaac gagctcgtag ctgggtcgag gtcaggatag gggaagaatt

10981 cgagctgcaa accgccgcgc tgcaggatca tccagccgcg atccttccaa ctcgtcgcaa

11041 agcccagctt cgcataaaac gcctctgtca catcgaaatc gcgcgatggc agattggggg

11101 tgacgtggtc agccatggct cagcgcagct tgtcggccat gcgggccgta tgagtgattg

11161 cggcgcggct atcgggggcg gaatggctca tcacgatcat gctggccttg gggaacgccg

11221 caccaaacgc gcgcgctgac gcggcgtagt gctcagtgtc ggcatcaccg agattgccga

11281 gcgacttggc cttgctgtcc ttgatcaggc agccaccaaa agcgatgtcg gtgccgtcga

11341 tcccaacggt gatattgtca ctggtgtggc cggggccggg gtaaaatacc ttgagcgggc

11401 caaagttggg cgcggttgct ggttcgaccc agccattggc ggcgaaagtc aggctgtgtt

11461 gcgccgcaac catcccctct tgcggggcaa gctggttcga caacgcattg gcataagtcg

11521 caatccccgc cgcatgcagc gcgtccatac cgcccatctt gtcctgatgc gcgtgagtca

11581 ccaccgccag cgcgaccggc aggttgatct cctgcttgat ccagttgagg atctgggcgg

11641 tctggtcatc ggtccaggcg gtatcgacca ccagcacgcg gccgccatcc ctgacgatca

11701 aaccgttgga agcgactgcc ccgaaacccg gcatgtcgag ataggaagtg tgctgccaga

11761 cattcggtgc gagctggcgg aaaaccagat cgccaaaccg ttggtcgcca gtttccattt

11821 gctggccaat cgtcgggcgg atttcaccgg gcatgcaccc gctcagcatc aatgcagcgg

11881 ctaatgcggt gctcagcttc gcgaccgggt gcataatatt gggcaattcc atcaagtttt

11941 ccttttattc agcattaaaa accccgcaaa tgcgaggcct agtaaataga tgatcttaat

12001 ttggttcact gtagcaaaaa tatggggcga attcaaacat gaggtgcgac agtttcaaaa

12061 gccatatgat aatcaacaag ctgtctttcg agtacggcat tagctggttc aattacagaa

12121 aatacgtctt ggaacaaaga gttctctgcc gaagccgtca atggtgtctt cgtgctttgt

12181 aaaagtagca gtaaatcctg cgctaccaat gacttagctc gtgcatcaaa ggaatatctt

12241 ccagcatcaa catttaagat ccccaacgca attatcggcc tagaaactgg tgtcataaag

12301 aatgagcatc aggttttcaa atgggacgga aagccaagag ccatgaagca atgggaaaga

12361 gacttgacct taagaggggc aatacaagtt tcagctgttc ccgtatttca acaaatcgcc

12421 agagaagttg gcgaagtaag aatgcagaaa taccttaaaa aattttccta tggcaaccag

12481 aatatcagtg gtggcattga caaattctgg ttggaaggcc agcttagaat ttccgcagtt

12541 aatcaagtgg agtttctaga gtctctatat ttaaataaat tgtcagcatc taaagaaaac

12601 cagctaatag taaaagaggc tttggtaacg gaggcggcac ctgaatatct agtgcattca

12661 aaaactggtt tttctggtgt gggaactgag tcaaatcctg gtgtcgcatg gtgggttggg

12721 tgggttgaga aggagacaga ggtttacttt ttcgccttta acatggatat agacaacgaa

12781 agtaagttgc cgctaagaaa atccattccc accaaaatca tggaaagtga gggcatcatt

12841 ggtggctaaa acaaagttaa acatcatgag ggaagtggtg atcgccgaag tatcgactca

12901 actatcagag gtagttggcg tcatcgagcg ccatctcgaa ccgacgttgc tggccgtaca

12961 tttgtacggc tccgcagtgg atggcggcct gaagccacac agtgatattg atttgctggt

13021 tacggtgacc gtaaggcttg atgaaacaac gcggcgagct ttgatcaacg accttttgga

13081 aacttcggct tcccctggag agagcgagat tctccgcgct gtagaagtca ccattgttgt

13141 gcacgacgac atcattccgt ggcgttatcc agctaagcgc gaactgcaat ttggagaatg

13201 gcagcgcaat gacattcttg caggtatctt cgagccagcc acgatcgaca ttgatctggc

13261 tatcttgctg acaaaagcaa gagaacatag cgttgccttg gtaggtccag cggcggagga

13321 actctttgat ccggttcctg aacaggatct atttgaggcg ctaaatgaaa ccttaacgct

13381 atggaactcg ccgcccgact gggctggcga tgagcgaaat gtagtgctta cgttgtcccg

13441 catttggtac agcgcagtaa ccggcaaaat cgcgccgaag gatgtcgctg ccgactgggc

13501 aatggagcgc ctgccggccc agtatcagcc cgtcatactt gaagctagac aggcttatct

13561 tggacaagaa gaagatcgct tggcctcgcg cgcagatcag ttggaagaat ttgttcacta

13621 cgtgaaaggc gagatcacca aggtagtcgg caaataatgt ctaactcaag cgttagatgc

13681 actaagcaca taattgctca cagccaaact atcaggtcaa gtctgctttt attattttta

13741 agcgtgcata ataagcccta cacaaattgg gagatatatc atgaaaggct ggctttttct

13801 tgttatcgca atagttggcg aagtaatcgc aacatccgca ttaaaatcta gcgagggctt

13861 tactaagctt gccccttccg ccgttgtcat aatcggttat ggcatcgcat tttattttct

13921 ttctctggtt ctgaaatcca tccctgtcgg tgttgcttat gcagtctggt cgggactcgg

13981 cgtcgtcata attacagcca ttgcctggtt gcttcatggg caaaagcttg atgcgtgggg

14041 ctttgtaggt atggggctca taattgctgc ctttttgctc gcccgatccc catcgtggaa

14101 gtcgctgcgg aggccgacgc catggtgacg gtgttcggca ttctgaatct caccgaggac

14161 tccttcttcg atgagagccg gcggctagac cccgccggcg ctgtcaccgc ggcgatcgaa

14221 atgctgcgag tcggatcaga cgtcgtggat gtcggaccgg ccgccagcca tccggacgcg

14281 aggcctgtat cgccggccga tgagatcaga cgtattgcgc cgctcttaga cgccctgtcc

14341 gatcagatgc accgtgtttc aatcgacagc ttccaaccgg aaacccagcg ctatgcgctc

14401 aagcgcggcg tgggctacct gaacgatatc caaggatttc ctgaccctgc gctctatccc

14461 gatattgctg aggcggactg caggctggtg gttatgcact cagcgcagcg ggatggcatc

14521 gccacccgca ccggtcacct tcgacccgaa gacgcgctcg acgagattgt gcggttcttc

14581 gaggcgcggg tttccgcctt gcgacggagc ggggtcgctg ccgaccggct catcctcgat

14641 ccggggatgg gatttttctt gagccccgca ccggaaacat cgctgcacgt gctgtcgaac

14701 cttcaaaagc tgaagtcggc gttggggctt ccgctattgg tctcggtgtc gcggaaatcc

14761 ttcttgggcg ccaccgttgg ccttcctgta aaggatctgg gtccagcgag ccttgcggcg

14821 gaacttcacg cgatcggcaa tggcgctgac tacgtccgca cccacgcgcc tggagatctg

14881 cgaagcgcaa tcaccttctc ggaaaccctc gcgaaatttc gcagtcgcga cgccagagac

14941 cgagggttag atcatgccta gcattcacct tccggccgcc cgctagcgga ccctggtcag

15001 gttccgcgaa ggtgggcgca gacatgctgg gctcgtcagg atcaaactgc actatgaggc

15061 ggcggttcat accgcgccag gggagcgaat ggacagcgag gagcctccga acgttcgggt

15121 cgcctgctcg ggtgatatcg acgaggttgt gcggctgatg cacgacgctg cggcgtggat

15181 gtccgccaag ggaacgcccg cctgggacgt cgcgcggatc gaccggacat tcgcggagac

15241 cttcgtcctg agatccgagc tcctagtcgc gagttgcagc gacggcatcg tcggctgttg

15301 caccttgtcg gccgaggatc ccgagttctg gcccgacgcc ctcaaggggg aggccgcata

15361 tctgcacaag ctcgcggtgc gacggacaca tgcgggccgg ggtgtcagct ccgcgctgat

15421 cgaggcttgc cgccatgccg cgcgaacgca ggggtgcgcc aagctgcggc tcgactgcca

15481 cccgaacctg cgtggcctat acgagcggct cggattcacc cacgtcgaca ctttcaatcc

15541 cggctgggat ccaaccttca tcgcagaacg cctagaactc gaaatctaac gtccgttcgg

15601 gcatcgaggt ccatgtcggg gtgggacggg cccgtggctt caagatcact tgcagtccga

15661 ccgcgatgtc ttggttgcgc gagaggttgt cgatatcctc cacttccatc atcaaccctg

15721 gataatgccg ccgccgtcat cgccgccgac gcccgtgccg ggcttttcgg gcctgtcagg

15781 cttgctcggc cttcagcctg cctgggcgag atctccggcg gacggattaa cggcggagct

15841 tcgccgcctt tcgtgcgtgt gaaggccgaa gatagttctc tcaaaaacat ccgtttatga

15901 gagataccaa atgtcatttt cagaagacga ctgcaccagt tgattgggcg taatggctgt

15961 tgtgcagcca gctcctgaca gttcaatatc agaagtgatc tgcaccaatc tcgactatgc

16021 tcaatactcg tgtgggctct gttgcaaaaa tcgtgaagct tgagcatgct tggcggagat

16081 tggacggacg gaacgatgac ggatttcaag tggcgccatt tccagggtga tgtgatcctg

16141 tgggcggtgc gctggtattg tcgctatccg atcagctatc gcgaccttga ggaaatgctg

16201 gcggaacgcg gcatttcggt cgaccatacg acgatctatc gctgggtcca gtgctacgcc

16261 ccggagatgg agaagcggct gcgctggttc tggcggcgtg gctttgatcc gagctggcgc

16321 ctggatgaaa cctacgtcaa ggtgcggggc aagtggacct acctgtaccg ggcagtcgac

16381 aagcggggcg acacgatcga tttctacctg tcgccgaccc gcagcgccaa ggcagcgaag

16441 cggttcctgg gcaaggccct gcgaggcctg aagcactggg aaaagcctgc cacgctcaat

16501 accgacaaag cgccgagcta tggtgcagcg atcaccgaat tgaagcgcga aggaaagctg

16561 gaccgggaga cggcccaccg gcaggtgaag tatctcaata acgtgatcga ggccgatcac

16621 ggaaagctca agatactgat caagccggtg cgcggtttca aatcgatccc cacggcctat

16681 gccacgatca agggattcga agtcatgcga gccctgcgca aaggacaggc tcgcccctgg

16741 tgcctgcagc ccggcatcag gggcgaggtg cgccttgtgg agagagcttt tggcattggg

16801 ccctcggcgc tgacggaggc catgggcatg ctcaaccacc atttcgcagc agccgcctga

16861 tcggcgcaga gcgacagcct acctctgact gccgccaatc tttgcaacag agcctccgtc

16921 gccatgctca cctcgctttg gtgcacacga gtattgagca tagtcgagat tggtgcagat

16981 cacttctgat attgaactgt caggagctgg ctgcacaaca gccattacgc ccaatcaact

17041 ggtgcagtcg tcttctgaaa atgacactgt ttgtatataa tcatgaaaaa atggtgagta

17101 gagtttcagg gtaacagggg atgcttatgt cggttttcca caactggcta cttgagatcg

17161 catgtgagaa ttacttcgtc tacatcaaac gcctttccgc caacgatacc ggcgcaacag

17221 gtggtcacca ggtagggctt tatatccctt caggtatcgt tgaaaaactc tttccgtcta

17281 tcaaccatac ccgtgaactg aacccttcgg tttttctcac cgcacatgtg tcatcgcatg

17341 attgccctga cagcgaagcc agggcaattt attataacag ccgtcatttt ggtaaaaccc

17401 ggaatgaaaa aaggattacc cgctggggta gaggcagccc acttcaggat cctgaaaata

17461 caggggctct gacgctcctg gctttcaagc ttgatgagca agggggggac tgtaaggaag

17521 taaatatttg ggtatgcgcc agcactgatg aagaggacgt cattgagacc gctattggtg

17581 aagttatacc cggagcgctt atatccggcc ccgcaggaca gattctaggc ggactatctc

17641 tacagcaagc gccagtaaat cataaatata ttctacctga agactggcac ctgcgctttc

17701 cgtcgggaag tgaaattatt cagtatgcag ccagccatta tgtgaaaaat tcccttgatc

17761 cggatgagca acttcttgac cgccggcgcg tggagtacga catatttcta ttggttgagg

17821 aactgcatgt tctggatatc atccggaaag gatttggctc tgtggatgaa tttattgcgc

17881 tggccaattc tgtcagcaat cgccgtaaat ccagagccgg gaagtcgctg gaactgcacc

17941 tggagcatct attcattgag cacggcctgc gacactttgc gacgcaggcc atcacagaag

18001 gtaataaaaa acccgatttc cttttccctt ccgcaggggc ttaccacgat actgagtttc

18061 ccgtagaaaa tctgcgcatg ctggcagtca agactacctg taaggatcgc tggcgtcaga

18121 tactgaatga ggccgataaa attcatcagg tgcatctgtt tacactccag gagggagttt

18181 ctctggctca atatagggag atgcgggagt cgggtgtcag attggtcgtg ccatcatctc

18241 tgcacaaaaa atacccggag gcggtgagag ctgagctaat gacgctaggt gcgtttattg

18301 ctgagctgac agggctttac gcagatattc catagattat ctcccggcat aaataccggg

18361 aggagcgatc agattcgttc aaccttgcac gaatcggcat taaccgcttt caggatataa

18421 ggttcaagca gtttggctac ggcttcaaac acgggcacca ctacggagtt accgaactgc

18481 ctgtacgact gagtgtctga cacaggaatg cgaaaaggcc tgccatctac tttttcaaaa

18541 cccataaggc gcgcgcactc tcgcggagtc agcctgcggg gccgatgcgc ctgattttct

18601 tcgttcgcga agtctgtttc acctgtggcc atatcccagc cacggtctat cagaatttca

18661 gacccgtctt tgtgatagcg agcagaaagc gtacgggcaa tgctttcttt attttcagga

18721 ttaacgaggc caaaaccgaa tccgttaccc ttagctgcgt gctttttggc gtagttatag

18781 agatactccc agagtttcgg cgtcagtata tatttgctgt caaccacggg ttccagcagt

18841 tcgccaaatg acggacgctg ttccggataa aaacgactaa tatcgcgcag ggtaaagccc

18901 tggtgaatgt tcagatcacg gcggaaaccg accaaaacga tacgttctcg gtgctgaggt

18961 aaaaagtgct ttccgtcgat aactttagga tcgtttttgc ccatctcagc tgcatccgca

19021 acttcatagc ccagttcgtc gagggtatcc atgatgactt taaaggtttt acccttgtca

19081 tggctcttca ggtttttaac gttttcaaga acaaagatgg caggtttttt tgcgcggata

19141 atacgcgcca catcgaagaa aagcgttccc tgagcctcac attcgaaacc atgcgcgcgc

19201 ccgagcgagt ttttcttgct tacgcccgca aggctaaacg gttgacaggg gaaacctgct

19261 agaagtacat catgatccgg cacatgctca ttaatgtaag cataggcatc gttttcaggt

19321 acttcaggtt tatcactgag cgtgacttca cgaatatcga gattgaaagt gtgttcctga

19381 gcatcgttaa accagttagc tttatatgtg cgcacagcct ctttattcca ttcactggta

19441 aaaacgcact ggccaccgat ggtttcgaag cccttccgta tacctccaat cccagcaaac

19501 aggtcaataa accggaaggc atagtcaggg tgatgtgcag gcgcttccgg aagcattttt

19561 cgtagaagtt cctcttcggc taacgtcagc gtcttaggtg agcacttacc attaatccag

19621 cgattaagag tctcgcgact ccactcattt ttaccaactt ttctaagcag ttcagccacg

19681 tacttctggt catagatttc cagcacctgc ccgagcagct ttttatcatt ttcctgtcgc

19741 agttgttctt ccgcttctgc tttctcaagc agatcctgcg ccagtaattc aaattcagac

19801 atattgcctc cattgggtct tatgggtgaa actgtatcac tcatttgacc cagattgaat

19861 gtttttatct ggatatttaa acaggtttat tgttaggtaa cgcacgttgg ccacgctgga

19921 gcgtcttctg ggcctgctgt cggcctttga ggtcgtggta tggatgacgg atggctggcc

19981 gctgtatgaa ccccgcctga agggaaagct gcacgttatc agcaagcgtt acactcagcg

20041 cattgagcga cataacctga atctgagaca acatctggca aggctgggac ggaagtcact

20101 gtcgttctca aaatcggtgg agctgcatga caaggtcatc gggcattatc tgaacataaa

20161 acactatcag taagttggag tcattaccgg ttctctttgt cttttagtga ttctataaac

20221 ctcattacgt ctgaatataa aaatctatta tttgatttat gtggctcatg aggttgtggg

20281 atggtcttgt ttttgaatgt gccagttttc ttaatggcaa agattaactc accttctgtt

20341 attcctaaca tttcagcaaa tgtttttgct tctatagtta ctgacttcat ttaattaact

20401 ctcatggtat cgattttctt taccggcatc tttaacaatg gtgctcgttt ctagtgttgc

20461 tgcggtacgc ttcatcatcg tctgcggggc ggttgcgata gtgaaggagc tgccgggcgt

20521 gagcaaatct atcaggcgct ggccgctgat aatctccatc cgttcactgg caatactgac

20581 agattttgaa cctgcgccgg ttttcccggt atggcaaaac agaccgcgac agttatgacg

20641 tttaagcaac ttctcgaact cctgtacgtg ctgtaaagca atatggccgc gatagcgttt

20701 agcctgaata agatagcgat attttcctat tattacctgg ccgtcaatgc ctccatcgcc

20761 ggtatagcgt ttgtttctga tggttctgaa gccatgcgct tcaaatcctt ccagcaacag

20821 ttcttcaaac acaaaaggat caattttcct caggtagtta attttttgtg ggaagcccgg

20881 caacgtcttt atgcgctcca gcacccgccg cgcactttgc tgcttccttt tgtgtcgcct

20941 gtgccgtact gaacgccgga agaatacaac ggcaagcagt gcgatggcgc tgcaagccca

21001 tagaataagg ttttctgtag tggggaaggg gatcatggtc atagtgggct ttctgagggt

21061 aaagaaaagg gcggttaaac cgccctggtg tttagcgacg gctgtatacc tgccacgaag

21121 cgctgcctga ctgattctgg caaatccgcc cgtaaagtac ggtgccggtc gagtagcggg

21181 cgccattcag atagcaatag ccgctggatt gcagcagttc gttgcgcagc ttttcttcct

21241 gctttgatag ccgggactcc gcagactgca aacgaacgga taattcgtta atctggcgtt

21301 gctggttatt cagctggctc tgcatcgcat tacctttatc ctggctgacg caaccagtta

21361 agagagctgt acaggctaat gcacttaata ttattttttt cacgttggct ccttaaattg

21421 agattattcc tagttcgcct ataagcgaac tttcccgtat ttacttatga tctggcttat

21481 catcgactgg ttacttccac cttcgccatt atccgggcat tcattaagaa aagccttcct

21541 ggcatccttc gtgtggttgg gtaaaaagcc gtgcttgttc tttttaacga tattgaagaa

21601 agcagcttca gcactgttac actcgcttcc gccgctatcg ccggtgagct tgcccgccat

21661 gcacataata actttgcagg gatcttcagc atggctggca ggaatataaa gcagactacc

21721 agctgctatc agaggtatta agagtttctt cattgttttg tccttaacag tttgttcaga

21781 tatacactcg ccagaatgtt gataacggta agcaatatta ataataacgc tgcgttataa

21841 atagatttgt aacctatatc gcctgcaata tattcgacaa caaaaacgaa aatcgttaaa

21901 attgtgtaaa acttgattaa ctttcctgta attttcttaa caatataagc aactgaataa

21961 gagccggatt tgatgatgct ggcgagtaac ctgatcgcat ggacacgaaa ttttctaaaa

22021 atgggcggaa tggaagatgc ctttttcttc cttctgcttt tttgcctttc cgcaaaatca

22081 ataagttcgc ccatttttat accctccggt tatatcttca gatcatcaag tgataaacca

22141 ctatcaagca gctctttgag ccatgtgggt cggcggccag tcccggacca ggtattttct

22201 gcgttcctgg ggtcgcggta ttttgctggt ctatgttctc tggggttggc tacgccctgc

22261 atcttcatgg cttccaggct aatgccagcg tccaattttg cagttagcca ggccgggcgc

22321 ttccctatac cagtccacgt attaaaaggg ttatccgggt cacgatacac gggttcgcct

22381 tttggtcgtt tttctgattt caggggaacc agcatcacct tccttccttc agcggttttc

22441 ttttcgtttc ttcttgacac gatctcatta gcccttatca taaacaaaag tcgtcgaaat

22501 tacaattacg cggtgaaaca taaatgagta aacatccaaa actcctggtt ctcgctctgg

22561 cctgccttgc ttgtgctggc cgtgccagtg ctgcgcctgc ctccgatgaa gttgccaggc

22621 ttgcgcagag atgtgcgcct gatgtttcac ccttaacgat ggcgtacatc gtcggccatg

22681 agtcctcaaa tgggccgtac aggatcaata ttaacggtag tattcagtta aaacagcaac

22741 cgcgtactga agctgaggcc gtcagcgttg cgaaagttct gctgaaggat aataaaagtt

22801 ttgatatggg ccttgcacaa attaactcaa ataatttagt gggcctgggt ctttcggttg

22861 acgatatttt caaaccctgc atcaacctgc gggcgagcca gaccatcctt aaagcctgtt

22921 atgatagcgc cctgaaatcc tacccagccg ggcaggttgc gctgagacac gcgctttcct

22981 gctacaacac cggctcactc acaaacggga tttctaacgg gtatgtcacg aaagttatca

23041 acgtggcgcg tcaatcaact gatttgaaaa tccctacgct gctacctgac ggccagacca

23101 gtgaggacag caccgcgact gagcctcagc aggcaaaaag tacggcgccg cagtatgacg

23161 gtgaacaaga tgtttttggt tcgggtgatg gcgatgcctt cagccgaaat aatacggatg

23221 cctttttaac cagacaggaa acagcgaagg gggaatgagg ttatggatgg aacgtttgta

23281 ccttgtattg caatcacaga tcctggagct atttgaagca ggaaaagtga aggaggtaac

23341 gatagaacgg gtttcattaa agaagtggta tcccgttttt cagatagacg atgaacagct

23401 gggccagatc gcatgttcca ttagggttaa caaagagcat gagctacgaa cctgggctga

23461 tttaaggcta ctggcagagt ttttgaaaga taagtgtggc gttgaagaat gccggttaaa

23521 tctgcaatca acagaagata gtgagtaagg agaaagtatg accacgttgt ttaagaagta

23581 tggccctgcg gtagttatgg gcgttttgtc cattgccctg ccgcaaattg cgctggccgc

23641 tggcaccgat actggtgaat caaccgctac atcaatccag acgtggttga gcacatggat

23701 tccaattggt tgtgctattg cgatcatggt tagttgcttt atgtggatgc ttcacgtaat

23761 cccagccagc tttattcctc gtatcgtaat ctcgctgatt ggtattggtt ctgcatcatt

23821 tctggtttcc ctgacgggcg taggaagctg aacaacgcga aaagggggga cttttgtccc

23881 ccaaagtgag gactacaaag atgttcgttg acgggaaaag accgcttttc aaaggtgcga

23941 ctcgcttacc tcgcgcgctg ggtgtaccac gtaatgtagc tatgatgata ttcatgattt

24001 ctgcctcgct ttttatgatt attcatatgt gggcgattct ggtgttcgtc tttttgtgga

24061 ttccttcagc tgcattaaca aaatatgacg accgcatgtt tcgaattatg ggcctgtggt

24121 tgaaaaccaa attcagtaat tggtttgatt ctccgtttaa gcagtgggga ggatcgtctt

24181 attcctctgt tgactacaaa cgtaagggtt taaaataatg agagctgcca ccgctacgaa

24241 gccaaaaaaa attgatgcct accgtaagga gccatcagta aataaaaagt atttgcccta

24301 ttcttatcac ctcaatgatt acgtgatttc gatggaaaac ggcgatctga tggctttttt

24361 caagctggat ggccgcacac atgactgcgc atcagatcgg gaactggtca cctggcataa

24421 agaccttaat acgctggtca agagcttcgg aacagaccat gtagagctgt ggacgcatga

24481 atatcaccat gaggctaaag agtacccgga tggtgagtat gaccattttt tccctgctta

24541 tgttgatcaa tataaccgta agctgcacgg tgattccaag cagctgatta atgaccttta

24601 tctgaccgtt atttacaaac aggtagggga taaaacacag aagtttctgg cgaaatttga

24661 aaagccgact cgtgacgaaa ttcagcaaat gcagaatgag gcgcttgaag gtctggaaga

24721 tatttctgaa caaatcctgg aagcaatgaa gccgtatggc attcagcagt tgggtatcta

24781 ttatcgtgac aaacgcggtg ttgaaattcc tgcgcctgat aaaaaagaac gtgaagaact

24841 tgctgaagtc gatgaatcag acatttttga cgaagccatt gttatcgaac gcaacgagcc

24901 tgaaccttcg caggctcacg cttattcaaa agcgctggag ttcctttatt tcctcgcaaa

24961 tatggaatgg gccatcgtgc ctgtttgccg tgatcgtatc cgtgagtaca tcatggacaa

25021 ccgccctgtt agctccctgt ggggggatgt tgtccagatc agaacggtag atcacaactt

25081 ctataccacc ggcattgaat ttcgtgaata cgaagaagat acagagccag gccagcttaa

25141 catgcttaaa gaagccgatt ttgaatacct tctgacgcag agtttttctt gcctctctga

25201 atcttcagct aaaacgtttc tgacgcatca ggaaaaatct ttgcaggaaa cgcgcgaccg

25261 tgcgcaaagc cagctggcac agcttggtac tgcgctcgat atgctgacgt ccagagagtt

25321 cgtgatgggc taccatcatg gaaccgtgca tgtctgggat aatgaccaaa acgcggtaca

25381 gcgcaaagcg cgtcgtgtga aggttatgct aaccggctgt ggcgtggttg gcgggactat

25441 cagcctggcc tctgaggctg catattatgc gagactgcct ggcaaccaga aatgggcgcc

25501 gcgcccggtt ccgataaact catggaactt cctgcacttc agcccgttcc acaattttat

25561 gcgtggcaag cctgacaata acccgtgggg gccagcgctg accatgttcc gcacgatcag

25621 cggtacgcca ctctatttta atttccatgt gaccccgctt gaagaacttt cctacggtaa

25681 acgcccgctg ggccatgcgt taataacggg tatgtcgggg gaaggtaaaa ccacgctgct

25741 taacttcctg ctggcgcagt caatgaagta caacccgcgg ctttttgttt atgaccgtga

25801 ccgcggtatg gagccgttca ttcgaagcgt tggtggctac tataaagttc tgcaacaggg

25861 tatgccgtcc gggtttgccc cgcttcagat tgaaccgacc aaacgcaata ttgccctcat

25921 taaaaacctg ttccgcattt gtgtggaaac caccaataac gggcctatca gcgcaacgat

25981 ggctaccgaa ctggctgaag gcgttgatgc ggttatgggg gaaggctcac ttattccacg

26041 cgaggcgcgc accgttacta tcctggacgg gtacgtgaat gaagttgtgg aaaatggcgt

26101 atcactgaaa gggctgctgc gcgaatggac gcgcgaaggc cagtatggct ggctgtttga

26161 caatgataaa gacagcctgg atctcagcgc gaatgatatt tttggcttcg atttatccga

26221 gtttatcgca gccaaagagg aagtatccag cccggcccgt actccgctca tgatgtacct

26281 tctgtaccgg gtacgtgact ccatcgacgg caaacgccgc gtcattcagt gctttgacga

26341 gttccacgcc taccttgacg atccggttat cgagcgtgaa gttaagcgtg gtatcaaaac

26401 tgaccgtaag aaagacgcta tctatgtgtt tgccacgcag gagccgaacg atgcgctgtc

26461 cagccgtatt ggccgcacga tcatgtcgca gaccgtcaca aaaatctgcc tgcgcgatcc

26521 ggaagctatc cgagaggatt atgccttcct tactgatgct gaatacgacg cgctgatgtc

26581 gattaccgaa cactccagac agttcctggt taaacaaggg caacagtctg cgattgcttc

26641 tttcaatctc taccctcgca acagcgacga tattgatgca gatattaaga caatggacaa

26701 cgttcttagc gtgttgtccg gtgaaccaca aaacgccgaa attgcgcatg agctggttga

26761 acggctcggt aatgaccctg aagtatggct caaagaatac tggcgcctga cggcttaaca

26821 acgaggcaaa acaccatgaa aaaaacactg acggcagtat tgctgaccac cggcctgatt

26881 ctgggaggcg cgcaaagcgc ttccgcaggc atcatcgtga ccaaccctac tgagctggct

26941 aaacaggtcg agcagcttca gcaaatggcg cagcagctgg agcagcttaa aagccagctg

27001 caaacgcaga aaaatatgta tgagtcgatg gcaaagacaa ccaacctggg cgatctgctg

27061 gggacgtcta ccagcacgct ggcaaataat ttgccggaca actggaagga gatctacagc

27121 gacgccatga actccagttc ttccgtcacg ccttcagtta acagcatgat gggccagttt

27181 aatgcggaag ttgacgacat gacgcccagc gaagcaatta cctacatgaa caaaaagctg

27241 gctgaaaaag gcgcttatga ccgcgttatg gcagaaaaag cctacaacaa ccagatgcag

27301 gaactaaccg atatgcagga gctgacggag cagattaaaa cgactccaga cctgaaatcg

27361 attgctgact tacaggcccg tatccagacg tcacagggtg ctattcaggg tgagcaggcg

27421 aagctgaatc tgatgaacat gttgcagcag tcacaggaca agctattacg tgcgcagaaa

27481 gaacgtgcca cccacaattt tgtttttgga accggcgggg acgttaccgc gtcaccttca

27541 attaactgag gtaattatga aaaaactact gcttgttatc cctttcctcc tggtggcctg

27601 cgatgcctcg catgacgtgg agtggtacaa aaaacatgag aaagagcgca aggcaacaat

27661 tcaggaatgc aagaaagacg cggatgaact tcagaaacct gattgcaaaa acgcgcgcga

27721 agccgatcgt cagctgtttg tgttcggcaa aaaagacggc gaaatcaatt caccgaaaat

27781 ttaggagtaa ggaggcaata tggcattcac cctagtcgca gacattttcg caaaagtaga

27841 cggggcgatt acgtcaatgg tgagcgccaa tgttgccacc attatctctg atgtaacgcc

27901 tctgattgcc acctgtctga caatcaagct gatggttcag gggatgtact cagcgtttaa

27961 tccgggggcg ggcgacagcc tgagttcgct gattaaagag tatctttcca tagcccttat

28021 cctgagcttt gcaacggcgg gcggctggta tcaacaggaa ctggtcaacg tggcgcttca

28081 cctgccggat gattttgccg ggatactgtc tgcccctaat aaagtcggtg caagtggcgt

28141 accggcgatt attgatagcg gtattgaaaa aggtatcaag atcgtcaaca ccgcatggga

28201 agccgcagac gtgttttcat cgagcggcct ggccgcgtat gccattggcg gcattatgat

28261 gattgctacc gttgtgctgg gcggcctcgg tgcgggcttt gtgatcatgg ctaagatcct

28321 tctggccgtt acgctttgtt ttggcccgat tgcaatcttc tgcctgctgt ggggagcgac

28381 aaaaaacatc tttgctcgct ggctggcgtc ggtcattaac tatggccttg tcgtcgtcat

28441 tcttgcgctc gtgtttggtt tcatcatgca gatgttcgac aacctcctgt cctcgatgaa

28501 ctctgatgcc gcttactcat caatcactgg ttctatctcc gccttattac tgacggtcat

28561 ttccgttttc gttctgttcc agattccgca aattgccgcc agctggggta gcggtatcag

28621 cgccggagtt gctgacgccg cacgctctac gggttcttcc atgcaggcgc ttggcaatat

28681 gggcagccac ggcatgtttg gcggtaatgc gttcagaggc ggtaacagtg gcggcggcca

28741 gcaatcggca ggtggaggaa gtggcagcaa cagcggagga agcagtggtt ctaatttaag

28801 tggtaaggca aggggcagtc gcgggaagaa ggctgcataa aattaaaaca aaagtcgttg

28861 aaattgcaat ttcggcgact tattataatt agtacgttca acaaccgata atggatgccg

28921 taatgcgcag cttattgctt atgggagttc ttctgattag cgcctgttcc agcgggcata

28981 aaccgccacc ggagccggac tggagcaaca ccgttccagt aaacaaaaca atcccggttg

29041 atacgcaagg tggtgcaaat gaaagctaat aaaaaaacag ggcttacacg tgaagccatt

29101 aaagagttca acgaaagccg taaagggctt gaagttgatc tgatggatga agtgctgaag

29161 tcccggcgta ccgcctggat ggttgccacc ggttcagcgg tggtaactgt ttttgcactc

29221 tctttagttg gttacgtggt gcataagtac agccagccaa tccccgcaca tctgctaacg

29281 ctcaacgagg ccactcacga agtacagcag gtcaagctga cccgcgacca gacctcttat

29341 ggtgacgaaa ttgataagtt ctggctgaca caatatgtca ttcaccgtga gagctatgac

29401 ttctattcag ttcaggtcga ctatacggcc gttggcttaa tgtccacgcc gaacgtggca

29461 gagtcttacc agagcaagtt caagggccgc aacggtcttg ataaggttct gggcgacagt

29521 gaaacgaccc gcgtgaagat taactctgtg atcctcgata aaccgcacgg cgtagcaacg

29581 atacgcttta ctacggttcg ccgcgtgcgc agcaatcccg ttgatgatca gccgcagcgc

29641 tggattgcca ttatggggta tgaatataaa tcgctggcga tgaatgctga gcagcgttat

29701 gtcaacccgc tgggtttccg cgtgacgagt tatcgcgtca accctgaagt taactgaggg

29761 ctgccccatg aaaaaactac ttctttcagc agtcgttttg tcagtcctgg gaggcgcggc

29821 cactaacgtt atggcgcttg aggttggccg caattctcct tatgactatc gcattaaaag

29881 cgttgtttat aaccctgtta atgtggtcaa aattgacgct atcgccggtg tggctaccca

29941 cattgttgtc gcgcctgacg aaacctatat cactcatgct tttggcgatt ctgaaagctg

30001 gacgtttgcg cacaaaatga accatttttt tgtgaagccg aaacaggcca tgagtgatac

30061 caacctggtg atcgtcaccg ataagcgcac ctataacatc gtcctccatt tcatcggtga

30121 agaaacgaag aaaaatgcag acggtacggt atcaaaatcc tttattgaaa cgccgtgggc

30181 tgtgcgccag gccgttcttc agctgaccta tgaatatccg tttgagcagc aggaaaaagc

30241 caaaagcgcg gctgataaaa aacgcattac gcagaagctg aagcagacgg cttttgcggg

30301 ggcgaagaac tatcagtacg taatgagcga acagcctgaa atgcgcagca tccagccggt

30361 tcacgtctgg gataactacc gctttacccg gtttgagttt ccggccaatg cggagttacc

30421 gcaggtctac atgatctcgg ccagtggcaa agaaacgctg cctaactctc atgttgtggg

30481 tgagaaccgc aacatcatcg aggtggaaac cgtcgctaaa gagtggcgta ttcgtctggg

30541 cgataaagtc gttggcgttc gtaataataa tttcgcgccg ggcgccggtg cggtagcaac

30601 cggtacggct tccccggatg tgcgcagggt tcaaattggg gaggataact gatggcccgt

30661 aaaagtgtcg atgtagatca ggaactcgat gaaaacaccg gagacggtga atttgaaagc

30721 gagcgtggcg gatttaaagg cagtaaccgc cgttcggctc ctggtatgaa agcctttgtc

30781 atactgatgg cgctgcttgc tttggtattc atcgggatta cggtcatggg taaaattcgc

30841 accccggcta aagctgaagc tgataaagac ggtggtaaag cgcaacaggc caatacactg

30901 ccaaactaca gctttaacag cgatcctgat gttaataaac ctgcaactgc gcagaatagc

30961 gccactgatg cccgtgctgt gcaggctgcc gcacaggcag atgcagatgc gggcagcagc

31021 aataccgccg cgcgtacctc taataagcgt aaagaacctt cgcctgaaga actggctatg

31081 cagcgtcgtc tgggcggcga gctggcccag actaatcagg cggctacaag caatagtccc

31141 ggagtgcagc cccaggacaa cgaaacaagc gaaggtagtt cagcactcgc taaaaacctg

31201 actcctgcaa ggctgaaggc tagccgcgct ggagtcatgg ctaatcccag cctgactgtt

31261 ccgaaaggca aaatgatccc ctgtggtacc ggcaccgagc tggataccac tgttccgggt

31321 caggtttcct gccgggtttc acaggacgtt tactcagctg atggactcgt taggctgatt

31381 gataaaggct catgggttga cgggcagatt accggtggta tcaaagacgg ccaggcgcgc

31441 gtgtttgttc tctgggagcg tatccgcaat gaccaggacg ggacaatcgt taatattgac

31501 agtgccggaa cgaactcact cggcagcgcg gggattccgg gccaggtgga tacccatatg

31561 tgggagcgtc tgcgtggtgc gatcatgatt tcgttgttct ctgacacctt aacggcgctg

31621 gttaaccaga cgcagagtaa taacattcag tacaacagca cagaaaacag cggtgagcag

31681 ctggcgtctg aagcacttcg ctcttacatg tctatccccc ctaccctcta cgatcagcag

31741 ggtgatgcgg tgagcatttt tgttgcccgc gacctcgatt tcagcggcgt ttatacgctc

31801 gcagacaact aaaaaagtgg gcgcttagcg cccgcttttc ttcaggagta atcatgactg

31861 atgcagcttt ctatcaactt ggcccactgc gcgagtattt agaagatcct actgtttttg

31921 aaattcgcat taactgcttt caggaagtta tctgtgatac gttcagcggc cgcagggttg

31981 tgcagaacgc ggcaattacg gcagatttta ttaggaacct tgctaaatcg ttggtgagca

32041 gcaacaagct gaccatgcag gccattaatg acgtgatcct gcctggcggg atcaggggcg

32101 ttatctgtct gccccctgcg gtgattgacg gtacaacggc cgtagcgttt cgtaaggatt

32161 tggcggccga taaaaatctg gagcagctga ccagcgaggg gattttcagt gactgccgga

32221 agattaccgg cagcaagcaa agcctaacgg atgatgattt tttccttaaa gagctgcaca

32281 gcagcgaaaa atggcccgca ttcctgcaaa ccgccgttga gaagaaacgc actatcgtga

32341 tctgcggtga aaccgggtcg gggaaaacgg tactcacgcg cgcgctgtta aaatcgctac

32401 ataaagacga gcgtgtaatt attttagagg acgttcacga agtcacggtc gatcacgttg

32461 tagaagccgt ttatatgatg tacggcgatg caggaaagat cggccgcgtc agcgccactg

32521 atgccctgcg agcctgtatg cgtctgacac cgggccgtat catcatgact gagcttaggg

32581 atgatgctgc gtgggattat cttaaagcac ttaataccgg ccatccaggc ggtgttatgt

32641 caacgcacgc taactctgcg cgcgatgcct ttaaccgtat tgggctgctt atcaaggcga

32701 cccctatcgg ccgtatgctc gatatgagcg atattatgcg aatgctctac tccaccattg

32761 acgttgtggt gcatatggaa aagcggaaaa tcaaagaaat ttattttgac cctgaatata

32821 aaatgcagtg tgtgaacggg agcctgtaat gaaaaactta gcaacctggc ttctggccgc

32881 agcatttacg acagccgccc tgcccgcctt tgcggtggaa ccatccgttc aggttggcta

32941 ctcgcctgaa ggaagcgccc gcgttctcgt cctgagcgcc attgactccg caaaaacctc

33001 gatacggatg atggcttatt cttttaccgc cccggatatt atgaaggcgc tggtagcagc

33061 caaaaaacgg ggagttgatg tgaaaatcgt aattgatgaa aggggcaata cggggcgcgc

33121 cagcattgcg gccatgaact acatagcgaa cagcggcatc cctttgcgta ctgacagtga

33181 tttccctatc cagcatgaca aggtgatcat cgtggataat gtgaccgttg aaactggcag

33241 ctttaatttc accaaagcgg ccgaaacgaa aaactcggag aatgcggtgg tgatctggaa

33301 catgcctaag ctcgctgaat cattcctcga acactggcaa gaccgctgga atcgggggag

33361 agactaccgt tccagctact gaatactgac cgccgaaagg cggttttttt tgttcaaaga

33421 aactgatctc agtatcgcgc acggggcagc tggccaacgt cgccggcatc gcgccggcaa

33481 aaatcgaaaa acagaaatat cagcaagata gcttaaacag gtgtcgggaa atttcgttga

33541 gatattttga aggaatcaca gtgtaagcag gcggcctatt tcaaaaaagg cggctgtacg

33601 gtagtcactg actctctcgc agatcttccc gctcatgtac cagtaatacc cgtcagtgct

33661 gacaatatcg agcaaatacc gcatatcata aaccgtatcg ggtattacct caccctcacc

33721 ggccagaacg ccgctggcaa tagaatggtt ccctcctcgt acaaagccta tccgccaggg

33781 caaccacaaa gtaacgcggt ggttaatatc ctgtacccac ggattgccct tagcgctgcc

33841 tatatcggct aaagcactcc ggtagcttga ttcatcccac ggccacggca ggatcttggc

33901 cgtcgcaagc gccaggggaa aatcttcagc tgcaagcctg agtgatttca tgtgcgtgta

33961 atccatcgcc cagatgattt ttgtgaagaa gaactcgcgc tcgttcatgt ccgggcgcgc

34021 gtcctggccc tccgtgccaa cggccagcaa ggggtcgtct cagaattcgg aaaataaagc

34081 acgctagcgg ttgatctgtc aggttgaagc ctgagaggcc gagcgcagat cgtcagaaaa

34141 ggcgaaaaac gatcctaatc tgacgcaaca taggtggggt gcctgacgcc cggttgaggc

34201 gtacttcaac tggacaccat tccagaaaga ccaagcatgg catggcctgc cgctgtctta

34261 ccgtgcttta tttcccgttt tctctatcga cccctatctc atctgcgcaa ggcagaacgt

34321 gaagacggcc gccctggacc tcgcccgcga gcgccaggcg cacgaggccg gcgcgcggac

34381 ccgcgccacg gcccacgagc ggacgccgca gcaggagcgc cagaaggccg ccagagaggc

34441 cgagcgcggc cgtgaggctt ggacgctagg gcagggcatg aaaaagcccg tagcgggctg

34501 ctacgggcgt ctgacgcggt ggaaaggggg aggggatgtt gtctacatgg ctctgctgta

34561 gtgagtgggt tgcgctccgg cagcggtcct gatcaatcgt caccctttct cggtccttca

34621 acgttcctga caacgagcct ccttttcgcc aatccatcga caatcaccgc gagtccctgc

34681 tcgaacgctg cgtccggacc ggcttcgtcg aaggcgtcta tcgcggcccg caacagcggc

34741 gagagcggag cctgttcaac ggtgccgccg cgctcgccgg catcgctgtc gccggcctgc

34801 tcctcaagca cggccccaac agtgaagtag ctgattgtca tcagcgcatt gacggcgtcc

34861 ccggccgaaa aacccgcctc gcagaggaag cgaagctgcg cgtcggccgt ttccatctgc

34921 ggtgcgcccg gtcgcgtgcc ggcatggatg cgcgcgccat cgcggtaggc gagcagcgcc

34981 tgcctgaagc tgcgggcatt cccgatcaga aatgagcgcc agtcgtcgtc ggctctcggc

35041 accgaatgcg tatgattctc cgccagcatg gcttcggcca gtgcgtcgag cagcgcccgc

35101 ttgttcctga agtgccagta aagcgccggc tgctgaaccc ccaaccgttc cgccagtttg

35161 cgtgtcgtca gaccgtctac gccgacctcg ttcaacaggt ccagggcggc acggatcact

35221 gtattcggct gcaactttgt catgcttgac actttatcac tgataaacat aatatgtcca

35281 ccaacttatc agtgataaag aatccgcgcg ttcaatcgga ccagcggagg ctggtccgga

35341 ggccagacgt gaaacccaac agacccctga tcgtaattct gagcactgtc gcgctcgacg

35401 ctgtcggcat cggcctgatt atgccggtgc tgccgggcct cctgcgcgat ctggttcact

35461 cgaacgacgt caccgcccac tatggcattc tgctggcgct gtatgcgttg gtgcaatttg

35521 cctgcgcacc tgtgctgggc gcgctgtcgg atcgtttcgg gcggcggcca atcttgctcg

35581 tctcgctggc cggcgccact gtcgactacg ccatcatggc gacagtgcct ttcctttggg

35641 ttctctatat cgggcggatc gtggccggca tcaccggggc gactggggcg gtagccggcg

35701 cttatattgc cgatatcact gatggcgatg agcgcgcgcg gcacttcggc ttcatgagcg

35761 cctgtttcgg gttcgggatg gtcgcgggac ctgtgctcgg tgggctgatg ggcggtttct

35821 ccccccacgc tccgttcttc gccgcggcag ccttgaacgg cctcaatttc ctgacgggct

35881 gtttcctttt gccggagtcg cacaaaggcg aacgccggcc gttacgccgg gaggctctca

35941 acccgctcgc ttcgttccgg tgggcccggg gcatgaccgt cgtcgccgcc ctgatggcgg

36001 tcttcttcat catgcaactt gtcggacagg tgccggccgc gctttgggtc attttcggcg

36061 aggatcgctt tcactgggac gcgaccacga tcggcatttc gcttgccgca tttggcattc

36121 tgcattcact cgcccaggca atgatcaccg cccctgtagc cgcccggctc ggcgaaaggc

36181 gggcactcat gctcggaatg attgccgacg gcacaggcta catcctgctt gccttcgcga

36241 cacggggatg gatggcgttc ccgatcatgg tcctgcttgc ttcgggtggc atcggaatgc

36301 cggcgctgca agcaatgttg tccaggcagg tggatgagga acgtcagggg cagctgcaag

36361 gctcactggc ggcgctcacc agcctgacct cgatcgtcgg acccctcctc ttcacggcga

36421 tctatgcggc ttctataaca acgtggaacg ggtgggcatg gattgcaggc gctgccctct

36481 acttgctctg cctgccggcg ctgcgtcgcg ggctttggag cggcgcaggg caacgagccg

36541 atcgctgatc gtggaaacga taggcctatg ccatgcgggt caaggcgact tccggcaagc

36601 tatacgcgcc ctaggagtgc ggttggaacg ttggcccagc cagatactcc cgatcacgag

36661 caggacgccg atgatttgaa gcgcactcag cgtctgatcc aagaacaacc atcctagcaa

36721 cacggcggtc cccgggctga gaaagcccag taaggaaaca actgtaggtt cgagtcgcga

36781 gatcccccgg aaccaaagga agtaggttaa acccgctccg atcaggccga gccacgccag

36841 gccgagaaca ttggttcctg taggcatcgg gattggcgga tcaaacacta aagctactgg

36901 aacgagcaga agtcctccgg ccgccagttg ccaggcggta aaggtgagca gaggcacggg

36961 aggttgccac ttgcgggtca gcacggttcc gaacgccatg gaaaccgccc ccgccaggcc

37021 cgctgcgacg ccgacaggat ctagcgctgc gtttggtgtc aacaccaaca gcgccacgcc

37081 cgcagttccg caaatagccc ccaggaccgc catcaatcgt atcgggctac ctagcagagc

37141 ggcagagatg aacacgacca tcagcggctg cacagcgcct accgtcgccg cgaccccgcc

37201 cggcaggcgg tagaccgaaa ggggtcgata gagaaaacgg gaaataaagc acggtaagac

37261 agcggcaggc catgccatgc ttggtctttc tggaatggtg tccagttgaa gtacgcctca

37321 accgggcgtc aggcacccca cctatgttgc gtcagattag gatcgttttt cgccttttct

37381 gacgatctgc gctcggcctc tcaggcttca acctgacaga tcaaccgcta gcgtgcttta

37441 ttttccgaat tctgagacga ccccagcaag taatcggcct gaattggcag tatcagcgcg

37501 cgtagtaagt catggatcgc cggttgcggc agtctgcgcg ccagattaat tacccggtcg

37561 aacttcagtt tatcctgctt gcgcttgtcg gtctgctcca tcagatttca tggccccctt

37621 cttcatgctc atgctcatgg gtgtgttctt ttccggtatg gctctgttcc gcctgagacg

37681 tctgcggcat ggcgtaatcg tcgtaaatgc tgctgtcaaa gtcgtagtct gatgcttcgg

37741 cataatgctc gtaatcggca tactcctgcg cgctccactg ctgatcgtcg gcagcagcat

37801 aatcatgggc cagttctgca tcattttgct gtgcttcatg acgccgcagg ccaacggaat

37861 catccatagg gttctgctta agatgaaagg cgtcctctgc gttgctcacc ggctgataat

37921 cagtgccggt tgtcatgtta tgttcatcgg gtttctggtt aaacgccatg ctttcccccg

37981 tggcttctgg cagacctttt tcagctgatc gggtttctaa actggtatcg cggccaatat

38041 ccttaaacct ggcctcaagc ccaaagaaac ggtcaatttc tgcggccgtg gttttcgggc

38101 tgtcgcggct cacgctcgat gccaaagatt ttttatcgtc ggtaaaaatt tccacctcat

38161 gacgcgcacg cgaaatacca acataaaaaa cgtccttaga agtggtaagc gatttggtat

38221 ctatgttgaa caacacgcga tcacaggtaa gcccctggga tttgtggacg gtggttgcat

38281 aagcatagga aagataagaa gcctgttttt tgtccagctc aaccgtgcgc ccttttttgt

38341 cctcaagcgt cagtttttca ccctccacgg ttttcaccgt gaagcggtcg ccgttggcaa

38401 cgtccagcgt tttatcgtta cgcgttacca taaccttatc gcccggcgcc agttcggcgc

38461 tgactgcctg gtatacagac agcttggtgt gtgtacgcgg gctgaaagcg atctgctcac

38521 cgctgctgct ttcaaccgtc aatttgttgc ccggcccggt atcaagaacc tggtaagact

38581 cgccccgctt cataccattt ttgtaatcct gttcggggat aatgatttgc cctttactga

38641 aataacggct gtcgcggcgt tccgcctgtg tcgaatccac gcggtcaagt agcgtgaacg

38701 tttcgccggt tccggcaagc cccagattgc cccggatgta gtcattgagg gttttgcgtg

38761 aggcgttcgt accagagatt atcagggtgg catcctgttg ttctgaggac agagacaggt

38821 agcgatcggc tagttgagag agtcggggcg cttcttcctt cagttcgttc acgccggtga

38881 tatttttcag ggcgcgcgcg gcattacctt cagcggcata cttaaccgcc tcaagcaaaa

38941 cttcattctt ctgtcgctga atgtctttca tgtagctggt ctgcatacct gctttaatca

39001 gctgctcaaa aggcttaccg gcttctaccg ctttcgtctg tgacgtatcc cccaggaata

39061 ccgcgcgagc gttatgcttc tcgatcacct ccatcagctg tttcatctgt cgggcgggta

39121 taaccccggc ttcatcaatg aatacgactg atttttcatc cagcttttta tccttcgctt

39181 tgaggaaagc ggcaacggtg cgggccggta atccatcatc ttcaagcgct tttttctgtg

39241 tcccataggg ggccagcgcc gtgaccttca gcccttgtga ctccagcagc tctttagcgg

39301 ccatcgtcat atagctttta ccggtaccgg cgtaaccatg tgcggccaca aaccgatctt

39361 tgctcgtcac aatttctgta accgcgcgca tctgctcctt cttgagggtt ttcccggcaa

39421 gcagctggcc tgcaatctct gcggtcagct gtcgcggcat ctgcccccgg ccgcgtgatt

39481 cgatagtcag aatggaacgc tcaaggcgaa taccctccac ggtagtgacg cggtggctgg

39541 tctttttaag cctgccgttt ttaataccat catctaccgc aaaacgggct ttatccgcac

39601 gcatcccgct attcgtcagc gagtcgatcc actctttgcg cgtcagagtt tcggccataa

39661 ctgaagcacc gaccttcaga gttgattgat accgggcttc gccctcgatg atggcgccct

39721 tctgtaccgc cttcaggtac gctttttcaa catcggctat tgtggcatgg cccagcacct

39781 gcttattagc gatttgaatc agcttctggc gttcaaagct ggcatcgcgc tctgacagcg

39841 acttaactgc aaactggata gcccggtcag ctttaacctc cgggctggta aaatccgggg

39901 ccatgttgcg cgctatatca gcctccagag gtttaccgtg tccctgccat tcacggttat

39961 caaaatcaat gccgagcgtt ttggcgcggc tggcccattc ctggtgaatt tcttcacggg

40021 aatgctctgt tttcttttca cgcgtagcca tcgagacgcg gcttttcgtc tgagcatcgg

40081 cggtttcccg cgtcagcccc attgcagcga gtcccttttc aatttgctcc gaccggcggg

40141 aaaaagcgcg aatctgttca tctgaaaaat gggccatatc gaacgtgtta tttttgctgt

40201 tgtaacgcag ctcataaccg gctttggtca actccaacgc cagctcctgt ttgtaaacat

40261 cgcccaggtg cattttatta cgcatcagct catcattttt gagcgcgcgc cactggccgt

40321 cctcgcgctg ggtcatgttc atgacaaaag cgtgtgtgtg caaatcagga tctagcgccc

40381 tggacgtttc gtggcggaaa gtagcgacga caaggttatt ggtattctgg gttactgatt

40441 tcccctggcg agtcgtccgg gcctgcgcga gtttttcagc ttcacgcaca gcagcggcaa

40501 cagctttttc atgagcctcg ataatggttt tatcgccgtg tatcagcgcc tgcatggata

40561 cccctttagg cgctgaaaac gtcaggtcgt agcccagacg ctcttttttg gcatcaccca

40621 cgtgtcgctg catatgcgtg aaggtatcta tctcaccgac aagcagctct ttaaaccggg

40681 ctgattcaac gtccccggat aagccgaggg cttcagctcc ggttccctgc caggacgtga

40741 atgatgaatc cttactgtag taatcatcct ttgcatcaga gtagtagccc acaacgctag

40801 tgacgttctg gcgggtaatc gtggttatat caagcatcag atatccctca gttcaatgcc

40861 aggaacaggg tttttgcgat ggtatttaac gtgtttagcc ttgaacttag cgacgggcat

40921 atcaccaggc aacgccagat agccggtgag gtttggcaac attgatattt cggtaggcgt

40981 tacggcacga acaactttaa cgtcgcggcg tttacggaca atccagggct tctgaggatc

41041 ggattcttta cgctcaactt cgccttctat ctcaccgagt gagcgcgaca tttgatccaa

41101 cgtttcatca ccgagacggc tgccgcccag cacgatgtta gaacgcatgt tagccagaat

41161 tgtctgagcc atatcccgac cataaacctt aaccagctga gaataggttt gatagccagc

41221 ataaacacac agaccgcttt tacgcccttt ggtcagtgca tcgttgaggt ttggcagaaa

41281 ctggagtgat tccagctcgt caataaatac attaatgcgg ctttcttttt cacccatacc

41341 cagcacgata gaaaaaatcg aatccagcca gcaggaaatt agcggattaa gtgacctttt

41401 catttcttcc tgccaggtga taaacaaggt tcccggcttt ccatcatcaa gccagtcacg

41461 cagggaaaaa ttaccttccg gcattttcaa atgtggggca agattcttac tgagaacaaa

41521 tcgcgcgctt ccaactgctt tttcagaccc ggaaaaaata gcttcggcag gcgtccccat

41581 taaaaattct tttaattttt tctggtcaac gttacaggcc cagtgaataa cttcttccat

41641 agttactgtg ctgtataggc tgtgaagttt tttcgaaact tcactaaaaa taagacggcc

41701 atagccgaac cattcttcag tagccatatc agggctttcc tgaacaatag agttcactaa

41761 gcgctcgtaa tcatatgaac ggcgaatttc attgaaaaac acccagcctt cagtgcgttt

41821 atcataggcg tttaaaataa catcgccggg acgatagaaa ttctttaaga accccccatt

41881 tggatctaaa gcaatatttt tgccgcctct aatgatgctc ttaaataaca gttcattgaa

41941 aattgtggtt ttaccagtac cggttgtacc ggcaatcgaa aaatgcaagt tctcagcgta

42001 tgtaggtatg gggatattag ccacggttaa ctggttgaca cctctttcgc gtgttttatc

42061 agcgagtgtt ctggcgctaa caagctctgt accacgataa atctttttga atctttcgcc

42121 tttaaacacg cgtgatttat cataaatgat aaaagcgatc agaccgccaa caccaataaa

42181 ccagccagca attaaagctg accataaagg ccatagcgaa aaagtattct taaccagata

42241 cggaatcagg tatttagccg tggatggatc aataccgtag gtaaattttg caactagaaa

42301 ccataccatc actggaggca aagtaattgc aaataaaaat gctaagcctc tttctctatc

42361 gtccatttca gcgctccttt tttggttccc agactttgta gccgttacgt tcaacctctg

42421 cttttgccgc tttggttttg cccggttctg ctatcgagcg caggaggatt agcgtttcaa

42481 tagcgagtga ttcatgcaac atcatctgtc ctgccggtgg gaattttacg ccagatagcg

42541 tttcggttat tgccttcagc tcatcgcgca gtgggccaaa atccgcatct gaagcacggt

42601 caaaaagata atccagtttg cgatttacgt cgctcagccg gtcggcaact attttcaacc

42661 cggactcccg atcacctggg ccagcttcaa tgcagcgccg cagataatct gaccgattac

42721 ctcctgaaac caggtctata taggccaaaa gttcatctga tacttttgcg gttattattg

42781 gcattcagtc ctcacattgt gcatttttta aacaaaaaat tgggatctaa caagctgaaa

42841 tcttagtatt accaaagtaa taaagcaaac tcattataaa acaatgagtt attgggtgtt

42901 tttaatacct aattattacc gaatattgtt gctatttatt tttttatctt ttaaatcagt

42961 atgatagcgt gatttatcgc gctgcgttag gtgtatagca ggttaaggga taaaaaatca

43021 tcttttttgg taggggcgat ctacgtaggt taaggactaa ctggctaaaa agcgttcaat

43081 attccgtatt catgcttgca tgaataccag tacaacaaaa gtacatcaaa attacatcaa

43141 aattacatca cttgaaggtt gacagtacaa caaaattaca tcattctttg gtcatgaggt

43201 agccagtaca acaaaagtac atcaaaagta catcaaaatt acatcaaaat tacatcattc

43261 taaatgaggg tactatgaag cccaaaagta tcagggcggc acttcagttg atgttgccgg

43321 aaatagaaga aatgctgtca ctgggagttt ccagggagga aatttataag gcagtttctg

43381 aacgcttcgg cctggaaggt gtgaacgttc gtagctttga tacgtcccta tatagagcgc

43441 ggcaaatccg gaaaaatgga atgcacaata cacatgaaag gatgccgaac aatgatgata

43501 gtgtattgca caatacacaa aaaggaggta gcgagaaagg tgcagaggaa agtgtattgc

43561 acaatacaca aacgccgcct gagcctgaac cgcaaggaag tgaaaaaaaa gaaagtcccg

43621 gcattattga taaagagttc ttcaataaaa tcagtgaaga tttcgaccct aagatgttca

43681 ataaaaaatt ctgaggtgat ttatgaaagt agcggtaatt aattacagtg gcagcgttgg

43741 taaaacctta atttcatcct acctgttagc cccgcgtctg actggagcaa aattctatgc

43801 ggttgaaact atcaaccagt ctgcttccga tctggggatt gagaatgttt ccatttttaa

43861 aggtgatgac ttttcacggt tgattgaaga tattgttttt gaagatgccg ggattattga

43921 tattggtgcg tcaaacgtag aagcgttcct gatggcaatg tcccgctttg acagtggcgc

43981 gaatgaattt gataagtatg taatcccggt tacgcctgat aataaggcca ttgatgaaag

44041 cctgaaaaca gcacatacgt taagtaaggc aggtgtaagc agcgataaaa tcatctttgt

44101 tccaaaccgc attagtccag atagtgaagt agaggatgta ctggcgccgg tatttgaatt

44161 tgtcaaacga acgaaagttg gcaaaatcag caagaaatct gttatttata acagtgaagt

44221 tttcgaatat ctcgcgtatc accgtatctc attcgaagca ttgaccgctg aagatccaga

44281 agaatttaaa gcccgcgcta aacaaacaac ggatgctgac gagcgcaaaa aactggcccg

44341 ccgttatacc tacatgaaac aggcaattcc tgttaaagct aatctcgata aagcatatgc

44401 ggctttaatg ggagaataaa atggaaaagc agccagataa atttgaagtt ctgatggatt

44461 ggtttttagg tgacgcgaag gaaatcaccg caagtcagaa agaaatgact gagatacttt

44521 ctgcgctttc ggaaaagctg gcaaaagaca ccgaaagttt aggagagacg gcagactctc

44581 ttaaacggac tttagtagaa aaccagcgtt caattagcct ggcaattagt gatgatgcta

44641 aggcgcgtga ggaatttctg acgaagttcc gccgcgcgca ggtgtccaga gctgagacgt

44701 taacccgtca gatccttttt attacagctg gctgcactat tgtgggcgcc gccgtgggtg

44761 ccgcgatagc cataattcta ctgagataat gtaaaccggg catgtcccgg ttttttttca

44821 agcgaagcgc ggaggccgca ggccggaggc attagtggcc gccgcccgaa ggggcgagac

44881 gcgtagcggc tcgatgcgca gcacggcaga acggccccgc aggggtaatg cccggtttaa

44941 ttcaacgtga cagtcacgtg gaggaaaaat atgaatgacc gacagcgtga acaagcccgt

45001 attcgccagg cccggcgccg cgcgcgactc aaagaggaag gcgctagcgt gacagtcacg

45061 ctaacaaaac aggaagaagc aatgttacag gagctttgcc gggttcgtcg tccaggacga

45121 acagcctatt ccacgaatga gtttttccaa ctgctgctga tccgaaactg gcaacagtgg

45181 caggagcaaa aggcgcagct ggggaaatgc caggcttgcg gaaagctgaa agcggaggga

45241 ggttgcggcg gcgaacggca gagcgaaacc tttaactgct ggctagccgt cgaagcaaac

45301 gagcttaatg tgtagtgtat tgtgctatac agaaattgcc aaaagcagcg cggagtaata

45361 actgaaacgg ccgcgcagcg gaaagaaaaa agcccggtca atccgggttt ttttctttcg

45421 gcggctcagt aatcgtctag atctccgcag cccagcgggc aacaattacg ctcaataccg

45481 tggctgcaat aatcgttttc ccgatcctgc cggatttctt ccgcgatctc gtcttgggtc

45541 agatagtcaa attcacgccg cgctaccgcg atcagttctt cccgatactc ggccggaacg

45601 ccagcaagat agccgtcaat aaccgcgctc aggcggcgct gatacagttc ataaatcagg

45661 ccgcagcggg gctgctgttc gcgcgcaaaa ctctcacact ggcgacgaaa atcttcaaga

45721 gtctgctcaa gggtttgtgt ggtcatgtcg tttgtcctcg atctttctgt taaagacccg

45781 gcttggccgg gtcgtcagtg ctatttagtt tgttgcagca gctggtctaa ttttgccgcc

45841 agtgcatacg tcgatgtgaa agcgccttgc aggcgctgat taggcagctg gttgaacgct

45901 tcgaggcagg cgcgcagcaa taattctttc tgagattcga cttctttttt cagctgggaa

45961 ggggtggtaa cagtggtcat gcttactcct tagtgatccg ataccggcaa tttttcgggt

46021 ggcggtattg cctcccgatg atttaattat cggtgattac gcctttaaag tcaatacaag

46081 tacggaattt atttacatgt ttttatgccc gtcagggcat ggaaggcgac cgcgccggac

46141 tccaccggac accggccgca aatcgccgga aactgcggga ctgaccggag caacaggcca

46201 accccccttc ctgctaagcc ataacccagc ccgcgcgcct gcgcgcgggt acagcggccc

46261 gcctgcgggt cgcggcgccg tactgcgagt tagcggccgc cgcgcggccg gttacggggg

46321 acaccgcacc gtcacggcca gcgccccgct gagctgcaca atccacggat aacacaatag

46381 cgcactggca aaggatgccg acgcctgaag ggcgttggca ccccgaaggg gcggggcgag

46441 acgggaaccg gctcgatgcg cagcacagca gagcggcccc gaaggggtaa cgccctgtgt

46501 ggcatcagga tttagcgcaa tggcagaaca tgagctggag agatcaccgg caagcagcag

46561 caaaggggcg gcgcagccgc ccagatggct gtttgccgat accggcgatt aattagagcg

46621 gtgtttaata tcccccgcgt ggcgggggac taggtttcag caagtcatgt taaatacgtg

46681 tccgtcatgt aaactgaaat ccccaataaa cagatcccgc gcataggcta cgatgtcaaa

46741 atatcgggct acggattcag gaatttcatt cagtagaccg ctatcattca ggtattcctc

46801 tgcaaaagtt tcttcgtcct tagcttcgcc catataggca tctctaaaca ggtcgaaatc

46861 agtgctatta aacagatcaa caaaggccac aaacgccgct tcgtttcctt cctcgcgggc

46921 ttgtttaaag ccgttaataa aatcccagtt gatatggcac tctgacgcca tatcagacgg

46981 aataccctcc caatcctgga acataaattc tggatcagcc tcatttgcgt gtaactcgcg

47041 gcagcgctcg taaaactcct ctgagctatc aaaatcggtc agatcgagcc aggctcccgc

47101 aatgcttccg cagttgtatt tatggtaagt gccaacataa acagaagggg tcgtaatatc

47161 agtcatggtg tactccttaa agcgccgata ccggcaattt ttcgggcggc ggtattgcct

47221 cccgatgatt taattatcgg tgattacgcc tttaaagtca atacaagtac ggaatttatt

47281 tacatgtttt tatgcccgtc agggcatgga aggcgaccgc gccggactcc accggacacc

47341 ggccgcaaat cgccggaaac tgcgggactg accggagcaa caggccaacc ccccttcctg

47401 ctaagccata acccagcccg ccgccacgca gctgccgcac gtcccccacg ggggtgcgca

47461 gtgggcgccg cgcgcctgcg cgcgggtacg gcggcccgcc tgcgggtcgc ggcgccgtac

47521 tgcgagttag cggccgccgc gcggccggtt acgggggaca ccgcaccgtc acggccagcg

47581 ccccgctgag ctgcacaatc cacggataac acaatagcgc actggcaaag gatgccgacg

47641 cctgaagggc gttggcaccc cgaaggggcg gggcggccgc ttgcggccgg gcgagtccgg

47701 cgcagggtgt ggcctgccaa gcggagcgcg gaggccgaag gccggaggcg ttagcggccg

47761 ctgcccgcgt aagcggggcg agacgggaac cggctcgatg cgcagcacag cagagcggcc

47821 ccgaaggggt aacgccctgt gtggcatcag gatttagcac aatgtcagaa cataaactgg

47881 agagatcacc ggcaagcagc tgcaaagggg cggcacagcc gccccgatgg ctgttacttg

47941 tctttgtcgc gtagcacttt gattaggccg gttacggccg taatcagagc ggccagcgag

48001 gtgatgattt gcggtaggtt ttcgaggatg gtagaggtca tatagcacct gtagagaagt

48061 tggcggggtg tcgtttccga cggccgcact gtaaccgggc gaataaggca ggttgtcaac

48121 agcttgagcg aagcgtctgt tgacaacctg ccgcgcccgg tttcactgcg gtcataggcg

48181 gaacgaccca cgccaacgga acggctttat gaccgggcag ctgagatacc ggcgaacctg

48241 gctggcggct gacgccagcc gccaagcgcc agcgcggagg gcgaagcccg gaggccaagc

48301 ggagcgcgga ggccgaaggc cggaggcgtt agcggccgct gcccgcgtaa gcggggcgag

48361 acgggaaccg gctcgatgcg cagcacagca gagcggcccc gaaggggtaa cgcccggagt

48421 ctgccgctgt ttatctctcg ttccatctga aatcggcggt aaggccatta aaagggtcag

48481 tttatcaggg gggcgttagc cccccatgtt gttaatcatc aggcaatatc gtctttgtag

48541 caggcataac cgaagctaag ctctgttttc atatagtggc gggcaaagtc ccaggcatcg

48601 tggccgaagt cctcataatc tgccaggacg atttcgcggg ctttgtgcca ttgctgtacg

48661 gaagaaagcg gcagaacagg gcagggaagc gaccagtcag tgacggtgtt gcagatcata

48721 tctgccagac gctccaggga gccgtaaacc agctctgtgc gcagatccgc caccatgcgt

48781 tgtttacgca gtgatgccag ataatcaatc tctttggtta tatcagaatt taagcgggtc

48841 tggtaatcca tgatgtactc ctttgcgcgc cgataccggc aattttgcgg gcgacggtgt

48901 tgcctcccga tgatttaatt atcggtgatt atgtcctcaa agtcaatata agtacggaat

48961 gtgcatacta aattttatat cttgcaaagc gttcatagag tgcctgaatc gctttctgac

49021 agcctcaata aaaaaggcgg ggattacccg ccttttttct tacagctgct tacgtggctt

49081 tttacgcgtc atatacaacg gtatcgcgca gtctaccgcg tacaaaaagc acgccagcgc

49141 gccgcaaccg tacagaaacg caagcggctt attatcgaag tagctgaaaa cccctgtcgc

49201 cgcacacagg cccagaacag agacgcaggc gcaggtgatc tgcaccagat ccctgtatcc

49261 cgcacgaacg ataaaccagg gcagggcaag cgcagcggcg ctaaaaatta atgcgagagg

49321 gacaaaaacg agatagtgat acatgtgaac tccttgatgg ttgccgatac cggcgattgt

49381 tcgggcggcg gtattgccac ccgatgattt aattagaggt tttgcgcgtc caggagattg

49441 acctgagccg gggtaacgtg aaacttttcc cctttatgga tcacgttatg cggggcgcta

49501 atttcatcac tgataaagct aaccgggtaa cgttttttac cgcaaatccg ctcgctaaac

49561 cacgccactt ttgccgctgg ccgatccact ggatgaacaa tcacaccggc catgctgcaa

49621 cccgttgcgg gttcgtccag cgtaatgctt accgggactg tatcccggta aaaaaactta

49681 accggcggca cacctgcctg cgtagcggct gcgacaactg caagcccgat aaccgctatc

49741 cgattaataa gcattttatt ccccttactc atgctgatat caccttgcca gctgttacca

49801 gtttacgaaa ttcgctttca tgaatttcac gcccatgctc aagcgttgaa tacacgttgc

49861 cgataagcca gttaccagcc gtttttgtct cggtatacca ccatgcttct gtcagagtaa

49921 gcagcggttc gctatcctcg cccttctcat agatccagat tttagtgacg tctttacccg

49981 tttcgttgct gccctgaatg gtagggtcaa aggtatgttc aatctctata tcgaagtaac

50041 gctgaaggaa gttagtaaag tgcatgacga ctcctgtaag cgccgatacc ggcaattttt

50101 cgggtggcgg tgttgcctcc cgatgattta attatcggtg attacgcctt taaagtcaat

50161 acaagtacgg aatttattta catgttttta tgcccgtcag ggcatggaag gcgaccgcgc

50221 cggactccac cggacaccgg ccgcaaatcg ccggaaactg cgggactgac cggagcaaca

50281 ggccaacccc ccttcctgct aagccataac ccagcccgcc gccacgcagc tgccgcacgt

50341 cccccacggg ggtgcgcagt gggcgccgcg cgcctgcgcg cgggtacagc ggcccgcctg

50401 cgggtccggc ggcccgcctg cgggtcgcgg cgccgtactg cgagttagcg gccgccgcgc

50461 ggccggttac gggggacacc gcaccgtcac ggccagcgcc ccactgagct gcacaatcca

50521 cggataatgc aggagacgaa tcatgatagg aggctgaagg ggaaatgagc ggcagcaggg

50581 gaaggggttg ccaagcggag cgcggaggcc gcaggccgga ggcgtcagtg gcagctgccc

50641 gcgtgagcgg ggcgagacgc gtagcggctc gatgcgcagc acagcagaac ggccccggag

50701 gggtgacgtc cgggggttcg cttttaaaga ttttcgacca catcagtaaa tcgtagtgac

50761 accatgaagc aaaagtatcg tgcagaccag aatcaacaac agtaacagca gacctttttt

50821 tcgacgtaat aaaaccggcc ccaaagcgcc cgcaagaagc acactcaaca cgatttccgt

50881 tatgctaatc gtattcataa tctctcacgt ttcccttttt agaactctgc cacacacgga

50941 taaaacctta taacaaagca acaaaacccg ttattcagac acggtaatgc ctagtttttt

51001 tgccagaatt tcagatgaca aagaaaccct tatcgacttc ccggttacaa ggtgaatgat

51061 tgtggcttca tgcccttcaa cagagtggga tattaactca cgctgaatga tatgcgtctc

51121 tccatcattt cccgtcacgg ctattaggct ctctgtagaa caggggttga tactactaat

51181 gttggtgttg ttttcggttt tgttcagcat cgctgatcct caaatatcgg tttgtgttac

51241 gtctgccgct ttgcgctgga taagcgactt aaagaaatcc gacgccttca gaatatcgct

51301 atcctggaag tcgggaaatg tcgcttctgt cagctcctgc gccgtacctg cccgtataac

51361 ggtttcatcg cggatcatcg ttcgcttaac gccatcagcc tgttttttgc tgaaggagtg

51421 ccggaaaata atcgcgtatt ggccgccgct atcctgatga acagaagtat cccagcgatg

51481 attaccctgt ttacgatagt ggtgatagat ttgctggccg tagcgctcct gatcggaatt

51541 acggtaatcg aacattaaca gttccaaaag cattaaccga tctggcaatt gccaggcggt

51601 agggtgttga agtgtcgcta acatagtttc ccctgagcgt gacagtcacg ataaggcggg

51661 ctttgcccgc ctggttatca gttaatcaat ggcacgataa atacgattct ggctttcgtt

51721 ctccagcgta ttaacgtact cccgcaacag gtgataccgg ttagccatcg tttcgttcag

51781 ttcggctttg ccttcttcat atgccagccc gcaaaagtaa ctgtaggcat acagacaaac

51841 aataatccct acttcgcgcg cgctgcattc accttcaaaa tagttaggta acgagagcca

51901 aagaggttgg ggcgcttcca taaaaaacgc gccattgctg gcctgaaggt attcccaata

51961 ccctccctgg tagtctttag cgtaacgatt cagaaaggac tgaatgaagt gatctgcgct

52021 gaagaaagcg ccacgaaatg ccgcaggcat gaagttcatg cgggcgtttt cagaaatgta

52081 gcgggcggtg atttcgatag tttccatgat acttcctctt taagccgata ccggcgatgg

52141 ttaagcggca ggcacatcac ctgccacttt ttaattatcg tacaatgggg cgttaaagtc

52201 aatacaagta cggattatat ttacctaatt ttatgcccgt cagagcatgg aaggcgacct

52261 cgccggactc caccggacac cgggggcaaa tcgccggaaa ctgcgggact gaccggagcg

52321 acaggccacc cccctccctg ctagcccgcc gccacgcggc cggttacagg ggacactgag

52381 aaagcagaaa gccaacaaac actatatata gcgttcgttg gcagctgaag cagcactaca

52441 tatagtagag tacctgtaaa acttgccaac ctgaccataa cagcgatact gtataagtaa

52501 acagtgattt ggaagatcgc tatgaaggtc gatatttttg aaagctccgg cgccagccgg

52561 gtacacagca tcccttttta tctgcaaaga atttctgcgg ggttccccag cccggcccag

52621 ggctatgaaa agcaggagtt aaacctgcat gagtattgtg ttcgtcaccc ttcagcaact

52681 tacttcctgc gggtttctgg ctcgtcaatg gaagatggcc gcatccatga tggtgacgta

52741 ctggttgtgg atcgctcgct gacggccagc cacggctcaa tcgtagtcgc ctgcatccat

52801 aatgaattta ccgtgaagcg gctactgctg aggcccagac cctgcctgat gccgatgaac

52861 aaagattttc ctgtgtacta cattgacccg gataatgaga gcgttgaaat ctggggagtg

52921 gttacgcatt cccttatcga gcatccggta tgtttgcgct gattgatgtc aatggcatgt

52981 acgccagctg tgagcaggca tttaggccag atctggcaaa ccgagcagtg gccgttttat

53041 ccaacaatga cggcaacatt gtggcccgta attacctggc gaagaaagcg ggcctgaaaa

53101 tgggcgatcc gtacttcaaa gtcagaccca taatcgagcg tcataacatc gctattttta

53161 gctctaatta cactctctat gcctccatgt cggcccggtt cgcggccgta gttgagtccc

53221 ttgcaagcca cgtcgaacag tattcaatcg acgagctttt tgttgactgc aaagggataa

53281 cggccgccat gagccttgac gctttcgggc gccaactgcg cgaggaagtc aggcgacaca

53341 caacgctggt atgcggggtc ggtattgccc gtactaagac gctggcgaag ctgtgtaacc

53401 acgctgcaaa aacatggccc gctactggcg gggtggttgc tctggacgat ggcgccagac

53461 tgaagaaatt aatgagcatc ctgccggttg cggaagtctg gggcgtcggc catcgtacag

53521 agaaagcact cgccacaatg gggatcaaaa cggtgctgga tttagccagg gcagatacgc

53581 gcctaatccg taagacgttt ggtgttgtgc ttgaaagaac ggtacgggag ctacgcggcg

53641 aggcttgctt cagcctggaa gaaaaccctc cggcgaagca gcagattgtt gtatcgcgct

53701 cattcggcca acgcgtagta gccctggcgg atatgcagca ggcgatcacc ggatttgcag

53761 cgcgcgcagc tgaaaaactg cgtaatgagc ggcaatactg ccgcgtcata agcgtcttta

53821 tccgcaccag tccttattca gtgcgtgata cacagtatgc caatcaggca accgaaaaac

53881 tgacggtggc aacccaggac agccgcacga taattcaggc ggcacaagcc gcgctggcgc

53941 ggatctggcg ggaagatatt gcgtatgcaa aagcagggat catgctggcc gatttcagcg

54001 gaaaggaggc ccagctggat ttattcgact ctgctacgcc ttcagctggc agcgaggcgt

54061 taatggctgt tctcgatggc ataaaccggc gtggcaagag ccagctgttt tttgcaggcc

54121 agggcatcga taactccttt gccatgcgcc gtcagatgtt gtcacctgat tacacgacag

54181 actggcgctc gataccaaca gcaaccatca aataattacc ggcgccgcac gcgggccggt

54241 caacccctca accggccgaa acgagtttcg gcgcggttga ggggttttcg gtaaaaggcg

54301 tttcatctgt ataaaagatc agctaaatta tgtgtattgc acaatacata tatgtgaggt

54361 taacagtgaa tttgcctacg cccgaaacct acgatgaact tcagagagcc tacgattttt

54421 ttaatgagaa gctattcagc aacgagctgc cgccatgcct gataacgttg cagcgtgaga

54481 agcgaacgta tggctattgt tcctttaagc gtttcgtcgg ccgtgagagt gggtacacgg

54541 tagacgagat cgctatgaat ccggtgtatt tctcgatcag aaccataaag gccacgcttt

54601 caacactggt gcatgagatg gttcatcagt ggcaattcca ttttggcgag cctggccgcc

54661 gtggctatca caacaaacag tgggcggccc ggatggaacg ggtaggacta atgccttctg

54721 ataccggcga accgggaggc aggaaagtgg gccagagcat gacccattat attattgccg

54781 gtggcccttt cgatatggcc tgtgatgaac tgctgacagg ccatttccgg ctttcctgga

54841 tggacaggtt tccgccttac cagcctaagc ctggcgctgt gctaagccct acaggaaaag

54901 gctatattga cgacgaggaa gatgatagcg aacacgaaca ggaggtggag gaagggcgcg

54961 acccggttga actcgacgac gagatcatag aggccatgcg atttgtaacc ccgccgcctg

55021 aagcgccggt gaacaaaaca aaccgggaaa agtacagctg cccggtgtgt catatcaatc

55081 tctggggtaa accggggata gtggtttact gtggtggcga gcactgtaat aaagccgcgt

55141 tagtagtctt aaaataaagt cctttcggac tttatttttt ttccatttcc gaggtcgtga

55201 tgttattaat gctgtacttc gcggcttctt ttaaaacagt ttcagcaagg cttgctggta

55261 tccagacctg aactaatttt aatggttcgc cgttctcggc tttaagagtg gtgttctggt

55321 acaaatccca gattcgctta acggtgctgg aaatgttttg cttggaacgg cctactcgcg

55381 tggctacgtc tgatgatttc tcacctttga caagcacgga atagccaata tctgttgtga

55441 tgtgtgcaaa ggaagccatt tgcggcagca gctgtttcca ttctgtttct gaaattctgt

55501 ttttctgagc catctgtggc gcctccgtag ttttggttac agaaaggata tactcagaat

55561 aaactggggt caatacaagt acgattttta taaactttat tttatttgag ggtgaggccc

55621 ggtgcggcag cagcgcgggc ctcgatggtg ccgcgaaggt gctggcgcca tgcttggatt

55681 aaaacatgaa ccgtgaagaa ctgcgaaact tgttttcgcg gttctgaggg gttgaccgag

55741 ccgcgaagcg gcgctggtaa gcgatgatat gcacatatcc acaggcatat ttttaaaagg

55801 tattttatag attttttatc tttttaaagt cttttagagc tatataactc attgatttaa

55861 aatcataaat aagtgttatc tctgggaatc cgcccacctt gttatgggaa ttggcccacc

55921 tatctatggg aaacacccca ccttactatg ggaattagcc caccttgtta tgggaattgg

55981 cccaccttag acgaaactgt aaaaaatgta tttacttgtt tgaactttgt ggtagtgtgg

56041 agagtaattt ttaacccaca aaggcaaggc tc

//
